# Application of quality by design for 3D printed bone prostheses and scaffolds

**DOI:** 10.1371/journal.pone.0195291

**Published:** 2018-04-12

**Authors:** Daniel Martinez-Marquez, Ali Mirnajafizadeh, Christopher P. Carty, Rodney A. Stewart

**Affiliations:** 1 School of Engineering, Griffith University, Gold Coast, Queensland, Australia; 2 Molecular Cell Biomechanics Laboratory, University of California, Berkeley, California, United States of America; 3 School of Allied Health Sciences and Innovations in Health Technology, Menzies Health Institute Queensland, Griffith University, Gold Coast, Queensland, Australia; 4 Centre for Musculoskeletal Research, Menzies Health Institute Queensland, Griffith University, Gold Coast, Queensland, Australia; 5 Queensland Children's Gait Laboratory, Queensland Paediatric Rehabilitation Service, Children's Health Queensland Hospital and Health Service, Brisbane, Queensland, Australia; University of Milan-Bicocca, ITALY

## Abstract

3D printing is an emergent manufacturing technology recently being applied in the medical field for the development of custom bone prostheses and scaffolds. However, successful industry transformation to this new design and manufacturing approach requires technology integration, concurrent multi-disciplinary collaboration, and a robust quality management framework. This latter change enabler is the focus of this study. While a number of comprehensive quality frameworks have been developed in recent decades to ensure that the manufacturing of medical devices produces reliable products, they are centred on the traditional context of standardised manufacturing techniques. The advent of 3D printing technologies and the prospects for mass customisation provides significant market opportunities, but also presents a serious challenge to regulatory bodies tasked with managing and assuring product quality and safety. Before 3D printing bone prostheses and scaffolds can gain traction, industry stakeholders, such as regulators, clients, medical practitioners, insurers, lawyers, and manufacturers, would all require a high degree of confidence that customised manufacturing can achieve the same quality outcomes as standardised manufacturing. A Quality by Design (QbD) approach to custom 3D printed prostheses can help to ensure that products are designed and manufactured correctly from the beginning without errors. This paper reports on the adaptation of the QbD approach for the development process of 3D printed custom bone prosthesis and scaffolds. This was achieved through the identification of the Critical Quality Attributes of such products, and an extensive review of different design and fabrication methods for 3D printed bone prostheses. Research outcomes include the development of a comprehensive design and fabrication process flow diagram, and categorised risks associated with the design and fabrication processes of such products. An extensive systematic literature review and post-hoc evaluation survey with experts was completed to evaluate the likely effectiveness of the herein suggested QbD framework.

## Introduction

### Current paradigm shift

An aging population exacerbating the current rate of bone related health complaints combined with increasing client expectations from bone surgical procedures will drive the push for implementing new technologies for innovative bone prosthesis and scaffolds. For biomedical companies to be competitive in the market, it is imperative for them to focus on continuous product development, and the customised needs of their customers. Custom products satisfy the specific individual needs for each patient [1], but is excessively expensive using traditional manufacturing methods [2]. However, the recent advent of additive manufacturing (AM) technologies also known as 3D printing is providing a new path for the design and manufacture of custom medical devices.

### Additive manufacturing

AM is an emerging manufacturing technology capable of fabricating complex shapes, and manipulating material properties that are impossible with traditional manufacturing methods. This technology is supported by CAD software to build 3D physical models from a series of cross sections that are automatically joined together to create the final shape [3]. The best known AM methods in the biomedical field are stereolithography, selective laser sintering, Inkjet 3D printing, electron beam melting, polyjet photopolymer and fused deposition [4, 5]. There are a variety of materials that can be used by these AM methods such as plastics, ceramics, metals and living cells [3, 6]. These materials are used by AM in the form of powders, filaments, and liquids [7].

3D printing is rapidly growing in popularity in the biomedical field for the development and design of customized bone prostheses and scaffolds [8–11]. To achieve accurate and safe bone solutions, 3D printing manufacturing needs to be integrated with other technologies such as digital medical imaging, material science, CAD, Finite element analysis (FEA), nano surface modification, motion capture, virtual surgical planning, and also with concurrent collaboration of experts from different fields [12].

Custom 3D printed bone prostheses and scaffolds have to overcome several barriers before being released to the market. Some of these barriers are that medical devices are strictly regulated by organizations such as FDA (USA), EMA (European Union), and TGA (Australia), in order to ensure their compliance to medical specifications and consistency in manufacturing practices. Moreover, current standardization methods applied to traditional manufacturing methods are not suitable for 3D printing technology [13]. As a result, there is a lack of standardization and defined quality control processes [14]. Furthermore, additive manufacturing incorporates new technologies in the biomedical field, and the design and fabrication of custom bone implants requires many steps that might lead to imperceptible errors, affecting the performance and consequently, patient safety. Successful industry transformation to this new design and manufacturing approach requires technology integration, concurrent multi-disciplinary collaboration, and a robust quality management framework.

### Product development: Avoid the ‘valley of death’

Product development involves numerous changes, iterations, and evaluations to achieve final product concept and design [15]. Early stage design can comprise 70% of the total product life cycle and influence between 70% and 85% of the total product cost [16]. For medical devices, higher development costs reflect inherent complexity and risks plus the clinical testing required by medical regulatory organizations [17, 18]. Tissue engineering ventures are susceptible to insolvency from a “Valley of Death” funding gap, due to costly pre-clinical and clinical studies for safety assessment before clinical approval [19]. Consequently, despite large investment, only a relatively small percentage of new tissue engineering research achieves clinical application and market release [19].

In order to help to close this gap, research studies should focus on technologies and processes that have the potential to be scalable, and designed for a particular clinical application that can achieve regulatory approval, gain surgeons acceptance and guarantee insurance coverage [20]. However, during the early stages of product development, the chances of a risk event are most likely to occur [21]. Therefore, efforts and resources to reduce and control product development risks should be expended at the early development stages, where the cost of a risk impact is less than if it takes place in other phases of the product life cycle [21]. Moreover, new developments should be systematically designed, manufactured and tested [22], fostering rational design approaches and avoiding trial-and-error studies [23] to also accelerate research timelines and reduce development costs [24]. Additionally, product success is directly influenced by its predevelopment activities such as preliminary market assessment and technical analysis [25].

### Quality by design for 3d printed bone implants

The concept of QbD was first created and published in 1985 by Dr. Joseph Juran to build quality during the development of products and services [26]. Juran’s ideas were later adopted by the US Food and Drug Administration (FDA), and the International Conference on Harmonisation (ICH), in order to create a flexible regulatory framework to improve pharmaceutical manufacturing processes and enhance pharmaceutical product quality [27]. This framework includes the ICH harmonised tripartite guidelines (ICH), incorporating guidelines ICHQ8(R2), ICHQ9, and ICHQ10. The QbD approach is composed of eight main steps that follow in a systematic way they can provide a deep understanding of the product and its manufacturing process, including the identification and control of all variables to ensure desired quality. These eight steps are: (1) Quality target product profile (QTPP), (2) critical quality attributes (CQA), (3) process flow diagram (PFD), (4) critical process parameters (CPP) and material attributes (CMA), (5) risk assessment (RA), (6) design space (DS), (7) design and implement a control strategy (DICS), and (8) development of strategies for product lifecycle management and continuous improvement (PLMCI).

Quality by design (QbD) is an approach where the product is carefully designed, with consideration provided to all aspects of its life cycle. Thus, the product is designed correctly from the beginning [28]. QbD is a tool that uses science and quality risk management to acquire a deep understanding of products and processes and eventually process control [29]. QbD philosophy is focussed on building quality into the product development process [30], with the main activities performed concurrently using multidisciplinary efforts, thereby providing immediate feedback [28]. Moreover, waste, time and cost can be reduced through early detection of errors and mistakes during the design and fabrication processes of 3D printed products [31]. Furthermore, the implementation of QbD can help to reduce the regulatory burden that forces many product engineers to purposely design their products to fit within existing approved thresholds in order to avoid seeking further time consuming approvals for minor variations [32].

## Purpose and objectives

QbD encourages process and product understanding to support innovation and efficiency in product development. Moreover, the application of a QbD approach helps to meet FDA regulatory requirements [32]. The benefits of QbD can be translated into an acceleration of product development, and a reduction of costs and waste. Therefore, the purpose of this study is to adapt the QbD approach to early stage design of custom 3D printed bone implants considering the ICHQ8(R2) guidelines [33] and existing quality risk management tools. Hence, the current study sought to achieve the following three main objectives:

1. Identify the main applications and benefits of a QbD approach as described in previous research studies.
2. Develop a comprehensive design and fabrication process flow diagram of custom 3D printed bone implants.
3. Identify and categorise the risks associated with the design and fabrication processes of such products to facilitate further risk assessment analysis.
4. Develop and validate an adaptation of the QbD approach to early stage design for custom 3D printed bone implants.

## Scope

**Fig 1:**
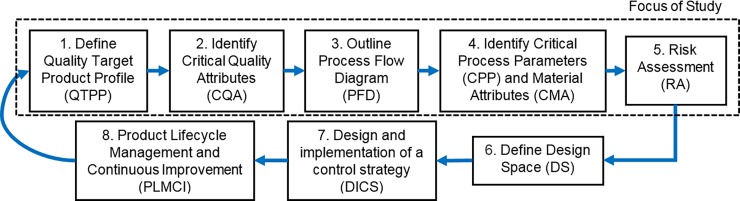
QbD systematic product and process design development flow chart showing principal steps. Adapted from [27, 34].

Fig 1 illustrates the focus of this current study within the overall systematic eight step QbD approach for the development of process and product design. Specifically the scope of this present study is limited to:

- A statistical analysis of 30 peer reviewed journal papers to identify the main reasons why QbD had been used in different health related research fields, including their main outcomes and benefits.
- The implementation of the first five steps (i.e. Steps 1–5) of the QbD framework for the early design stages of custom 3D printed bone implants.
- A general QbD approach is specified for each element within each of the five steps; detailed specifications for different bone implant topologies are outside the scope of the present study.
- The Risk Assessment step (i.e. Step 5) is limited to the identification and categorisation of risks associated with the quality of such products during their design and fabrication.

## Materials and methods

This is a qualitative exploratory research study that is focused on the adaption of the QbD approach to custom 3D printed bone implants. To the authors’ knowledge, no previous work has investigated, discussed and/or proposed implementation of the QbD approach to 3D printed bone implants. We propose a constructive research approach to produce innovative solutions to practical problems in a heuristic manner, followed by validating the solution afterwards [35]. The solutions are commonly proposed using managerial problem-solving techniques through the construction of models, diagrams, plans, and organizations [36]. The recommended solutions requires the researchers to immerse themselves in the contextual literature to allow an in-depth interpretation and synthesis of the problems [35].

According to Oyegoke (35), the constructive research approach comprises six phases which are covered in this study in the following manner:

- *Phase one*: Involves finding a relevant practical problem that has strong research potential. This study aimed to fill a gap in knowledge by formulating a QbD for the development of custom 3D printed bone implants.
- *Phase two*: Focuses on creating topic understanding, which was achieved by completing a comprehensive literature review, followed by a systematic development of a specific QbD system tailored for custom 3D printed bone implants.
- *Phase three*: Involve innovating and designing a new construct and was achieved through the design of a unique QbD system for the development of custom 3D printed bone implants. The authors are not aware of previous reported work that has investigated, discussed, and proposed a QbD system for custom 3D printed bone implants.
- *Phase four*: This phase should demonstrate that the new construct (solution) works. This research will demonstrate that a systematic implementation of the QbD system for 3D printed bone implants can provide a more comprehensive understanding of the design and manufacturing processes of relevant products through providing a detailed workflow map of these processes. This workflow map will lead to a more accurate identification of the risk sources and its potential effects on product quality, through the development of a Risk Breakdown Structure.
- *Phase five*: Focuses on the theoretical connections and the research contribution of the solution concept. The new novel QbD approach proposed in this study is supported by various different datasets obtained from peer-reviewed papers. Moreover, the reliability of this study is based on design and fabrication methodologies, and lessons learned that have been used successfully in different studies for the development of 3D printed bone implants.
- *Phase six*: Semi-structured interviews were completed in order to solicit opinions from pertinent experts on the applications and benefits of QbD for 3D printed prostheses and scaffolds.

### Data collection

Three systematic searches were conducted based on objectives 1, 2, and 3 and according to the Prisma statement [37]. The first systematic search was performed in Science Direct on 2^nd^ August 2017. Search term was related to studies implementing the concept of Quality by Design based on objective 1. The search related term was: “*quality by design*”.

The second and third systematic search were based on the objectives 2 and 3 defined above. Searches of Science Direct and Google Scholar databases was conducted on 10^th^ November 2016. Search terms were related to AM of bone implants and the risks associated to product quality during their design and fabrication.

The terms related to objective 2 were: Additive manufacturing; 3D printing; rapid prototyping; reverse engineering; tissue engineering; and biomimetic. These terms were connected to independent keywords using Boolean operators (AND, OR) in order to narrow down search. The connected independent keywords were: scaffold(s); custom(ized, ised); patient specific; implant(s); prosthes(is, es); design; and lattice.

The full search phrase used for objective 2 was:

("Additive manufacturing" OR "3d printing" OR "rapid prototyping" OR "reverse engineering" OR "tissue engineering" OR "biomimetic") AND (scaffold* OR custom OR custom?ed OR "patient specific") AND (implant* OR prosthes* OR design OR lattice)

The terms related to objective 3 were: "Additive manufacturing; 3D printing; rapid prototyping; and reverse engineering. These terms were connected to independent keywords using Boolean operators (AND, OR): accuracy; defect(s); metrology; quality; errors; optimi(zation, sation); strategy; and rules.

The full search phrase used for objective 3 was:

("Additive manufacturing" OR "3d printing" OR "rapid prototyping" OR "reverse engineering") AND (accuracy OR defect* OR metrology OR quality OR errors OR optimi* OR strategy OR rules)

Interviews are one of the most common techniques to gather primary data in all type of business and management research [38]. Interviews can be used to extract expert knowledge about their experiences, beliefs, or opinions [39]. Therefore, in order to confirm the applications and benefits of the adaptation of QbD, qualitative semi-structured interviews were selected as the data collection instrument to be performed with each interviewee. For the purpose of this study, the exploratory approach was adopted following the consolidated criteria for reporting qualitative research (COREQ) [40]. By following this approach, 26 questions in total divided in four specific groups were designed. Moreover, an interview guide and a PowerPoint presentation were designed to guide the direction of the conversation, present the preliminary results obtained from the adaptation of QbD and the statistical analysis, and to gather deeper insight on matters that could not be taken into account during the systematic search. (See supplementary material in S2 File for complete details of this procedure).

### Study selection

Selected studies from the first systematic search were limited to the following inclusion criteria: (1) peer-reviewed papers with full-text; (2) empirical studies showing evidence of the applicability of Quality by Design approach; (3) published in English language; (4) assess the first ten pages of the search results; (5) sort the search results by relevance.

Selected studies from the second and third search were limited to the following inclusion criteria: (1) peer-reviewed papers with full-text published within the last 16 years (2000–2016); (2) empirical studies showing evidence of the applicability of design and manufacturing methods; (3) reports describing errors and difficulties experienced during any step of the design and manufacturing process; and (4) published in English language; (5) assess the first one hundred thirty pages of the search results; (6) sort the search results by relevance.

The criteria to select the participants for this post-hoc QbD evaluation study was based on their experience and expertise in the field of study. Therefore, pertinent experts in the field of tissue engineering, medical product development, and orthopaedic surgeons with previous experience with 3D printed bone implants, were selected. The sample size is limited by the nature of the research field, which is characterized by small samples, but detailed and extensive work [41]. Therefore, the snowball sampling method was selected since it allow further study participants to be suggested or introduced from the interviewees network [39].

### Data extraction and analysis

For the systematic review, full-text screening was independently performed by the authors. Any discrepancy between the two reviewers was resolved by a consensus meeting. The articles were classified based on their research objectives to facilitate their analysis. The classification topics were: quality by design, properties and requirements of porous scaffolds; medical image; biomaterials and surface treatments; study cases; 3D printing methods; 3D printing fabrication errors; design approaches; and performance simulation (finite element analysis; and joint kinematics simulation). The classified articles were thoroughly reviewed and analysed according to the objectives of this study. From *search 1* the following information was extracted: article application context, QbD implemented steps; QbD tools used; Key output/conclusion. Moreover, the articles’ key output/conclusion was classified in different four categories: (1) Process understanding (PU); (2) Prediction and optimization (PO); (3) Reduction of experimental runs (RER); (4) Development of robust manufacturing methods (DRM). Furthermore, the total number of experiments that each study performed was gathered, including the calculation of the number of experiments if they had used one variable at the time.

**Fig 2:**
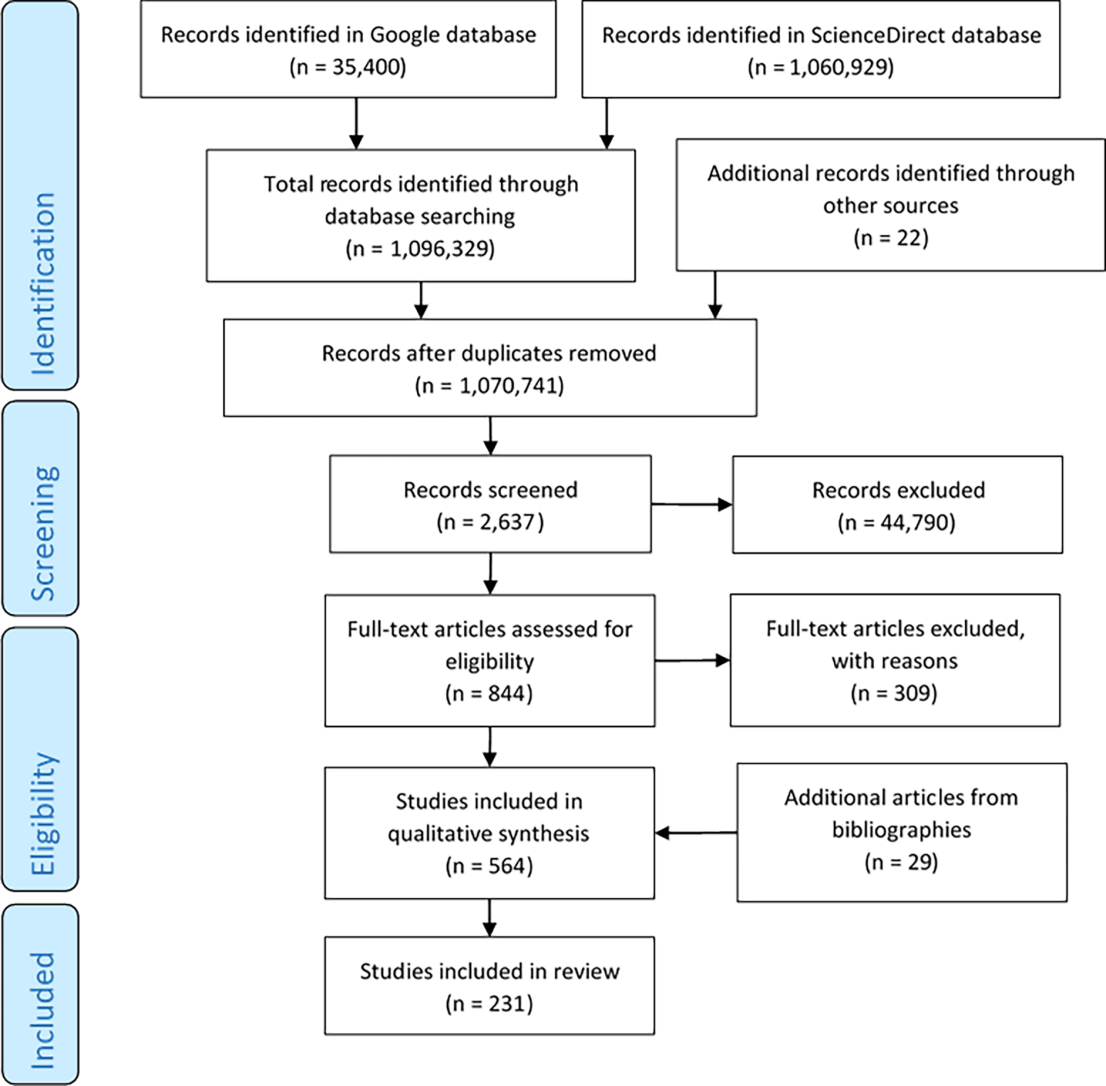
Search strategy and selection of the studies.

For the articles from *search 2* and *3*, the analysis was aimed to obtain: a deep understanding of the technologies and processes involved; data related to the design and fabrication of patient-specific bone implants; and to identify risk factors related to their design and fabrication. Additionally, the reference list from collected papers was systematically reviewed to identify further items. Once all applicable literature was identified (Fig 2) the tailored QbD approach adapted specifically for 3D printed bone implants was formulated.

With regards to the interviews’ data extraction, an interview guide was used to maintain the direction of the conversation and the relevant lines of enquiry, whilst probing into the issues of interest (see supplementary material in S2 File for further details on this procedure). To obtain a complete and accurate description of the interviewee’s responses and comments, all interviews were recorded and transcribed for further analysis. Moreover, at the beginning of each interview a consent form was delivered to each participant, explaining that the information that they will provide will be considered confidential and that only a de-identified summary of results may be used for presentations and publications. Consequently, each participant signed the consent form and approved the interview be audio recorded. The types of questions that this research addressed were *descriptive* and *structural*. *Descriptive* questions are asked to get descriptions of things and processes in order to get insights, or to check validity or accuracy about something [39]. *Structural* questions help the researcher to categorize groups of things and processes and to understand its relationships [39].

Qualitative data analysis required to examine, categorize, tabulate, test and combine evidence to address the initial propositions of a study [42]. The data analysis for the semi-structured interviews followed two key steps recommended by Eisenhardt [43]: within-case and cross-case analyses. In this study the within-case analysis was concerned with the evaluation of the collected data, as well as the reporting of the findings of each individual case study. A systematic reading through each transcript was performed, to then assign codes to tag segments with similar content to sort them into separate categories for a final distillation into major themes (see supplementary material in S2 File for further details on this procedure). The codes were pre-designed using the deductive coding technique based on the four groups of questions designed for the interviews [44]. It is worth mentioning that due to the open ended nature of the interview questions, the answers for some particular questions were mixed up with another question. As a result, part of the data gathered was of an “unstructured” nature consisting of long paragraphs which were organized in a structured and evidentiary-based manner to be able to draw conclusions as the study progresses. The information obtained from each interview provided an insight into how different factors of the adapted QbD system were perceived by experts from different fields of expertise. This was presented in the form of evidentiary tables, detailed in supplementary material in S2 File, containing the classified evidence from each interview using Miles, Huberman [45] tabular approach.

Following this, the cross-case analysis was performed to find patterns, agreements, and disagreements in opinions between the interviewees [46]. To facilitate the cross-case analysis, the information in the form of evidence extracts, was categorized in tabular manner based on the coding used (see supplementary material in S2 File). Once all the evidence had been organised, the results of this analysis were used to enhance and confirm the preliminary results obtained from the adaptation of the QbD approach, and to report participants’ opinions and concerns.

## Results

### Prior QbD study descriptive statistics

**Fig 3:**
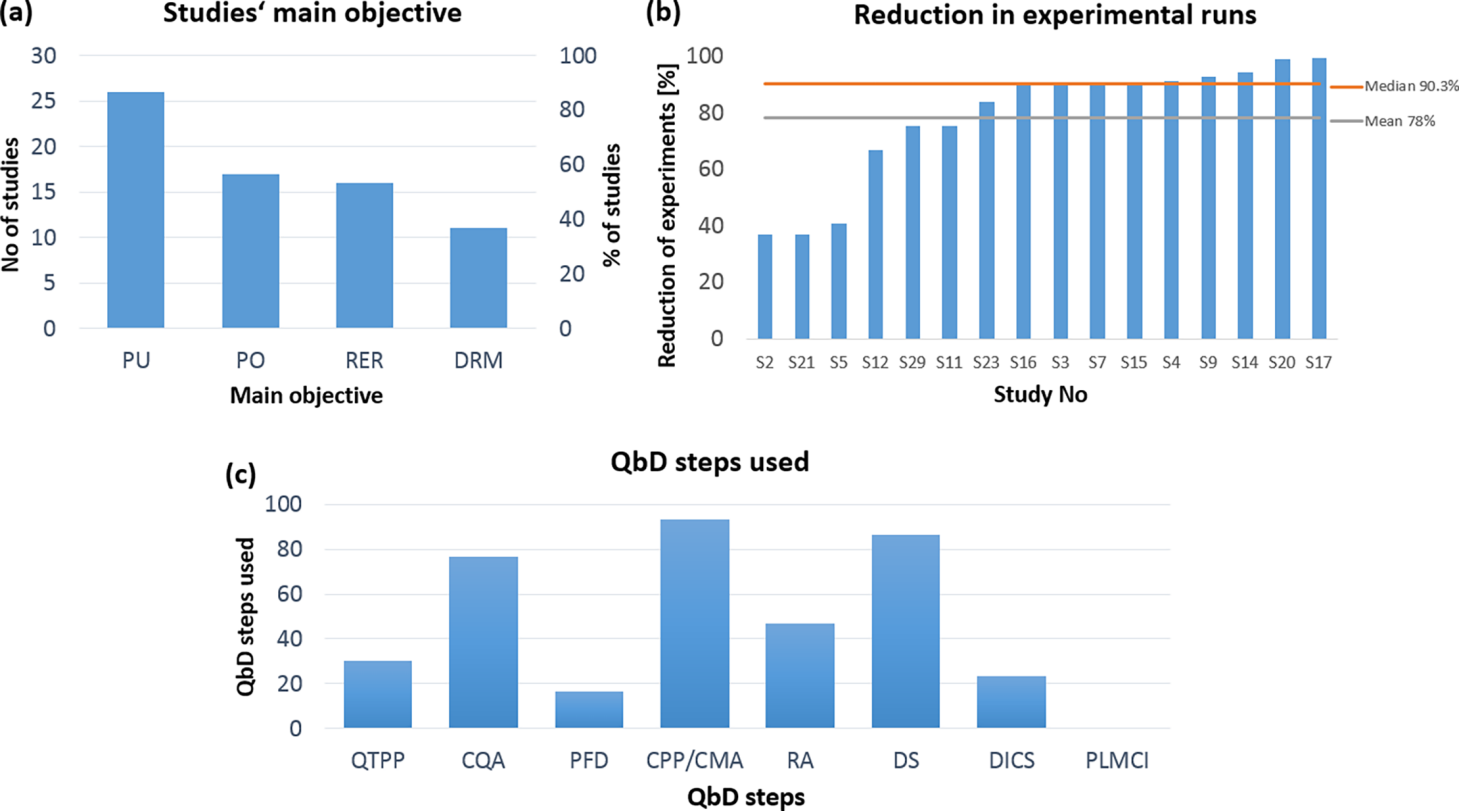
(a) Studies’ main objective, PU: Process understanding, PO: Prediction and optimization, RER: Reduction of experimental runs, DRM: development of robust manufacturing; (b) Percentage of reduction of experimental runs per paper; (c) QbD steps implemented in the reviewed studies.

A total of 30 prior completed QbD studies were statistically and qualitatively analysed to identify: the most implemented QbD steps; reasons to use the QbD system in research; positive results and drawbacks that were encountered in the QbD implementation; the total reduction of experiments obtained by using the QbD system. According to our results, QbD has been widely used in different pharmaceutical fields for several purposes, such as formulation and process design [27], improvement of drug manufacturing [47–49], and development of nano based pharmaceutical products [50–52], (See supplementary material in S1 File). Moreover, it was found that the most implemented QbD steps in the reviewed studies are: identification of critical process parameters and material attributes (CPP/CMA), design space (DS), and identification of critical quality attributes (CQA), with 93%, 87%, and 77% respectively (see Fig 3C). Whereas the QbD steps used the least were: development of strategies for product lifecycle management and continuous improvement (PLMCI), process flow diagram (PFD), and design and implementation of a control strategy (DICS), with 0%, 23%, and 17% respectively.

Furthermore, shown in Fig 3A it was identified that QbD was mainly used to achieve four different objectives: process understanding (PU), prediction and optimization (PO), reduction of experimental runs (RER), and development of robust manufacturing methods (DRM).

The majority of the studies (86%) used QbD to enhance process understanding (PU). The identification of CQA and CPP/CMA were the main steps of the QbD approach used for this purpose, representing 77% and 93% respectively (Fig 3C). For example, Verma, Lan (52) used QbD to gain a comprehensive understanding of the preparation and processing of nanosuspensions via microfluidization by identifying various two-way interactions between independent variables which were impossible to detect using other methods. Similarly, Zhang, Yan (47) used QbD to improve the manufacturing process with regard to the understanding of botanical drug products. In this study they identified the potentially critical factors on the performance of ethanol precipitation to later develop a potential design space of the ethanol precipitation operation. According to Zhang, Yan (47) “*QbD is a powerful tool in improving the knowledge of the relationships between potentially high-risk factors and the performance of ethanol precipitation…*”.

Prediction and optimization of pharmaceutical formulations (PO), and reduction of experimental runs (RER) were the second and third reasons for using QbD in the 30 studies, with 56% and 53% respectively (see Fig 3A). A total of 16 studies out of 30 aimed for the reduction of experimental runs. Incredibly, 50% of these studies reduced their experimental runs by more than 90%. On average the reduction of experimental runs was 78% (Fig 3B). In regards to optimization of pharmaceutical formulations, Mazumder, Pavurala [53] optimized the formulation of an oral disintegrating film to reduce its dissolution time from 10–40 mins to 3–6 min, representing an improvement of 85%. In another study, Schmidt and Molnár [54] reduced the purity testing of non-sedating antihistamine using an ultra-high performance liquid chromatographic (UHPLC) method from 160 min to 4 min, representing an impressive 40-fold increase in productivity.

The fourth reason identified for the implementation of QbD was the development of robust manufacturing methods (DRM) by reducing variability in production. Merely 36% of these studies used QbD for this purpose (Fig 3A), and 23% implemented the 7^th^ step of the QbD approach, which aims to design and implement a control strategy (DICS), as shown in Fig 3C. A good example of variability reduction with QbD is Cun, Jensen [51] study, where they were able to increase the encapsulation efficiency of Poly(DL-lactide-co-glycolide acid) PLGA nanoparticles from 2.01% to 60%, and reduce the variation efficiency from 49.17% to just 10%. Also Dubey, Boukouvala [55] were able to improve their production variability. In their study they reduced the tablet coating variability by more than 50%, and concluded: “*These results reported here demonstrate that the new coating process optimized by this QbD study was robust and produced consistent results*”.

In summary, the review of prior QbD studies suggests that the majority of them presented low levels of maturity in regards to the product development life cycle. The reason is that most of the research studies did not aim to reach commercial stages; therefore, the 7th (DICS), and 8th (PLMCI) QbD steps were perceived as being irrelevant for most of these research studies. Additionally, it has to be noted that the QbD system was developed to assist and accelerate the product development process in pharmaceutical companies, therefore it is not surprising that all the steps of the QbD system were not implemented in any study.

Nevertheless, it is evident that all of these studies showed positive results from the implementation of QbD. Moreover, despite that only a few studies aimed for DRM and implementation of the DICS step, they achieved remarkable results. Furthermore, most studies had a dramatic reduction of experimental runs and process variability, which can be translated to lower cost and improved quality. The implementation of QbD can provide similar benefits to different kinds of processes and products regardless of the industry to which they belong. Therefore, QbD is a powerful system that can assist in the development of 3D printed bone implants and scaffolds to provide better products and process understanding, ensure robust manufacturing, and minimize development risks and cost.

### QbD Step 1: Ideal quality target product profile

The Quality Target Product Profile (QTPP) is a description on how the product will be used by the end user, providing important information about its ideal characteristics and features in relation to safety and performance [56]. This will assist new product development phases, directing the development process to a clear goal [57]. It will also assist in identifying medical device development failures in the early development phases, and reduce the total development time and costs [57]. Therefore, before starting the development of a new product it is important to define the QTPP, drawing on the expertise of multiple professions, including scientists, engineers and medical specialists, to optimally meet future product expectations. Definition of the QTPP should also take into account users’ needs in relation to product sales and market success [58]. Moreover, it is important to point out that the formulation of QTPP is a dynamic process which receives feedback from all of the QbD systematic processes. As a result, QTPP is updated through the product development life cycle as new knowledge is acquired [58].

In order to describe the ideal quality characteristics of custom 3D printed bone implants, eight quality dimensions will be used to create the framework to measure product quality, giving consideration to various perspectives and market competitiveness [59].

Garvin (60) explains five approaches to define quality, based on different perspectives. These approaches are *the transcendent based approach*, *user approach*, *manufacturing based approach*, *value based approach*, and *product based approach*. In the case of bone implants, the client is usually the orthopaedic surgeon, who makes the final decision on the choice of prosthesis, but on rare occasions the patient may have the final decision. Consequently, there are three quality definitions which can inform our understanding of the market for medical devices. These definitions are the *user based approach*, the *manufacturing based approach* and the *product based approach*.

The *user based approach* defines quality from the clients’ perspective taking into account their wants and needs and how the client is satisfied with the product [60]. This approach in defining quality is supported by the orthopaedic surgeon’s decision making process, where the choice of one brand over another depends mostly on brand reliability, which is based on medical and scientific studies of prosthesis failure rates. The advantages of this approach is that it allows for a more accurate identification of product needs and definition, leading to better user experience, reduced complaints and improved functionality [61].

In contrast, the *manufacturing based approach* is focused on the reduction of scrap, rework and product failure [59]. This means that quality is focused on compliance with product specifications compliance from the engineering and production point of view [59]. This definition is supported by the strict product regulations which medical devices are subjected to in order to comply with medical requirements. Moreover, Sharkey, Sethuraman [62] found in a survey study that 97.1% of the 102 patients of total hip and knee replacements responded that the main determinant to choose an implant is the quality and not the cost. Moreover, it was found that a large percentage of patients (84%) are willing to pay additional costs for a better, but more expensive implant. This means that from the patients’ point of view, quality is based on cost perception, where a higher cost can be interpreted as better quality. This assumption supports the *product based approach* where a product with higher quality is considered more expensive [60].

In the case of custom 3D printed bone prosthesis and scaffolds, the clients are medical doctors experienced in regards to the product required, and are part of the design process. They interact directly with the designer, providing instructions and verifying if the implant fulfils their needs. Therefore, in any company dedicated to custom 3D printed prosthesis and scaffolds, the products and the service are equally important. Consequently, clear communication and direct interaction with medical doctors is essential in order to provide a suitable product. Moreover, direct cooperation and high levels of service between the manufacturing company and its clients is also crucial for this contemporary arrangement.

The eight dimensions of quality are basic elements to analyse product quality characteristics [60]. The importance of each dimension in a specific product depends on its market and customer needs. Thus, some dimensions are more critical for competitive success [63]. According to Garvin [63], *performance* is the first dimension of quality, and can be defined as the product’s functional attributes. The second quality dimension is *features*, and refers to those additional traits which complement the basic purpose of a product. *Reliability* is the third dimension and refers to the product’s failure rate. *Conformance* is the fourth dimension and relates to how a product fulfils design requirements and standards. The fifth quality dimension is *durability* and is a measure of a product’s life span that can be linked to technical and economic product aspects. *Serviceability* is the sixth dimension and is related to the external aspects of the product, such as the relationship and interaction between the company and the client, and also the speed and competence of product repair. *Aesthetics*, which is the seventh dimension, refers to how the product is perceived by the user’s senses and personal preferences, such as colour, taste, and smell. The last quality dimension is *perceived quality*, which shows how a product is perceived by the client, noting that not all clients are experts or have a clear understanding of the product’s attributes. Consequently consumers rely on product brand image, advertising and market trends. In Table 1, the eight quality dimensions related to custom 3D printed bone implants are described based on an ideal product.

**Table 1:** The ideal eight quality dimensions of 3D printed bone implants.

Quality approach	Dimension	Description
**Product-based approach**	Performance	Each 3D printed bone prosthesis and scaffold is custom designed to restore the functional characteristics of patient’s bone, provide long term osseointegration, operate properly in normal conditions, and to last the required number of years for its purpose.
	Features	3D printed bone prostheses have special features such as bioactive surfaces that promote bone ingrowth, a modulus of elasticity similar to the host bone, are specifically designed according to each customer’s geometry and functional characteristics, and possess a hierarchical macro, micro, and nano architecture that resembles bone structure.
	Reliability	Due to the fact that each prosthesis is custom made and fabricated with the latest technologies and materials, its life expectancy should match or exceed traditional bone prosthesis reliability and functional characteristics. In the case of bone scaffolds they are tuned to resorb based on each patient’s biological conditions to restore bone tissue. Moreover, The fabrication process of such products should be robust with minimum quality variations.
**Manufacturing-based approach**	Conformance	Each 3D printed bone prosthesis meets or exceeds medical device regulations. The design and fabrication processes are subjected to strict quality tests (control) to provide a product with zero defects (according to tolerances needed).
	Durability	Products are made with the best materials and technologies available, using a direct interaction with the customer to design custom bone implants that exceed the life expectancy of traditional bone prosthesis.
**User-based approach**	Serviceability	Each client is unique. Therefore, the company is responsible for providing a personalized service where each client is involved in the prosthesis design process. Moreover, the company must meet all customer specifications on time and be responsible for any irregularity. Additionally, it is important to provide an easy and accessible service to any potential customer.
	Aesthetics	The product has the correct materials and appearance for the target market. The technologies used for the product design and fabrication allow a precise reconstruction of defects from trauma or surgery, providing a correct custom shape and superficial finish to achieve better cosmetic enhancement and functional rehabilitation.
	Perceived quality	In this case the client has a clear understanding about the product’s attributes, which can be found in medical performance reports and statistical data.

### QbD Step 2: Critical quality attributes

#### Overview

Critical Quality Attributes (CQA) are the product characteristics which should be contained within certain limits to ensure that they conform to desired quality standards defined in the QTPP. The CQA can be chemical, mechanical, biological or microbiological [33]. For an effective identification of CQA, scientific and risk management rationale are used taking into account product knowledge, and business and regulatory requirements [29]. However, for an early product development phase, CQA are just identified but not defined within limits. Nevertheless, they may be updated as new knowledge is acquired, which is the case of this study.

Bone tissue engineering is the combination of biology and engineering to design functional engineered structures to repair bone tissue [23]. Thus, bone tissue engineering should help to provide a complete restoration of the damaged tissue, recovering its mechanical and biological properties and functions [64]. For the identification of CQA of custom 3D printed bone implants, it is important to take into account the hierarchical structure of bone and its properties, which are defined from molecular, nano, micro and macroscopic scales. Moreover, it is necessary to understand which performance indicators of bone implants can be used.

According to Giannoudis, Dinopoulos [65] there are three main performance indicators for bone substitutes (Table 2). The first performance indicator is osteoinductivity, which is a scaffold property where multipotential mesenchymal cells (MSCs) are stimulated or attracted to the material surface, to later differentiate into osteoblasts and form ectopic bone in vivo [65]. The second is osteoconductivity, which is the scaffold capability to allow new cell colonization, bone ingrowth and blood-vessels formation [66]. Osseointegration is the third indicator which is the bond between new bone and the scaffold biomaterial [65]. The identification of CQA of custom 3D printed bone implants was made based on different biological, physicochemical, mechanical, dimensional, and functional characteristics necessary to comply with the performance indicators mentioned above [67, 68].

**Table 2:** Performance indicators of bone substitutes.

Performance indicators	Definition
Osteoinductivity	Scaffold property where multipotential mesenchymal cells (MSCs) are stimulated or attracted to the material surface, to later differentiate into osteoblasts and form ectopic bone in vivo [65].
Osteoconductivity	Scaffold capability to allow new cell colonization, bone ingrowth and blood-vessels formation [66].
Osseointegration	Bond between new bone and the scaffold biomaterial [65].

#### Biological characteristics

The biological characteristics of bone implants and scaffolds are the most important, because they dictate how the body of the patient will react to it. The first reaction of the biological environment to any biomaterial is the conditioning of its surface by a rapid adsorption of proteins on it [69]. This occurs during the inflammatory phase, which is vital for the subsequent steps of the healing cascade in order to have a successful bone healing process [70]. Some of the proteins absorbed act as cell receptors that promote cell attachment biomineralization and matrix maturation due to their chemotactic or adhesive properties [71]. Furthermore, the injury site during healing is influenced by different factors such as hormones and nutrients, growth factors, oxygen tension, pH, the electrical environment, and mechanical stability [72]. Including, external factors such as bacterial infection which can drastically affect the healing process leading to prolonged antibiotic therapy or premature implant failure [73].

The ideal artificial bone should act as a scaffold (lattice structure) with a stable interface, and not produce toxic by-products which cause allergies and inflammatory reactions [74]. Moreover, the ideal bone scaffold should be a three dimensional resorbable biocompatible structure, that allows the flow of cell nutrients and waste, and stimulates cell migration and adhesion [75], thus promoting bone tissue formation and vascularization (Osteoconduction) [76].

At the macrostructure level, the biological performance of the lattice structure of scaffolds and metallic implants depends on their pore size, pore shape, pore interconnectivity, and percentage of porosity [77, 78]. These microstructural properties are the basis for cell proliferation and promote bone regeneration, rapid cell growth, vascularization, and interconnection between the implant and the local bone area [79]. Moreover, these properties directly influence the transport of nutrients and waste removal through the implant [67], allowing for the creation of hydrodynamic microenvironments which mimics natural conditions in vivo [77]. Thus, permanent and resorbable bone substitutes should act as a extracellular matrix for an adequate cell formation [79].

It has been demonstrated that at the nano scale level, surface properties also affect cell behaviour [80], accelerating the early stages of bone healing [81]. These are caused by physicochemical and biochemical reactions between the biomaterial surface and living cells, influencing intracellular signalling and affecting cell proliferation, differentiation, and adhesion [77, 82]. Surface properties involved in these processes are topography, roughness, and viscoelasticity [82]. Therefore, controlled material topography is needed in order to provide a high surface area for cell attachment and improve osseointegration and osteoinduction [82, 83]. However, in joint prostheses, some surfaces are preferred to be polished to improve its dynamic contact [84].

#### Physicochemical characteristics

Material biocompatibility is directly influenced by its surface physicochemical properties, which can modulate the cell environment, regulate cell adhesion and migration, and eventually promote bone healing [85, 86]. This is done by using materials and surface treatments that can release ions and molecules that can penetrate the cell membrane or activate its bound receptors [82]. Surface energy affects the wettability of a surface, which play a vital role in the initial stage of wound healing [87, 88], and is strongly correlated with cell adhesion and proliferation [89]. Material surface chemistry and topography also dictates material corrosion, wear resistance in permanent implants, and how long a degradable scaffold will last [86]. For example, most metallic implants are treated to change their surface chemistry so as improve its corrosion and wear resistance [90]. Alternately, resorbable scaffolds are chemically tuned to have a resorption rate similar to the local tissue healing process [74]. Additionally, implants and scaffolds, should be chemically strong enough to tolerate sterilization processes, and the corrosion induced by extracellular body fluids without any adverse reactions [91–93].

Currently in research another tissue engineering strategy to improve bone scaffolds and metallic implants is the incorporation of bioactives such as therapeutics and growth factors to stimulate cellular growth and cellular differentiation for healing, bone formation and vascularization, and to reduce bacterial infection [94–96]. Growth factors act on recruiting local and distant mesenchymal cells to differentiate into osteoblast and form bone tissue [66]. Some of the growth factors that can be used in bone scaffolds are vascular endothelial growth factors (VEGFs), platelet derived growth factor (PDGF), bone morphogenetic proteins (BMPs), and fibroblast growth factors (FGFs) [79, 97].

#### Mechanical characteristics

There are several mechanical properties that are needed for ideal bone scaffolds and prosthesis. For example, in resorbable scaffolds compression strength is important to provide structural integrity and support to the affected area during the healing process [98]. Whereas, for load bearing non-resorbable scaffolds fatigue resistance is needed to support in vivo cyclic stress [99]. Other mechanical properties that have to be taken into account are flexural modulus, Young’s modulus maximum strain, and tensile strength, hardness, and toughness [100, 101].

#### Dimensional characteristics

Scaffold dimensional accuracy at macro, micro and nano levels is vital to achieve some of the requirements mentioned previously, such as pore shape, pore size, surface topography, and mechanical integrity [100]. At the macro level, implants with custom-fit geometry in respect to the bone defect should be dimensionally accurate to match the anatomic deficit shape [102]. Thus, ideal bone scaffolds and prostheses should mimic the external shape of the patient’s anatomical defect and fracture sites. This results in better cosmetic results, structural support, joint performance improvement, surgery time reduction and faster recovery [103–105]. At the micro level (0.1 to 1mm) scaffold features, pore size and shape affect the scaffold mechanical properties and the response of cell multitudes [78, 102]. Furthermore, for features at the nano level, individual cell behaviour is affected [106, 107]. Therefore, the dimensional accuracy of scaffolds at all the hierarchical levels is critical in order to achieve its biological, mechanical, and anatomical characteristics [108].

#### Functional characteristics

Personalised bone implants with a patient-specific shape can ensure a precise reconstruction of defects from trauma and better mechanical stability. Nevertheless, there are more aspects to bone regeneration than just geometry [109]. Customised 3D printed bone implants and scaffolds should also be biologically active to restore the functional characteristics of patient’s bone such as vascularization for an appropriate diffusion of oxygen, nutrients and waste products [110]. Furthermore, it is critical to consider how the patient’s neuromusculoskeletical system might adapt to satisfy the new dynamic moment requirements at each joint during everyday activities [111]. For example, a change in bony geometry at the proximal femur will impact muscle tendon pathways and muscle moment arms at the hip and may also alter the ideal force-length operating range of the muscles during such activities. Moreover, the implant’s material should resemble the mechanical characteristics of native surrounding tissues [109], e.g. resemble the patient’s bone modulus of elasticity to avoid stress shielding and to promote early bone regeneration [94]. In the case of metallic implants that are in contact with surrounding soft tissues, such as muscle and mucosa, shear forces lead to thicker fibrous capsule and inflammation, therefore, these forces should be avoided or reduced using smooth polished implant surfaces [78]. Finally, if the implant aims to restore bone biological performance, it is important to take into account that these kind of implants will require customised rehabilitation programs to provide the best conditions for bone maturing and healing [112]. These characteristics act as a fourth extra dimension apart from the 3 dimensions included within the patient’s bone geometry allowing proper physiologic and mechanical functioning.

A summary of the ideal CQA of custom 3D printed bone implants and scaffolds is presented in Table 3.

**Table 3:** Summary of the ideal CQA of custom 3D printed bone implants and scaffolds.

Dimensional	Mechanical	Biological	Physicochemical	Functional
Match patient specific geometry	Low modulus of elasticity (resemble bone Young’s modulus)	Material purity	Biocompatibility	Patient’s bone characteristics
Macro surface geometrical accuracy	Shear/Compressive/tensile strength	Protein adhesion	Tuned resorption rate (biodegradable implants)	Muscle moment arms
Micro geometrical accuracy	Fatigue strength	Cell adhesion	Corrosion resistance (permanent implants)	Real life implant’s loading conditions
Nano geometrical accuracy	Hardness	Cell migration	Surface micro and nano topography	Implant’s bone interface contact forces
3 dimensional structure	Toughness	Cell proliferation	High surface area	Implant’s soft tissue interface contact forces (metallic implants)
Pore size	Poisson's ratio	Mineralization	Bioactives	Customised rehabilitation program
Pore shape	Wear resistance	Control of inflammation	Resistance to sterilization process	
High pore interconnection		Antibacterial properties	Surface energy (wettability)	
High percentage of porosity		Transport of nutrients and waste removal		

### QbD Step 3: Process flow diagram of 3d printed bone implants

Process mapping helps to represent processes visually through the identification of all the necessary steps, participants and decisions in a process. This improves process understanding, and helps to identify problem areas and improvement opportunities [113]. In the QbD approach, process mapping offers the opportunity to identify the design and manufacturing process parameters that can potentially affect product performance and CQA. This helps to achieve a more accurate identification of the most critical processes, to later design the most appropriate monitoring and controlling methods to ensure the desired quality is obtained [33].

**Fig 4:**
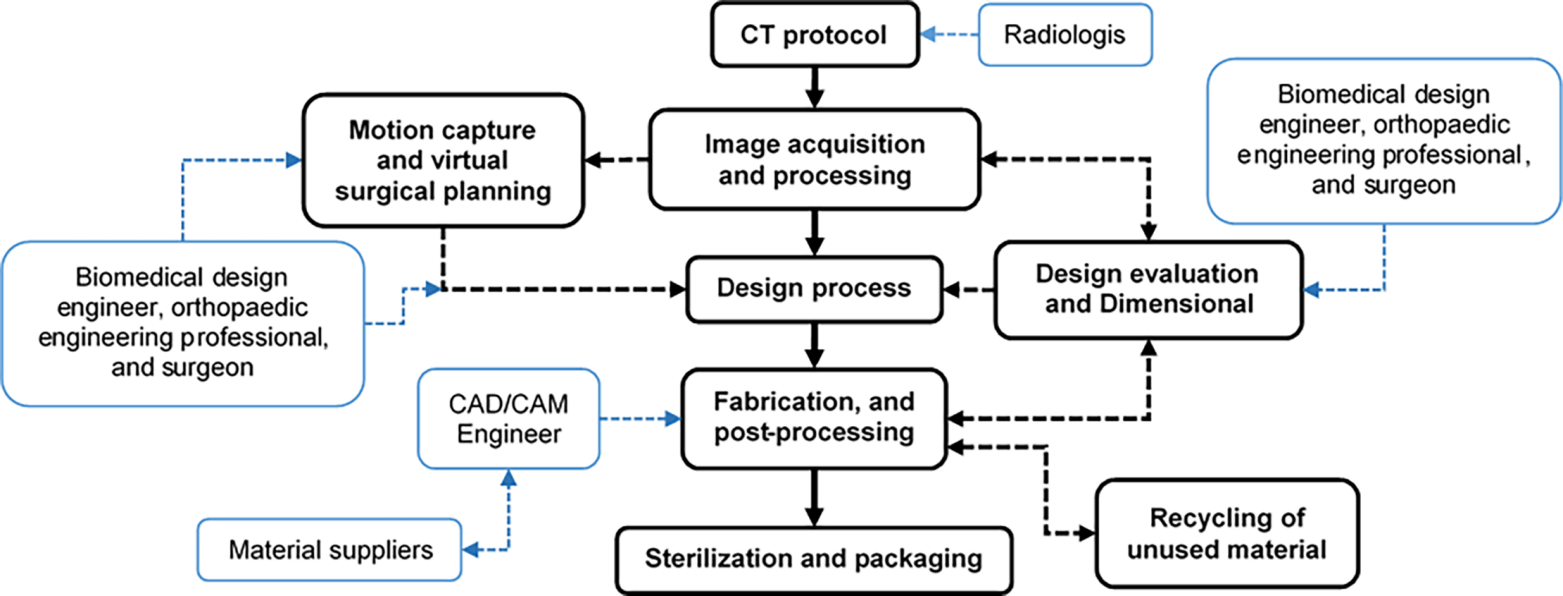
General process flow diagram of custom 3D printed bone prostheses and scaffolds.

In general, the process to produce custom 3D printed bone implants and medical devices can be summarized in eight main processes (Fig 4): (1) CT protocol; (2) patient’s image acquisition and processing; (3) implant design; (4) design evaluation and dimensional validation; (5) motion capture and virtual surgical planning; (6) fabrication; (7) recycling of unused material; and (8) sterilization and packaging [114]. However, a common view of the process is not detailed enough to encompass all the different activities and parameters that can affect the CQA of such products [27]. Taking into consideration that the purpose of this study was to have a clear understanding of the different parameters that can affect the CQA of custom 3D printed bone implants, an exploratory analysis of different workflow approaches was necessary. Through a literature review, different design and fabrication approaches were identified [8, 114–132] and critically evaluated to provide a more detailed picture of the activities involved in each process.

**Fig 5:**
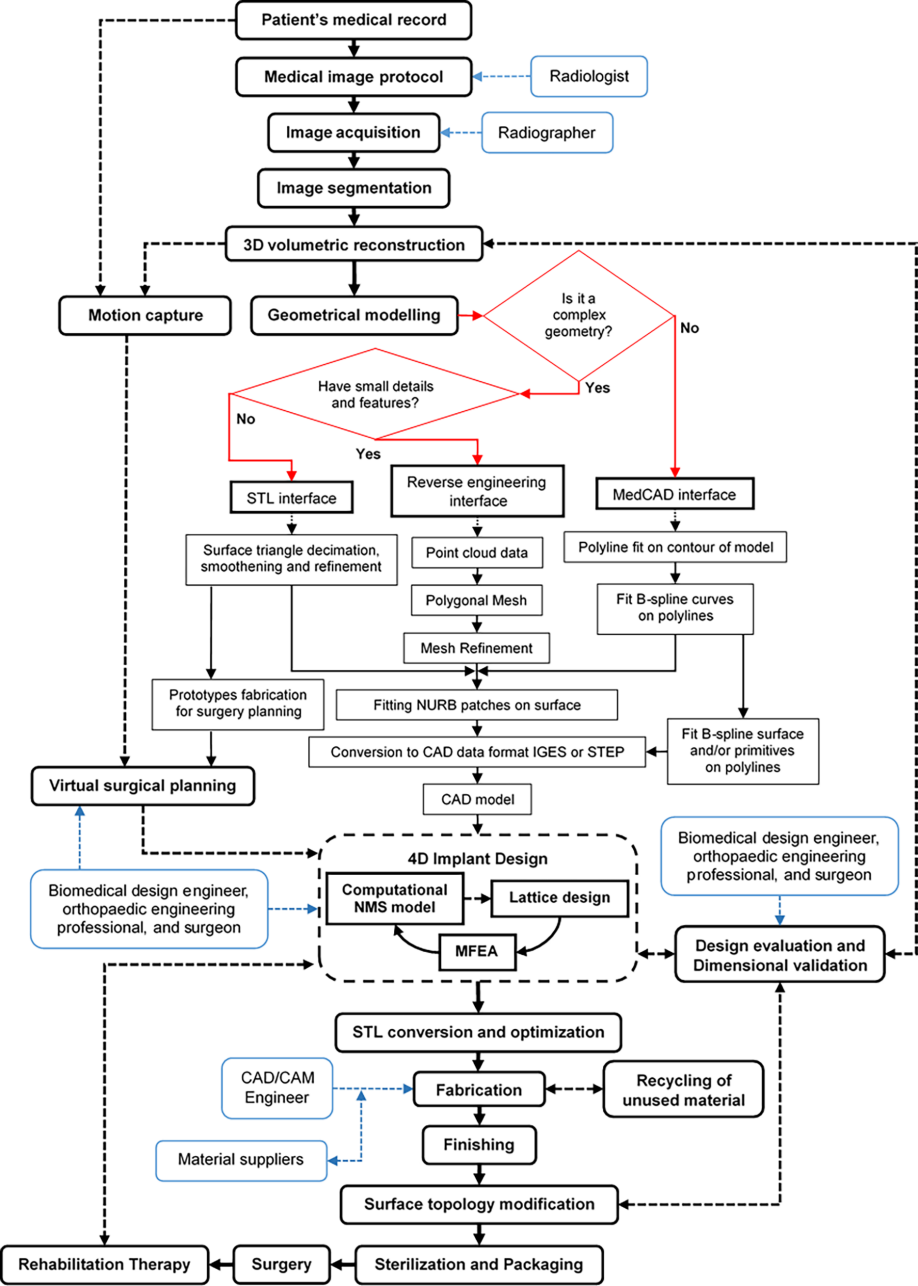
Detailed process flow diagram for the design and fabrication of custom 3D printed bone prostheses and scaffolds.

Moreover, it was identified that there are two process activities (*Geometrical modelling* and *Implant design*) can be carried out utilising different routes depending on the implant desired outcomes, complexity and chosen characteristics. The result of this analysis was a more detailed outline process map (Fig 5) that will aid future researchers and practitioners to design the most appropriate workflow approach for a specific medical device. In the detailed process map the main processes are represented by black round boxes, their different routes and their activities are represented by black rectangular boxes, and the different experts that interact in each process are represented by blue round boxes. The explanation of each main activity is provided in the following paragraphs.

#### Image acquisition and CT protocol

Image acquisition is one of the vital processes for the development of custom 3D printed bone implants, because it essentially informs how the implant design process is obtained. Today, the common medical image techniques to assist AM are Magnetic Resonance Imaging (MRI) and Computed Tomography (CT) [133]. From the image data point of view the difference between CT and MRI technologies is that CT provides images of bone tissue with better resolution, while MRI technology provides high resolution images of soft tissues [134–136], making this technology suitable for the development of 3D printed organs. Another system is the micro CT that is capable of taking images of nano size cross sections giving 10,000 times more resolution than conventional CT scanners and at a lower level of radiation exposure [137]; and it can be used for the development of high precision implants [12].

The advantage of MRI technology is that there are no harmful effects on the human body; however, it has to be carefully used in patients with implants due to the strong magnetic field used with this equipment [134], and is contraindicated in patients with claustrophobia [137]. Whereas, CT exposes the patient to a dose of radiation that can be potentially harmful, it has to be strictly controlled by the medical doctor and radiologist according to standardized procedures [138]. However, the most used medical image technology for AM is the multislice CT (MSCT) scanner [139], because its image post-processing is easier [133].

The image acquisition process starts with the scan of the patient’s region of interest, based on the necessary protocol [140]. In the case of the CT scanner, settings are based on image slice thickness, slice spacing, number of pixels, and grey scale [141]. Grey scale is measured in Hounsfield Units (HU), which represents tissue density for an easy identification of different tissues in CT images. For example, in CT images, bones appear in a white colour, soft tissues in grey, and air cavities in black [142].

#### Image segmentation

After acquiring the patient’s medical image, the file is saved in DICOM format (Digital Imaging and Communication in Medicine) which is the standard format for CT images within the medical field [141]. At this point, the image data of the scanned area is a group of 2D images that need to be segmented in order to separate the bone tissue from objects that are not relevant such as muscle and fat [143]. The image segmentation process can be performed manually, automatically or using both algorithms and filters [119, 144]. There are several segmentation methods and algorithms for medical images, such as edge detection, region growing [144], threshold based, clustering based, and deformable models based techniques [145]. Nevertheless, the most common method is thresholding, where a threshold value is chosen using the radiodensity of different tissues to select a range of pixels with certain intensity (measured in Hounsfield Units) to highlight and separate specific tissues in the image, making for easier identification of organs and bones [144]. For the image segmentation process there are several software programs available that can be used with DICOM datasets, such as InVesalius (open source software) [143], Osirix (Pixmeo, Geneva, Switzerland) (open source software for Mac) [140] and Mimics Innovation Suite (Materialise NV, Belgium) (commercial software) which is the most common software used [5]. It important to note that careful segmentation is crucial as it defines the accuracy of the final model, and as a result is one of the most labour intensive steps requiring the most manual intervention [146].

#### 3D volumetric reconstruction

The 3D volumetric reconstruction is carried out after finalising the image segmentation process to generate a 3D image representation that can later be used to create the 3D CAD model for the implant design. The 3D volumetric reconstruction entails obtaining a three dimensional representation of the region of interest, segmented from CT images. Each CT image represents a cross section cut of the scanned region of interest with a specific thickness [147]. By stacking the images together, a three dimensional approximation of this region is obtained [147]. Through this process, pixels with the same intensity and same position in each CT image are converted into a pixel volumetric representation called voxel, and thus create a three dimensional image, which is later used to create a 3D model [147].

#### Geometrical modelling

The geometrical modelling process is conducted to generate a 3D CAD model from medical imaging data that can later be used as a reference to design the implant or scaffold by isolating the patients’ region of interest using the mirroring technique and Boolean operations [125]. There are three different traditional and modern geometrical modelling routes, which are used depending on implant complexity and the available software resources, for the design of personalized implants [125].

The first route is the *reverse engineering interface* or *NURB based method*, which is a traditional route for geometrical modelling. This method transforms the 3D voxel data from the volumetric reconstruction into a point cloud, that later is used in a reverse engineering software to create a polygonal mesh, which then is softened using NURB curves [115]. This process is based on four steps: *Pre-Processing* where the point cloud is optimized to reduce file noise and errors; generation of the *Polygonal Mesh*, where a faceted model is created from the triangulation of the point cloud; *Mesh Refinement*, where the mesh is refined; and errors are then corrected fitting *NURBS patches* [148]. To optimise the mesh resolution software, packages such as MeshLab (CNR, Pisa), and PolyMLib (Solid Modelling Solutions, Aichi, Japan) can be used [149]. After this, the optimised mesh can be converted into a STL file to be fabricated by additive manufacturing for preoperative surgery planning models or can be converted into a NURB surface to be used as an input for CAD software for implant design [148]. The advantages of this method are total control of implant thickness, smooth curves [125], clean 3D model representation, high data quality, stable configuration, less error in format transfer, and it can be used for complex shapes [115]. Nevertheless, the disadvantages of this method are that this process is tedious and time consuming [115, 125], and generates waved surfaces with an enormous number of control points, making its manipulation in CAD/CAM software very complicated [125].

The second route was developed to address some of the disadvantages of the NURB based method [125], such as data conversion issues, and served as a bridge between medical image and CAD software [115]. This method is based on the AIT MedCad program that evolved to *MedCAD interface* as a standard module for a medical imaging process software [115]. However, the drawback of *the MedCAD interface*, is that it is limited in terms of design, and cannot capture complex and detailed geometries [115].

The third route is the *STL design interface*, which is currently the most used method to avoid the tedious process of the *reverse engineering interface*. This is done converting the 3D volumetric model (voxel model) to a STL file, which represents the model with a triangulated mesh [150], to later import it into reverse engineering software for mesh refinement and error correction [115]. For the simplification of the implant design process, the region of interest can be isolated to reduce computing time and file size. This is done by eliminating the structures or surfaces that are not relevant for the implant design, with the remote assistance of the surgeon using viewer software such as SolidView [132].

The advantage of this method is that it uses a STL triangulated surface as an input instead of the point cloud [115], making it fast and efficient [118]. During this process the STL can be optimised to reduce the number of elements and correct errors, using software such as Magics (Materialise) [132]. Nevertheless, the disadvantages of *STL design interface* are: it requires triangulated surfaces without errors; is not suitable for geometries with small details and complex features [115]; and is limited by the many drawbacks of the STL format [151]. However despite all the disadvantages, this method is preferred by researchers due to its simplicity and efficiency [114, 116, 118, 142].

#### Virtual surgery and planning

Virtual surgery planning is an online collaboration between the surgeon, the orthopaedic engineering professional and the biomedical design engineer. The main focus of this virtual planning is to study and manipulate the patient’s bony defect to find the optimal solution to restore it to its normal condition. The process begins with an evaluation of the patient’s health history, particularly of the mechanism of injury and details of any distorted or destroyed anatomy [152]. The surgeon then decides the most suitable surgical technique and explains the specifics of the planned procedure to the engineers to later discuss the design of the fixation method and implant solution. This vital process requires a close collaboration between surgeon and engineers in order to understand the limitations and constraints of the surgery [152]. Once this process is completed the implant design process begins. It has to be mentioned that the surgeon’s active engagement in the virtual planning will improve technical performance, give confidence, and pay dividends on the day of the actual procedure [152].

#### 4D implant design

Current design methodologies and mass production of bone implants employs minimum variation of product characteristics in order to cope with the limitations of traditional manufacturing methods, which are based on statistical quality control processes with quality control activities, such as destructive testing and lot sampling. Moreover, in vitro testing is limited in testing real life conditions, and is also time-consuming and requires manually intensive measurements. These approaches are limited for the 3D printing manufacturing environment of medical devices where the manufacturing process is composed by variable and complex product characteristics, low production lots and high design requirements [153]. Additionally, current bone implant designs do not take into account joint contact forces that occur during real life activities [154]. To address these limitations, the next generation of bone implants should not merely comply with the unique 3D geometrical features of patients’ bone; instead they should include a 4 dimensional design where the physiological condition, and current health of each patient are also taken into account. Therefore, 4D implant design incorporates multiscale and multi-physics in order to address clinical problems that manifest at different hierarchical levels, such as at the whole organ, meso, micro and cellular levels [111]. For this, the design and performance of custom 3D printed bone implants should be performed and validated concurrently not just with mechanical and in vivo testing, but also with patient specific computational neuromusculoskeletal (NMS) predictions and multiscale finite element analysis (MFEA) to accelerate their development and fabrication process, and simultaneously improve their reliability before they are subject to clinical evaluation and fabrication [155–157].

Computational NMS models provide a non-invasive method to estimate in vivo contact loads of the patient’s region of interest accounting for muscle force contributions, which are influenced by external loading conditions, joint kinematics, as well as an individual’s task-specific muscle activation patterns, during real life activities [154, 158]. Moreover, NMS models help researchers and medical practitioners to understand the mechanisms of injury and disease of the musculoskeletal system and structural form-function relationships to better design, test, and validate bone implants [157]. MFEA enables virtual tests and simulations for more insightful study, design and optimization of micro and nanostructured hierarchical materials. These simulate the interactions of different implants microstructures and surfaces, with local cancellous and cortical osseointegration [159, 160]. Furthermore, MFEA helps designers to virtually check the structural, mechanical, and biological performance of implants, detecting weak areas in the design in order to strengthen them to support *in vivo* cyclic stress, before commencing production [99, 119, 146, 161].

#### Implant’s lattice design

At this point of the workflow process the designer has enough data to start to design the external geometry of the implant using the mirrored geometry of the contralateral anatomical site (for patients who are only unilaterally affected) or a scale idealized statistical shape bone model to create a 3D model that can be used as a reference to design the implant. At this stage it is also possible to simulate and plan surgical operations of bone replacement to reduce surgery time and errors during difficult surgeries [12, 162]. Additionally, bone cutting guides can be designed to improve surgery accuracy [117].

To obtain biomimetic bone implants such as porous scaffolds and prosthesis, they should mimic geometrical, biological and mechanical properties of bone with controlled macro micro and nanoarchitecture to facilitate cell proliferation, nutrients transport, and waste flow [98]. However, due to bone tissue complexity, most of these design approaches are simplified models that attempt to resemble bone tissue architecture and mechanical properties [163]. This is done using 3D unit cells that can be intersected with a patient’s bone 3D model from medical imaging data, using Boolean operations, in order to create volumetric lattice structures [98]. These design approaches can be classified into four main groups: *CAD based methods*, *implicit surfaces*, *image-based topology design*, *and topology optimization* [163, 164].

CAD based methods for bone tissue combine a biomimetic design with Computer-Aided Tissue Engineering (CATE) to create load bearing tissue scaffolds. This, provides the capacity to mimic bone porous macro structure and mechanical properties, using libraries of different unit cells based on platonic solids, archimedean solids, prisms and anti-prisms, and archimedean duals [115, 165–168]. The advantage of this approach it is that it can tailor the stiffness variation of the scaffold more effectively to mimic bone microstructure [166]. However, despite the advantages of unit cells CAD approaches, they are restricted by the availability of polyhedra shapes, and by its inaccurate description of complex natural shapes due to their straight edges and sharp turns [169]. Moreover, this method is time consuming [170], its parameterization is difficult, and requires high performance computers to reduce computational time and improve its manipulation and visualization [76, 167, 171].

Implicit surface modelling is a design method that has been introduced to overcome the disadvantages of polyhedral unit cells used in CAD based methods. The design of implicit surfaces comes from single trigonometric functions to generate cellular structures based on the triply periodic minimal surfaces (TPMS) [172], which are present in nature and describe material architectures such as crustacean skeletons, beetle shells, and butterfly wings scales [163]. The great advantages TPMS are that they allow the creation of biomorphic scaffolds with free and easy control of pore architecture [164]; can be repeated infinitely in three independent directions [173] with variable porosity [174] that can mimic mechanically cancellous and cortical bone [175]; and a simple computer program can perform the whole process automatically [172]. Furthermore, TPMS creates intricate labyrinths with smooth joints that reduces stress concentrations [175], and provide high surface-to-area ratios that improve cell proliferation [176].

Image based topology design is used to create optimized porous structures with voxel datasets using density distributions [177]. This method can create regular and random scaffold internal architectures based on voxel data [20, 122, 177]. The advantage of image based design is that it is a fast method that allows the creation of bone scaffolds with controlled internal architecture at different scales [163]. Moreover, this method, combined with topological optimization, allows control of the material used and the creation of optimal structures with less resources [20].

Topology optimization is an engineering design technique that has been applied in industries, such as automotive and aeronautics, to design parts that satisfy constraints such as performance, costs, and weight [178]. For the design of cellular materials with orthotropic properties such cancellous bone, topology optimization has been researched to obtain specified orthotropic mechanical properties of periodic microstructures under given constraints [164, 179]. Topology optimization methods can be classified as being either concurrent or non-concurrent. This depends on whether the method is applied to the macro structure or the unit cell (concurrent approach), or both (non-concurrent approach) [180]. In the case of bone scaffolds, topology optimization can be applied to improve the properties of their internal architecture by optimizing their unit cells in order to achieve a modulus of elasticity closer to that of a particular bone and/or to improve their properties for the purpose of transporting nutrients and waste.

#### STL file conversion and optimization

Before starting the fabrication process, the design should be checked again by surgeons and engineers to compare the design geometry with the patient’s CT images. After this, the CAD file has to be exported to STL format, which is the format that most 3D printers use [181]. Then, the 3D STL model has to be checked for potential errors caused during the file conversion. For this task there are dedicated software programs which automatically repair and edit STL files for 3D printing manufacturing [182], such as Netfabb by FIT, Viscam by Marcam, and Magics RP by Materialise [183].

#### Fabrication

For this step the first activity, is to set up the 3D printer machine parameters. These parameters vary depending on the type of system used, and the selected material. For example, the building layer thickness is an important parameter for all the AM systems because it is the one that controls the building resolution [183]. However, only for AM systems that use laser, such as selective laser sintering (SLS) and stereolithography (SLA), parameters such as laser power and beam diameter have to be accordingly set up depending on the material used [184]. Other parameters such as powder bed temperature, chamber and bed temperature, cooling cycle, scan speed are important in AM systems such as SLS and electron beam melting [184–186]. When the implant is manufactured and its temperature decreases to room temperature, it can be removed and properly cleaned.

#### Cleaning and finishing

Once the fabrication process is finished, the parts have to be removed to be cleaned from residual material and then further post processing steps can be done, such as sandblasting, polishing, homogenisation, and thermal treatments [183]. The removing and cleaning methods depend on the AM system and material used. For example, AM technologies, such as fuse deposition modelling (FDM) and stereolithography (SLA), fabricate parts with supports to brace overhanging features, and these supports have to be removed by hand after the fabrication process [7, 187]. In the case of AM that uses material in powder form, the residual powder has to be properly cleaned from cavities and porous surfaces [166].

#### Material recycling

Most AM systems have the advantage that part of the unbound material can be recycled after the fabrication process [183]. For example, in SLA, the remaining resin can be poured off and used for the next project [3]. In the case where the material is in powder form, the remaining particles can be sieved and combined with virgin material for its reuse [188]. However, material recycling can only be done a certain number of times because the mechanical properties of the material will deteriorate after each cycle [189]. Moreover, particle size and shape of the powder materials have to follow strict specifications to guarantee high quality parts and consistent mechanical properties [190].

#### Surface topography modification

Implant surface modification treatments are performed to improve biological, physicochemical and mechanical properties [191], such as bioactivity, biocompatibility, blood compatibility, reduce wear and increase corrosion resistance [95]. Surface modifications of metallic implants are needed to provide a better quality of bone tissue-to-surface contact, controlling tissue formation at the early stages of cell formation [82]. For example, some titanium surface modifications can improve osseointegration and osteoinduction [82], and it is proven that surface roughness in titanium alloys can accelerate the early stages of bone healing [81]. However, there are areas in implants that are preferred to be polished to improve dynamic contact [84], and soft tissue-to-surface contact [78].

There is experimental evidence indicating that some of the important factors of cellular recognition in biomimetic materials is nanoscale topography [192]. For example, some of the most innovative surface modifications in titanium alloys are TiO2 nanotubes, which are produced with electrochemical anodization and are a novel method to coat titanium implants surfaces [106]. The advantage of this approach is that by applying different voltages in different electrolytes containing fluorites [106], the size of TiO2 nano-structures, such as nanotubes, pillar-like nanostructures, and nano-dots, can be controlled [95]. Moreover, the adhesion of hydroxyapatite on TiO2 nano tubes surfaces is more efficient in promoting the growth of osteoblast than non-anodized surfaces [193]. Additionally, it is proven that TiO2 nano tubes accelerate adhesion/propagation of osteoblast cells by almost 400% [194].

#### Sterilization

Any implant has to be sterilized before its clinical use [139] to ensure the safety of the final product. This is to ensure a total elimination of all forms of microbial life, bacteria, viruses and spores [195]. There are several sterilization methods that can be used depending on the implant material, structure, and use [196]. Some of the traditional sterilization techniques used by the medical device industry are dry heat, ionizing radiation, steam, and ethylene oxide [197]. However, sterilization of patient specific implants with internal microstructures such as bone scaffolds can represent a challenge in selecting the most appropriate sterilization method [198], and getting the validation of Sterility Assurance Level (SAL), which is required for FDA approval [196]. Additionally, it has to be pointed out that depending on the material used in the implant fabrication, the sterilization process can affect its dimensions, therefore this has to be taken into account to maintain implant precision [114].

### QbD Step 4: Identify critical process parameters and critical material attributes

Through the identification of the CQA, it is clear that for the fabrication of custom bone prostheses and scaffolds, accurate design and fabrication techniques are needed in order to maintain a precise control of their dimensional, mechanical, biological, functional, and physicochemical properties [77]. The identification of critical process parameters (CPP) and critical material attributes (CMA) help to determine process parameters and material attributes whose variability can potentially affect CQA [33]. Therefore, CPP and CMA should be monitored and controlled to ensure process consistency, repeatability, and accuracy. However, at this early stage of product development, the specific AM technique and product material are not yet selected.

Nevertheless, AM technologies have a variety of different materials that are suitable for bone scaffold and prostheses. Depending on the fabrication technique, these materials are used in solid, liquid and powder form. Moreover, some AM technologies have the advantage that the unbound material can be recycled until a certain point without greatly affect the mechanical properties of the build part [199]. However, it has to be taken into account that the material recycling process can lead to material contamination and difficulties with material traceability [196]. Moreover, these material attributes have to comply with general regulations for biomaterials used for permanent implants. These regulations includes ISO-10993 and FDA standard tests, as well as quality control documentation to certify material characteristics such as biocompatibility, chemical composition, mechanical properties, purity, traceability, and storage [196]. An example of biocompatibility evaluation is shown in Table 4.

**Table 4:** Example of FDA recommended initial ISO 10993 biocompatibility evaluation endpoints for medical devices in contact with bone tissue and blood. Adapted from [200].

Device categorization		Biological effect												
Nature of body contact	Contact duration A–limited (≤24 h) B- prolonged (>24 to 30 d) C- permanent (>30 d)	Cytotoxicity	Sensitization	Irrigation or intracutaneous reactivity	Acute systemic toxicity	Material-Mediated Pyrogenicity	Subacute/Subchronic Toxicity	Genotoxicity	Implantation	Hemocompatibility	Chronic Toxicity	Carcinogenicity	Reproductive/Developmental Toxicity	Degradation@
**Tissue / bone**	A	X	X	X	O	O								
	B	X	X	X	X	O	X	X	X					
	C	X	X	X	X	O	X	X	X		O	O		
**Blood**	A	X	X	X	X	O		O	X	X				
	B	X	X	X	X	O	X	X	X	X				
	C	X	X	X	X	O	X	X	X	X	O	O		

X = ISO 10993–1:2009 recommended endpoints for consideration*

O = Additional FDA recommended endpoints for consideration*

Note * All X’s and O’s should be addressed in the biological safety evaluation, either through the use of existing data, additional endpoint-specific testing, or a rationale for why the endpoint does not require additional assessment.

Note @ Degradation information should be provided for any devices, device components, or materials remaining in contact with tissue that are intended to degrade.

For an easier identification and classification of CPP and CMA, scientific and quality risk rationale were used. This was performed by linking the four CQA groups previously identified (biological, mechanical, chemical, dimensional) to each of the processes of the workflow map, which can potentially affect them, as shown in Table 5. In the same way the CMA were linked to the CQA that can be potentially affected, as shown in Table 6. This procedure helps to identify risks more easily in the quality assessment process.

**Table 5:** Linking CPP activities with CQA groups.

Critical process parameters	Affected CQA
Patient’s Medical Record	Functional
CT Protocol	Dimensional
Image Acquisition	Dimensional
Image Segmentation	Dimensional, Functional
3D Volumetric Reconstruction	Dimensional, Functional
Geometrical Modelling	Dimensional, Functional
Computational NMS model	Dimensional, Mechanical, Biological, Functional
Lattice design process	Dimensional, Mechanical, Biological
MFEA	Mechanical, Functional
STL conversion	Dimensional
Fabrication	Dimensional, Mechanical, Biological
Material recycling	Dimensional, Mechanical, Biological
Cleaning and Finishing	Dimensional, Mechanical, Biological, Physicochemical
Surface modification	Dimensional, Mechanical, Biological, Physicochemical
Sterilization and packaging	Dimensional, Mechanical, Biological, Physicochemical

**Table 6:** Linking CMA with CQA groups.

Critical material attributes	Affected CQA
Recycling	Dimensional, Mechanical, Biological
Mix	Mechanical, Biological
Composition	Mechanical, Biological, Physicochemical
Storing	Mechanical, Biological, Physicochemical
Traceability	Mechanical, Biological, Physicochemical
Sterilizing method	Mechanical, Biological, Physicochemical
Mechanical	Mechanical, Biological
Biological	Mechanical
Physicochemical	Mechanical, Biological, Physicochemical
Material form	Mechanical
Necessary energy for binding	Mechanical, Biological, Physicochemical

### QbD Step 5: Quality risk assessment

#### Overview

Quality risk management (QRM) is an important element of QbD in order to verify that any changes in the product design are comprehended and correctly managed to ensure patient safety [201]. QRM ensures high quality product, identifying and controlling potential quality risks during development and manufacturing, using a realistic evaluation of the true level of risks that can occur [202]. One of the main activities of QRM is the risk assessment, where the initial list of potential causes of risk that can affect CQAs can be reduced by giving priority to only the most significant risks. Thus, these risks can be controlled through the product development process and its life cycle [203].

For a successful systematic implementation of any risk assessment, there are several QRM tools that need be used [204]. In the case of this study, the selected tools were the Ishikawa diagram and the Risk Breakdown Structure (RBS). The Ishikawa diagram, also known as fish bone diagram, is used to categorise risks in meaningful groups to facilitate the risk analysis process [205]. The Ishikawa diagram is composed of several branches, which are used to find and categorise the major influencing factors for a given problem [206]. The RBS is a table that depicts a hierarchical structure with descending levels that represent an increasing detail of risk sources to organise and structure risks in order to facilitate their understanding, communication and management [207].

**Fig 6:**
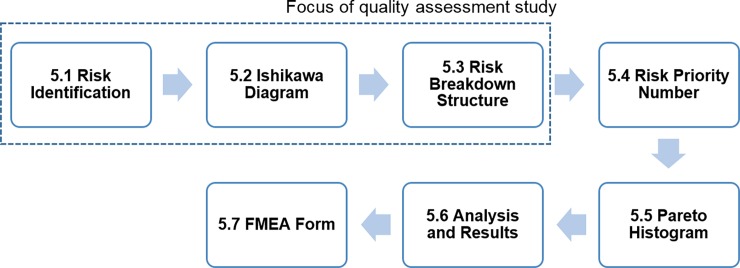
Flow chart of selected quality risk assessment tools, and scope of present study.

Fig 6 illustrates the focus of this present quality assessment study and the tools selected, which involves seven key elements. In future research the authors will work on a dedicated paper that will be focused on completing the four remaining QbD steps, to then develop a Failure Mode and Affects Analysis (FMEA) form containing corrective and preventive actions to mitigate all the critical potential failures and risks to prevent defective products reach the customer [208, 209].

#### Risk identification

Before risks can be properly managed, they need to be identified. Any factor with an uncertain probability of occurring that can influence the outcome of a project is considered a risk source or risk hazard. Identifying hazards involves learning as much as possible about what things could affect the project or could go wrong, and what the outcome for each would be. The risk identification process was conducted by a systematic literature search taking into account the CQA, CPP and CMA previously identified. However, for an ideal risk identification during product development, all of the stakeholders, such as medical doctors, designers and manufacturers, need to have a proactive attitude to identify and control potential risks to quality, having a clear understanding of the customers, market, process, and the product definition [201].

The design and manufacturing processes of custom bone prosthesis involves many steps, leading to potential mistakes that can occur in any of the workflow processes such as medical image acquisition, data transfer, segmentation process, and additive machine settings [144]. Moreover, in any of these processes, imperceptible dimensional errors can occur affecting the final product accuracy [210]. This results in inaccurate and imperceptible distorted models, leading to surgery mistakes and wrong prosthesis measurements [211].

During the image acquisition step, different kinds of errors can occur if the instructions provided by the design company and the medical doctor are not strictly followed. Therefore, it is important to follow the CT scanning protocol provided. For example, the gantry tilt, that is the angle in which the patient is scanned, should be set at zero degrees. If this this is not followed, and the gantry angle is small, it will lead to imperceptible errors in the final model [212]. Additionally, is important to not have low resolution images nor to compress the images file, because it can lead to loss of information quality and cause discrepancies [140].

One of the most common errors that occurs in the CT image acquisition process is due to a patient’s involuntary movement that can result in distorted images [211]. For example, for maxillofacial surgery as little as 1 mm of displacement leads to useless models, and if the patient is scanned with a closed bite this will result in an overlap of upper and lower teeth [211]. Moreover, metal artefacts in the patient, such as dental amalgam or gold fillings can distort the CT images and affect the 3D model [211]. So, to generate a clean model, artefacts have to be removed by editing the CT images slice by slice [212].

Furthermore, during the image segmentation process of bones, noise can be found between boundaries of dense tissues, making it difficult to perform an approximated tissue differentiation [211]. This problem can be caused by an incorrect thresholding, where the voxels along the boundaries automatically acquire a wrong threshold value [144]. This happens because a voxel can only have one threshold value, so if it is located in a boundary and is shared by two tissues, it will acquire the threshold value of the dominant tissue [144]. Therefore, this is one of the most sensitive processes in the workflow [149].

Another typical error in 3D printing technologies is the stair step effect. This kind of error affects the surface resolution of the 3D printed part and can be caused by the slice thickness of the CT images, the part building orientation, the building layer thickness of the 3D printing machine, and by a wrong approximation of the freeform shape [213]. Nevertheless, these kinds of errors can be minimised, but not be completely eliminated, by changing the CT protocol, adjusting the 3D printer machine settings, and by an optimization of the STL model [213].

In traditional manufacturing methods, process monitoring and control have been widely employed in academic and industrial applications to ensure process consistency [214]. However, in AM, the adoption of process monitoring and control have been used mainly in research [215]. This is due to the fact that AM systems involve a multitude of factors with high complexity making this task extremely challenging [216]. Some of the variables involved are: part orientation [217], layer thickness, hatch spacing, bed temperature [218], laser diameter and temperature [189, 190], bed temperature, and cooling cycle [2]. Moreover, In AM systems, machine errors and incorrect machine settings, may cause irregularities in the model [213]. Unless adequately monitored and controlled, all of these factors will lead to a potential fabrication error compromising material properties, dimensional accuracy and biological safety.

Regardless of the 3D printing system used, cleaning and finishing of post-manufacture build parts is necessary [196]. The method to clean 3D printed parts varies according to the system used for their manufacture. For example, some systems need to build parts with support structures that need to be removed later [219], while powder based systems require the cleaning of the build parts from the remaining particles of the powder bed [190]. Moreover, depending on the desired surface finish, certain finishing processes are needed, such as tumbling and sandblasting [196]. However, porous parts can be affected by imperceptible inclusions of particles inside their pores compromising their biological safety [220].

Moreover, residual stress and dimensional distortion can be caused by a uniformly heating and cooling in metals, leading to premature failure and fatigue of the 3D printed part [221]. Nevertheless, this problem can be reduced using different approaches, such as heat treatments [222], the application of ultrasonic impact treatment (UIT) [223] or it can be controlled by an optimization of the 3D printing machine settings [221]. Additionally, in 3D printing powder based technologies, the distribution of the powder bed particles can affect the surface quality of the built part. This can be caused by wear of the coater blade, short of feed power [224], and material flowability [225].

Overall, a total of 85 main causes that lead to non-conformance quality were identified and allocated into the Ishikawa diagram and Risk Breakdown Structure (RBS). To avoid some of these risks communication protocols between surgeons, radiologists and engineers are essential in conjunction with strict quality control systems during the design and manufacturing processes of 3D printed prosthesis and scaffolds.

#### Ishikawa diagram

**Fig 7:**
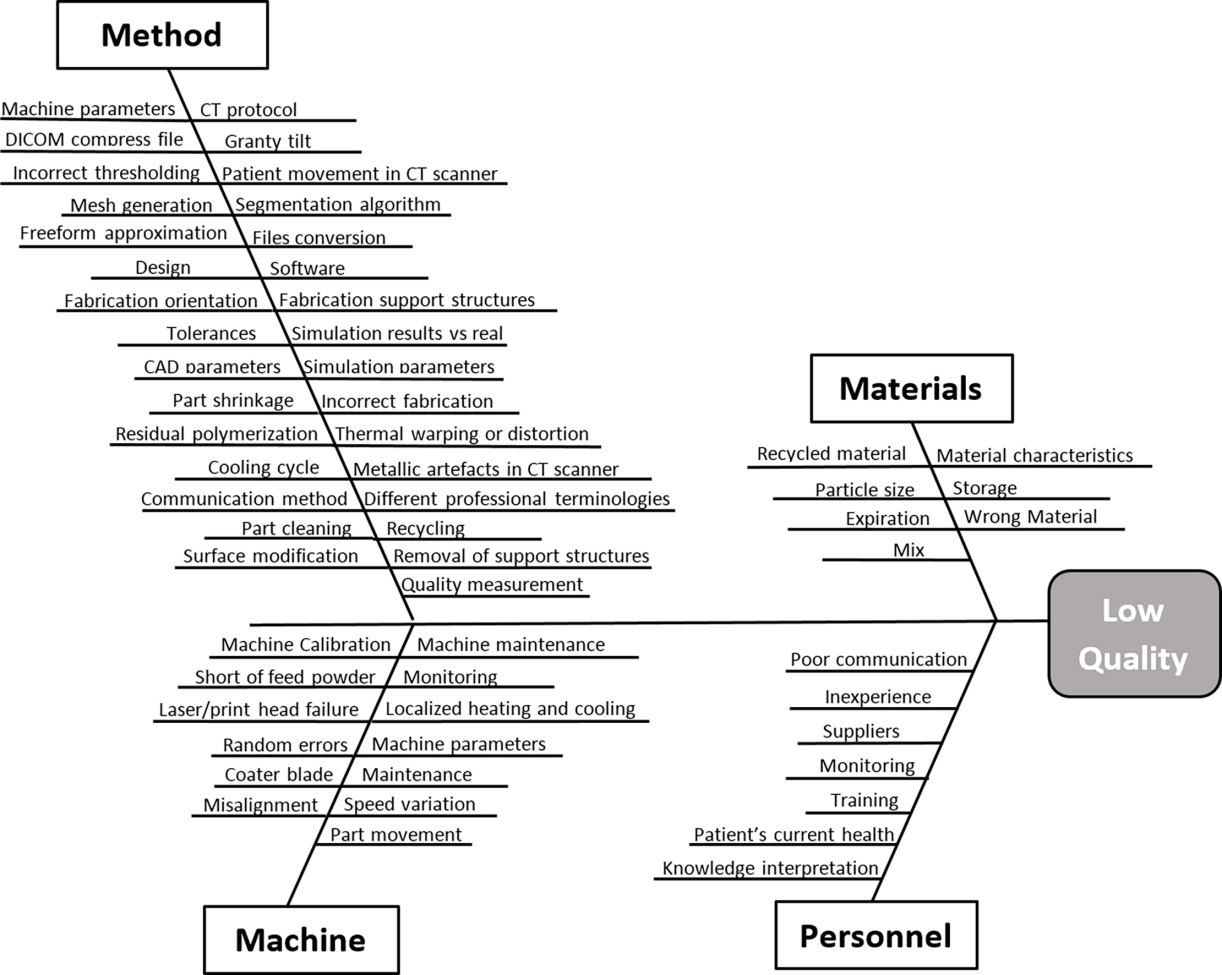
Ishikawa diagram, summary of causes of non-conformity to the quality of custom 3D printed bone implants.

In this study the Ishikawa diagram is composed of four branches. These branches are *method*, *machine*, *material*s and *people*. The *method* branch includes those factors related to the design and fabrication process. The *machine* branch compromises the machinery and equipment used in these processes. The *material* branch outlines the materials used in each process, and the *people* branch details the factors related to human resources. Using the Ishikawa diagram, all of the 86 identified risks are segregated according to the four categories of the diagram, a summary is presented in Fig 7.

#### Risk breakdown structure

The Risk Breakdown Structure (RBS) developed for this study (Table 7) consists of three hierarchical levels that categorise identified risks sources. In our RBS, each risk received a category number according to its hierarchical position that facilitates further risk assessment analysis. Therefore, the first hierarchical level (Level zero) of the RBS represents all the risks of non-conformance that affect the CQA of custom 3D printed bone prostheses and scaffolds. The second hierarchical level (Level 1) compromises the risks that belong to the four branches of the previous Ishikawa diagram, which are *Method*, *Machine*, *Material*, and *Personnel*. The third hierarchical level (Level 2) includes each sub-process presented in the design and fabrication workflow map that can affect the desired quality. The last columns of the RBS belongs to the fourth hierarchical level (Level 3). These columns contains the list of the 86 identified risks to non-conformance quality, including 178 effects on product quality. Additionally, to each risk was assigned a code number to facilitate its identification.

**Table 7:** RBS of the causes of quality non-conformity in custom 3D printed bone prostheses.

Level 0	Level 1	Level 2	Level 3			
**Non-conformance risks**	**1. Method**	**1. CT protocol**	**Code**	**Risk**	**Effect**	**Ref.**
			1.1.1	Difficulty to implement CT protocol. Use of wrong CT parameters	• Model dimension distortion	[149, 226]
			1.1.2	Slice increment is too large or the slice thickness is too big	• Stair step effect • Rough dissolved surface • Fail to capture thin bone (mainly in facial structures such as orbital walls) • Smooths out sharp corners greatly affecting the accuracy of sharp vertices or acute edges.	[210, 212, 213, 226–230]
			1.1.3	Small radiation dose compared to slice thickness	• Noise in images leads to wrong 3D reconstruction	[228]
		**2. Image acquisition**	1.2.1	Wrong gantry tilt	• Distortion of 3D volumetric reconstruction	[132, 212, 227]
			1.2.2	Patient involuntary movement during CT scan	• Discrepancies in CT images • Distortion of 3D volumetric reconstruction	[211, 227, 230]
			1.2.3	Metallic artefacts	• Distortion of 3D volumetric reconstruction	[132, 211, 227, 230]
			1.2.4	Compressed file or wrong file format	• Discrepancies in CT images • Low quality image resolution	[140]
		**3. Image segmentation**	1.3.1	Incorrect thresholding or algorithm processing	• Fail to capture thin bone (mainly in facial structures such as orbital walls) • Larger or smaller models due to wrong boundaries • Noise in 3D volumetric reconstruction • Dimensional variations in the model	[140, 144, 149, 210, 212, 226, 227]
		**4. 3D volumetric reconstruction**	1.4.1	Incorrect mesh generation	• Dimensional variations in the model • Noise in 3D volumetric reconstruction • Loss of data • Fail to capture thin bone	[140, 144, 149, 226]
			1.4.2	Incorrect mesh optimization or refinement	• Poor and rough surface quality	[149, 212]
			1.4.3	Software used	• Dimensional variations in the model • Noise in 3D volumetric reconstruction • Loss of data • Fail to capture thin bone	[140, 144, 149, 226]
			1.4.4	Conversion from DICOM to STL.	• Dimensional variations in the model • Noise in 3D volumetric reconstruction • Loss of data • Fail to capture thin bone	[140, 144, 149, 226]
		**5. Design**	1.5.1	Software: file conversion between STL and CAD	• Loss of part details such as thin bone of the orbital wall, due to incomplete data transfer during file conversion • Loss of thin bone • Floating regions	[226, 227, 230]
			1.5.2	Wrong freeform approximation	• Stair step effect	[213]
			1.5.3	Wrong implant/scaffold design	• Inclusions of particles inside closed cavities	[190]
			1.5.4	Wrong design (surface and unit cell)	• Wrong implant mechanical properties for soft-hard tissue contact adaptation (modulus of elasticity)	[78, 108]
			1.5.5	Close tolerances	• Wrong tolerances of the fabricated part due to tolerances being geometry dependent	[190]
			1.5.6	Wrong scaffold pore design (unit cell), such as size, shape, and interconnection	• Insufficient cell density and bone regeneration	[102, 108]
			1.5.7	Insufficient support structures	• Part or surface damage	[230]
			1.5.8	Wrong tolerances	• Fusion of trapped particles	[190]
			1.5.9	Incorrect material or design parameters	• Part shrinkage and distortion	[213]
			1.5.10	Incorrect part orientation for fabrication	• Stair step effect • Undesired surface quality • Orthotropic or transvers isotropic mechanical behaviour • Dimensional inaccuracy • Bad surface quality • Risk of warping • Can increase fabrication time • Leads to dimensional inaccuracies due to post machining	[213, 217, 231–235]
		**6. Simulation and FEA**	1.6.1	Discrepancies between computational simulation and experimental data, due to inaccurate micro precision in fabrication	• Inaccurate mechanical and biological properties as well as fluid dynamics	[233]
			1.6.2	Errors in joint kinematics estimation	• May affect load computations.	[158, 236]
			1.6.3	Inaccurate estimation of bone contact forces	• Implant failure	[237, 238]
			1.6.4	Inaccurate estimation of bone/implant contact forces	• Inaccurate estimation of micromotion and stability between bone and implant	[237, 239]
			1.6.5	Incorrect FEA parameters	• Wrong design • Wrong design optimization • Wrong implant prediction of mechanical behaviour	[239]
		**7. Fabrication**	1.7.1	Inaccurate fabrication of micro-features such as pore size and shape	• Reduced biological and mechanical performance • Defective product	[233]
			1.7.2	Localised material heating and cooling.	• Thermal warping or dimensional distortion • Residual stress • Material microstructure variation	[2, 221–223, 240, 241]
			1.7.3	Material contamination	• Defective product due to impurities higher than max limits	[226]
			1.7.4	Part overhanging features	• Undesirable defects	[164]
			1.7.5	Residual polymerization	• Inaccurate parts	[227]
			1.7.6	Fabrication layer thickness.	• Stair step effect in Z direction • Material density • Surface roughness	[149, 212, 213, 218, 240, 242]
			1.7.7	Large scanning spacing or hatch spacing	• Stair step effect in X and Y directions, leading to dimensional inaccuracy • Insufficient bonding between scan tracks • Poor mechanical properties	[213, 218, 235, 240]
			1.7.8	Low scanning spacing or hatch spacing	• Slowdown the fabrication process • Increases amount of energy require • Increases fabrication cost	[235]
			1.7.9	Laser diameter	• Omission of part fine details	[190, 227]
			1.7.10	High laser temperature	• Part shrinkage • Fusion of surrounding particles • Over-curing or over-sintering	[189, 213]
			1.7.11	Low laser temperature	• Slow fabrication process • Increases fabrication cost	[235]
			1.7.12	Powder bed temperature	• Variation on part density and mechanical properties • Age hardening of material	[218, 235, 243]
			1.713	Material thermal and phase change effects	• Part shrinkage	[240]
			1.7.14	Incorrect cooling cycle	• Thermal warping • Changes in colour • Variation in material microstructure. • Damage of unsintered powder	[2, 190]
			1.7.15	Part exposition to atmosphere when still at high temperature	• Changes in colour	[190]
			1.7.16	Different professional terminologies	• Defective product • Misinterpretation of different terminologies from the different fields involved.	[118]
			1.7.17	Process documentation and expression of documents	• Defective product. • Inaccurate product definition • Inefficient workflow • Miscommunication and difficulty to understand different terminologies from the different fields involved	[118]
			1.7.18	Communication method (technology used)	• Defective product • Inefficient workflow • Inappropriate interpretation of the transferred knowledge	[118, 244]
			1.7.19	Difficulty to monitor fabrication process	• Can leads to reduced quality • Process inconsistency • Unreliability	[31, 215, 218, 245, 246]
			1.7.20	Inaccurate/inefficient material recycling method	• In powdered materials this can lead to larger particle size, contamination, and oxidation.	[2, 3, 188, 190]
		**8. Finishing**	1.8.1	Removal of supporting structures	• Part damage • Lead to surface roughness where support structures were allocated	[212, 227]
			1.8.2	Part cleaning (Sand blasting)	• Part damage (changes in part dimensions) • Inclusions of particles in porous structures (biological contamination)	[166, 190, 220, 247]
			1.8.3	Surface modification method	• Discrepancy in mechanical behaviour	[121]
		**9. Surface topography modification**	1.9.1	Surface topography modification parameters.	• Wrong biological performance • Biofilm formation	[83, 106, 107, 248]
			1.9.2	Selection of surface topography modification method.	• Different biological performance • Biofilm formation	[83, 249]
		**10. Implant dimensional validation**	1.10.1	Difficulty locating land marks to measure the model.	• Wrong measurement of model	[118, 227]
			1.10.2	Human error during measurement	• Wrong measurement of model	[118, 227]
			1.10.3	Dimensional validation method	• Wrong measurement of model	[118, 227]
		**11. Sterilization**	1.11.1	Wrong selection of sterilization method	• Implant dimensional changes • Alteration of surface chemistry and morphology	[114, 250]
			1.11.2	Inefficient sterilization	• Biological hazard, such as viral and microbial transmission • Leads to revision surgery	[197, 251, 252]
	**2. Machine**		2.1	Building speed variation	• Inaccurate part dimensions • Defective part • Higher cooling rates • Higher material porosity	[213, 235]
			2.2	Misalignment of positioning system	• Inaccurate part dimensions • Defective part	[213]
			2.3	Part movement during fabrication	• Inaccurate part dimensions • Defective part	[213]
			2.4	Clogged print head or Nuzzle	• Damaged or defective part	[213, 218]
			2.5	Worn coater blade	• Low surface quality	[224]
			2.6	Short of feed powder	• Low surface quality	[224]
			2.7	Laser failure	• Internal defects • Undesirable porosities	[220, 253]
			2.8	Random errors in parts made by the same process, using the same material and parameters	• No identical implants	[240]
			2.9	Localised material heating and cooling	• Thermal warping • Dimensional distortion • Residual stress • Material phase change effects	[2, 221–223, 240, 241]
			2.10	Machine maintenance	• Equipment failure • Inefficiency	[164, 254]
			2.11	Machine calibration	• Dimensional inaccuracies • Low quality parts	[164, 255]
			2.12	Machine parameters	• Dimensional accuracy • Low quality parts • Unreliable mechanical properties	[164, 218, 235]
			2.13	Difficulty to monitor fabrication process	• Can leads to reduced quality • Process inconsistency • Unreliability	[31, 215, 218, 245, 246]
	**3. Personnel**		3.1	Poor communication between design team and surgeon	• Inaccurate product • Difficulty to predict surgery risks and results	[256, 257]
			3.2	Misinterpretation of the transferred knowledge	• Defective product • Inefficient workflow • Inaccurate product definition	[118]
			3.3	Availability of high qualified personal in the necessary professional skills	• Can lead to low performance • Difficulties in product development	[17, 258]
			3.4	Material Suppliers	• Low material quality	[259]
			3.5	Fabrication/design suppliers	• Low quality	[118, 259]
			3.6	Deficient personnel traits and training	• High chances of mistakes during design and fabrication processes, leading to low quality products	[17]
			3.7	Lack of training and experience due to introduction of new technologies and surgical methods (medical doctor)	• Inaccurate use of implant Higher surgery risk	[257, 260]
			3.8	Low stakeholders involvement during the product development and design process	• Incorrect product characteristics	[260, 261]
			3.9	Limited knowledge of patient’s current health condition an biological characteristics	• Poor preoperative planning • Iatrogenic trauma • Concomitant injuries and illnesses • Longer operation time • Increment of revision surgeries • Higher infection rate • Decreased of bone healing rate	[152, 262]
	**4. Materials**		4.1	Material stored under sun light and humid places	• Damage material and contamination • Distinct mechanical properties	[7, 213]
			4.2	Expired material	• Inconsistent mechanical properties • Inconsistent printing quality	[213]
			4.3	Wrong material mix (% of virgin and % of recycled)	• Inconsistent mechanical properties	[189]
			4.4	Wrong powder particle size	• Incorrect particle bonding, • Weaken part areas. • Incorrect powder material flowability • Decreases fabrication resolution	[149, 188, 190, 242, 263]
			4.5	Wrong material characteristics and contamination	• Inconsistent mechanical properties, not compliance with regulations	[164, 188, 190]
			4.6	Wrong material selection	• Reduced implant biocompatibility • Reduced life span	[264]
			4.7	Material reuse times	• Can lead to contamination • Increment in oxygen content, • Less spherical and rougher powder particles • Increase in powder flowability, Inconsistent mechanical properties	[2, 189, 190, 199]

The code consists in a number of 3 digits (***i*.*j*.*k***) if the risk belongs to the Ishikawa branch *Method*, in Level 1, However, for the risks that belong to the Ishikawa branches *Machine*, *Personnel*, and *Material* the code number is compose by two digits (***i*.*k***). The first digit *“****i****”* refers to the Ishikawa branch were the risk is allocated. The second digit *“****j****”* references the sub-process of the workflow map where the risk was identified (note that for *Machine*, *Personnel*, and *Material* the digit *“****j****”* is not present). The last digit *“****k****”* is the enumeration of each risk belonging to a sub-process or to one of the Ishikawa branches in the case of *Machine*, *Personnel*, and *Material*.

#### Interviews

A total of six semi-structured interviews were performed in October 2017. To maintain the participants’ confidentiality and anonymity a code was assigned to each of them as A, B, C, D, E, and F. Overall, the participants’ expertise comprised a good mix of researchers, industry experts, and medical practitioners from different fields related to medical device development, 3D bone printed implants, motion capture, tissue engineering, orthopaedic surgery, bone biomechanics, computational neuromuscular modeling, and nano engineered implants. The duration of each interview ranged from 30 to 90 minutes. Four sets of face to face interviews were conducted in Australia, and two via video conference in Australia and USA, (see supplementary material in S2 File). All interviews were carried out by the first author of this paper on a one-to-one basis.

Overall, the interviewees agree that adapted QbD system of this study can be used to achieve the same benefits that the adapted QbD system proposed in this study has strong potential to achieve the same benefits that have been delivered in the pharmaceutical sector. These four benefits were previously identified in the prior QbD study statistical analysis (see supplementary material in S1 File), namely: process understanding (PU), prediction and optimization (PO), reduction of experimental runs (RER), and development of robust manufacturing methods (DRM). Moreover, during the interviews the participants discussed, opportunities and benefits of applying QbD in this field.

According to the opinions of participating experts, one of the potential benefits that QbD can offer for the development of such products is clearer direction for the product development process by providing in-depth understanding of the numerous factors involved in it, allowing a better definition of product boundaries, targets, and potential modifications. Moreover, according to participants’ comments the results of the adaptation of the QbD can enhance current design practices and accelerate product development by breaking down the whole development process into easily understandable sections that can independently be developed under the same goal. This will result in a more efficient process ultimately leading to a reduction in product development time, and risk. Furthermore, a good feature of QbD is that it “*can act as an insurance for innovation to be truly examined and analysed”* (Participant A). Moreover, *“having the backing of QbD it will definitely assist innovation towards commercialization”* (Participant E). All this can be possible due to the fact that QbD is a tool that facilitates a complete understanding of the product and its fabrication processes, requiring less testing of products and ideas to find optimal ranges of operation that then can be extrapolated to invent new products.

Other potential benefits identifies by interviewees was that QbD can facilitate communication between experts, because it breaks down the whole development process into coherent and manageable elements. Moreover, it was mentioned that the use of QbD and computational modelling will be critical for the development of personalised biological implants. Furthermore, three participants identified that there is a need for regulations in this emerging area and QbD would be a good framework to guide relevant regulatory bodies (e.g. FDA in the USA and TGA in Australia) to develop new quality standards for emerging customized medical products. One interviewee summarised their perceived benefits of a structured QbD framework in the following statement *“they can function better*, *break less*, *and give better outcomes”* (Participant C).

## Discussion

### Implications for researchers, practitioners, and industry

In this study we have provided a foundation for future studies to adapt the QbD approach for 3D printed bone implants. Therefore, our study offers the opportunity to refine and validate the concepts and constructs that emerged from it. Moreover, this early adaptation of the QbD approach can be used to assist and guide the early planning of the design and prototyping phases of any custom 3D printed bone implant in order to optimize research activities and resources, while controlling risks, improving quality and reducing costs.

In addition, practitioners can benefit from this research because 3D printing and QbD are providing the opportunity to design better products utilizing their experienced input in the design process. This represents an opportunity for specialists to compete, and be part of this growing market. Consequently, the future of the custom bone implants market can be driven by medical doctors with design skills and a deep product understanding to maintain a constant feedback for product improvement, based on their direct contact with patients.

Furthermore, the QbD approach can provide well-established design and manufacturing procedures to the 3D printed bone implants industry as has been shown in the pharmaceutical sector. Consequently, these robust procedures can be translated into quality improvement and reduced failure rates. Moreover, this adaptation of QbD will help the industry to optimize resources and accelerate product development time frames for 3D printed bone implants.

### Future directions

Future work seeks to: build the foundation of the three remaining steps of the QbD framework; generate detailed specifications for all elements of each step of the QbD framework for specific types of 3D printed bone implants; refine and quantify risks through a structured interview process; and finally implement the QbD framework (i.e. case study implementation). Collaboration with researchers, medical practitioners, additive manufacturing experts, and other appropriate industry practitioners is required to complete these pertinent research activities.

### Limitations

The information available for this study was limited by the relatively recent repository of data available in peer reviewed journals compiled for the systematic search process, and by the challenge of obtaining opinion from the small community of experts working in this emerging field. To generate a more comprehensive QbD adaptation and risks assessment, it is necessary to collaborate with a large multidisciplinary team of specialists in this area, such as researchers, engineering designers, manufacturers and practitioners; this work will be subject for future research by the authors. However, despite these limitations, this current study was able to formulate and validate the architecture for the first five steps for the QbD framework, develop a detailed work-flow map of key processes, identify critical risks, and accurately allocate these risks to their sources and potential impacts.

### Concluding remarks

Additive manufacturing is a paradigm shift for the medical device industry, innovatively changing the way that customized bone prostheses and scaffolds are designed and manufactured so as to extend product life and fulfil each customer’s needs. However, this new technology in the medical market is limited by a lack of standards and quality assurance, leading to a deficiency in confidence by practitioners and consumers. Additionally, these issues, in conjunction with unproductive trial-and-error studies, are leading innovative products to die prematurely in the ‘Valley of Death’ due to high development costs, preventing new developments reaching pre-clinical and clinical studies to demonstrate product safety, and obtain regulatory approval. To address some of these problems, we propose the implementation of the QbD approach as it provides a strong framework for guiding research and practice in this emerging manufacturing technology and paves the way to ensure error-free 3D printed bone implants for consumers.

The aim of this study was to formulate and validate the first five steps of the QbD approach for the early development of custom 3D printed bone implants. The main outcomes of this work are: a detailed workflow map for the design and fabrication of custom 3D printed bone implants; and a RBS, which describes 86 risks that lead to quality non-conformances, and the associated 178 potential undesirable effects on product quality. The workflow map proposed by this study was developed to provide a more detailed picture of the activities involved in each process. This will aid future studies to take the most feasible route according to the implant’s desired outcomes, complexity and chosen characteristics. The RBS developed in this study will facilitate future studies by identifying the most critical areas or activities related to the design and fabrication processes of such products and to be able to formulate adequate strategies and contingency plans to control these risks. Therefore, the developed QbD framework, in conjunction with adequate quality assurance technologies, can help to achieve better product and process understanding in order to develop within the established limits more robust design and manufacturing methods for 3D printed bone prostheses and scaffolds, as has been proven in the pharmaceutical sector. Consequently, these robust procedures can be translated into quality improvement and reduced failure rates, which will lead to heightened confidence in this emerging manufacturing technology. However, to achieve this, industry, researchers, and practitioners need to be greatly involved. Consequently, collaborative efforts are needed for the development and sharing of best practices in order to promote the use of this technology to foster innovation in this area.

## Supporting information

S1 File QbD study descriptive statistics. (PDF)

S2 File Semi-structured interviews transcripts and analysis. (PDF)

S3 File Systematic search data availability statement. (PDF)

S1 Database QbD study descriptive statistics dataset. (XLSX)

S2 Database Systematic search dataset. (XLSX)

## References

[1] Xue C, Yang Y, Bao B. Evaluation of product customization customer satisfaction. International Journal of Advancements in Computing Technology. 2012;4(20).

[2] Murr L, Quinones S, Gaytan S, Lopez M, Rodela A, Martinez E, et al Microstructure and mechanical behavior of Ti–6Al–4V produced by rapid-layer manufacturing, for biomedical applications. Journal of the mechanical behavior of biomedical materials. 2009;2(1):20–32. doi: 10.1016/j.jmbbm.2008.05.004 19627804 10.1016/j.jmbbm.2008.05.004

[3] Prince JD. 3D Printing: An industrial revolution. Journal of Electronic Resources in Medical Libraries. 2014;11(1):39–45.

[4] Mazzoli A. Selective laser sintering in biomedical engineering. Medical & biological engineering & computing. 2013;51(3):245–56. 23250790 10.1007/s11517-012-1001-x

[5] Ho CMB, Ng SH, Yoon Y-J. A review on 3D printed bioimplants. International Journal of Precision Engineering and Manufacturing. 2015;16(5):1035–46.

[6] Bhatia SK, Sharma S. 3D-Printed prosthetics roll off the presses. Chemical Engineering Progress. 2014;110(5):28–33.

[7] Pham D, Gault R. A comparison of rapid prototyping technologies. International Journal of Machine Tools and Manufacture. 1998;38(10):1257–87.

[8] Fan H, Fu J, Li X, Pei Y, Li X, Pei G, et al Implantation of customized 3-D printed titanium prosthesis in limb salvage surgery: a case series and review of the literature. World journal of surgical oncology. 2015;13(1):1. 26537339 10.1186/s12957-015-0723-2 PMC4632365

[9] Imanishi J, Choong PF. Three-dimensional printed calcaneal prosthesis following total calcanectomy. International journal of surgery case reports. 2015;10:83–7. doi: 10.1016/j.ijscr.2015.02.037 25827294 10.1016/j.ijscr.2015.02.037 PMC4429954

[10] Kroonenburgh I, Beerens M, Engel C, Mercelis IP, Lambrichts I, Poukens J. Doctor and engineer creating the future for 3D printed custom made implants. Digital Dental News. 2012;6:60–5.

[11] Denry I, Kuhn LT. Design and characterization of calcium phosphate ceramic scaffolds for bone tissue engineering. Dental Materials. 2016;32(1):43–53. doi: 10.1016/j.dental.2015.09.008 26423007 10.1016/j.dental.2015.09.008 PMC4696887

[12] Lantada AD, Morgado PL. Rapid prototyping for biomedical engineering: current capabilities and challenges. Annual review of biomedical engineering. 2012;14:73–96. doi: 10.1146/annurev-bioeng-071811-150112 22524389 10.1146/annurev-bioeng-071811-150112

[13] Monzón M, Ortega Z, Martínez A, Ortega F. Standardization in additive manufacturing: activities carried out by international organizations and projects. The International Journal of Advanced Manufacturing Technology. 2015;76(5–8):1111–21.

[14] Yeong W, Chua C, editors. Implementing additive manufacturing for medical devices: A quality perspective. High Value Manufacturing: Advanced Research in Virtual and Rapid Prototyping: Proceedings of the 6th International Conference on Advanced Research in Virtual and Rapid Prototyping, Leiria, Portugal, 1–5 October, 2013; 2013: CRC Press.

[15] U.S. Food and Drug Administration. Medical device innovation initiative white paper: CDRH innovation initiative2011.

[16] Asiedu Y, Gu P. Product life cycle cost analysis: state of the art review. International journal of production research. 1998;36(4):883–908.

[17] Russell RK, Tippett DD. Critical success factors for the fuzzy front end of innovation in the medical device industry. Engineering Management Journal. 2008;20(3):36–43.

[18] Brown A, Dixon D, Eatock J, Meenan BJ, Young T, editors. A survey of success factors in new product development in the medical devices industry. 2008 IEEE International Engineering Management Conference; 2008: IEEE.

[19] Hollister SJ. Scaffold engineering: a bridge to where? Biofabrication. 2009;1(1):012001 doi: 10.1088/1758-5082/1/1/012001 20811095 10.1088/1758-5082/1/1/012001

[20] Hollister SJ. Scaffold design and manufacturing: from concept to clinic. Advanced Materials. 2009;21(32‐33):3330–42. doi: 10.1002/adma.200802977 20882500 10.1002/adma.200802977

[21] Larson EW, Gray C. Project management–The managerial process 5th ed. McGraw-Hill/Irwin, New York; 2011 p. 211.

[22] Henkel J, Woodruff MA, Epari DR, Steck R, Glatt V, Dickinson IC, et al Bone regeneration based on tissue engineering conceptions—a 21st century perspective. Bone research. 2013;1(3):216 doi: 10.4248/BR201303002 26273505 10.4248/BR201303002 PMC4472104

[23] Sipe JD. Tissue Engineering and Reparative Medicine. Annals of the New York Academy of Sciences. 2002;961(1):1–9. doi: 10.1111/j.1749-6632.2002.tb03040.x 12081856 10.1111/j.1749-6632.2002.tb03040.x

[24] Krucoff MW, Brindis RG, Hodgson PK, Mack MJ, Holmes JDR. Medical device innovation: prospective solutions for an ecosystem in crisis. Adding a professional society perspective. JACC Cardiovascular interventions 2012;5(7):790 doi: 10.1016/j.jcin.2012.03.023 22814785 10.1016/j.jcin.2012.03.023

[25] Cooper RG, Kleinschmidt EJ. An investigation into the new product process: steps, deficiencies, and impact. Journal of product innovation management. 1986;3(2):71–85.

[26] McConnell J, Nunnally BK, McGarvey B. The forgotten origins of quality by design. Journal of Validation Technology. 2010;16(3):30.

[27] Fahmy R, Kona R, Dandu R, Xie W, Claycamp G, Hoag SW. Quality by design I: application of failure mode effect analysis (FMEA) and Plackett–Burman design of experiments in the identification of “main factors” in the formulation and process design space for roller-compacted ciprofloxacin hydrochloride immediate-release tablets. AAPS PharmSciTech. 2012;13(4):1243–54. doi: 10.1208/s12249-012-9844-x 22993122 10.1208/s12249-012-9844-x PMC3513475

[28] Naidu NVR, Babu KM, Rajendra G, ebrary I. Total quality management New Delhi: New Age International (P) Ltd., Publishers; 2006.

[29] Vogt FG, Kord AS. Development of quality‐by‐design analytical methods. Journal of pharmaceutical sciences. 2011;100(3):797–812. doi: 10.1002/jps.22325 21280050 10.1002/jps.22325

[30] Lawrence XY. Pharmaceutical quality by design: product and process development, understanding, and control. Pharmaceutical research. 2008;25(4):781–91. doi: 10.1007/s11095-007-9511-1 18185986 10.1007/s11095-007-9511-1

[31] Straub J. Initial work on the characterization of additive manufacturing (3D Printing) using software image analysis. Machines. 2015;3(2):55–71.

[32] Sangshetti JN, Deshpande M, Zaheer Z, Shinde DB, Arote R. Quality by design approach: Regulatory need. Arabian Journal of Chemistry. 2014.

[33] ICH Harmonised Tripartite Guideline. Pharmaceutical development Q8 (R2) ICH Steering Committee, Step 2009;4.

[34] Cogdill RP, Drennen JK. Risk-based quality by design (QbD): A taguchi perspective on the assessment of product quality, and the quantitative linkage of drug product parameters and clinical performance. Journal of Pharmaceutical Innovation. 2008;3(1):23–9. doi: 10.1007/s12247-008-9025-3

[35] Oyegoke A. The constructive research approach in project management research. International Journal of Managing Projects in Business. 2011;4(4):573–95. doi: 10.1108/17538371111164029

[36] Kasanen E, Lukka K, Siitonen A. The constructive approach in management accounting research. Journal of Management Accounting Research. 1993;5:243.

[37] Liberati A, Altman DG, Tetzlaff J, Mulrow C, Gøtzsche PC, Ioannidis JP, et al The PRISMA statement for reporting systematic reviews and meta-analyses of studies that evaluate health care interventions: explanation and elaboration. Annals of internal medicine. 2009;151(4):W-65–W-94. 19622512 10.7326/0003-4819-151-4-200908180-00136

[38] Maylor H, Blackmon KL. Researching business and management New York;Houndmills, Basingstoke, Hampshire;: Palgrave Macmillan; 2005.

[39] Harrell MC, Bradley MA. Data collection methods Semi-structured interviews and focus groups. DTIC Document, 2009.

[40] Tong A, Sainsbury P, Craig J. Consolidated criteria for reporting qualitative research (COREQ): a 32-item checklist for interviews and focus groups. International Journal for Quality in Health Care. 2007;19(6):349–57. doi: 10.1093/intqhc/mzm042 17872937 10.1093/intqhc/mzm042

[41] Anderson C. Presenting and evaluating qualitative research. American journal of pharmaceutical education. 2010;74(8). 10.5688/aj7408141 PMC2987281 21179252

[42] Yin RK. Case study research: design and methods Fifth ed. Los Angeles: SAGE; 2014.

[43] Eisenhardt KM. Building Theories from Case Study Research. The Academy of Management Review. 1989;14(4):532–50. doi: 10.5465/AMR.1989.4308385

[44] Stemler S. An overview of content analysis. Practical assessment, research & evaluation. 2001;7(17):137–46.

[45] Miles MB, Huberman AM, Saldaña J. Qualitative data analysis: a methods sourcebook Third ed. Thousand Oaks, Califorinia: SAGE Publications, Inc; 2014.

[46] McCutcheon DM, Meredith JR. Conducting case study research in operations management. Journal of Operations Management. 1993;11(3):239–56. doi: 10.1016/0272-6963(93)90002-7

[47] Zhang L, Yan B, Gong X, Lawrence XY, Qu H. Application of quality by design to the process development of botanical drug products: a case study. Aaps Pharmscitech. 2013;14(1):277–86. doi: 10.1208/s12249-012-9919-8 23297167 10.1208/s12249-012-9919-8 PMC3581681

[48] Hubert C, Lebrun P, Houari S, Ziemons E, Rozet E, Hubert P. Improvement of a stability-indicating method by Quality-by-Design versus Quality-by-Testing: A case of a learning process. Journal of pharmaceutical and biomedical analysis. 2014;88:401–9. doi: 10.1016/j.jpba.2013.09.026 24176744 10.1016/j.jpba.2013.09.026

[49] Badawy SI, Narang AS, LaMarche KR, Subramanian GA, Varia SA, Lin J, et al Integrated Application of Quality-by-Design Principles to Drug Product Development: A Case Study of Brivanib Alaninate Film–Coated Tablets. Journal of pharmaceutical sciences. 2016;105(1):168–81. doi: 10.1016/j.xphs.2015.11.023 26852852 10.1016/j.xphs.2015.11.023

[50] Raina H, Kaur S, Jindal AB. Development of efavirenz loaded solid lipid nanoparticles: Risk assessment, quality-by-design (QbD) based optimisation and physicochemical characterisation. Journal of Drug Delivery Science and Technology. 2017;39:180–91.

[51] Cun D, Jensen DK, Maltesen MJ, Bunker M, Whiteside P, Scurr D, et al High loading efficiency and sustained release of siRNA encapsulated in PLGA nanoparticles: quality by design optimization and characterization. European Journal of Pharmaceutics and Biopharmaceutics. 2011;77(1):26–35. doi: 10.1016/j.ejpb.2010.11.008 21093589 10.1016/j.ejpb.2010.11.008

[52] Verma S, Lan Y, Gokhale R, Burgess DJ. Quality by design approach to understand the process of nanosuspension preparation. International Journal of Pharmaceutics. 2009;377(1):185–98. 19446617 10.1016/j.ijpharm.2009.05.006

[53] Mazumder S, Pavurala N, Manda P, Xu X, Cruz CN, Krishnaiah YS. Quality by Design approach for studying the impact of formulation and process variables on product quality of oral disintegrating films. International Journal of Pharmaceutics. 2017. 10.1016/j.ijpharm.2017.05.048 28549972

[54] Schmidt AH, Molnár I. Using an innovative Quality-by-Design approach for development of a stability indicating UHPLC method for ebastine in the API and pharmaceutical formulations. Journal of pharmaceutical and biomedical analysis. 2013;78:65–74. doi: 10.1016/j.jpba.2013.01.032 23454599 10.1016/j.jpba.2013.01.032

[55] Dubey A, Boukouvala F, Keyvan G, Hsia R, Saranteas K, Brone D, et al Improvement of tablet coating uniformity using a quality by design approach. AAPS PharmSciTech. 2012;13(1):231–46. doi: 10.1208/s12249-011-9723-x 22232020 10.1208/s12249-011-9723-x PMC3299457

[56] Chavan SD, Pimpodkar NV, Kadam AS, Gaikwad PS. Quality by Design. Journal of Pharmaceutical Quality Assurance. 2015;1(2).

[57] FDA. Guidance for industry and review staff: target product profile-a strategic development process tool Washington, DC: US Department of Health and Human Services 2007.

[58] Lambert WJ. Considerations in developing a target product profile for parenteral pharmaceutical products. AAPS PharmSciTech. 2010;11(3):1476–81. doi: 10.1208/s12249-010-9521-x 20842540 10.1208/s12249-010-9521-x PMC2974137

[59] Sebastianelli R, Tamimi N. How product quality dimensions relate to defining quality. International Journal of Quality & Reliability Management. 2002;19(4):442–53. doi: 10.1108/02656710210421599

[60] Garvin DA. What does product quality really mean. Sloan management review. 1984;26(1).

[61] Kirk D, McClelland I, Suri JF. Involving People in Design In: Wilson JR, Sharples S, editors. Evaluation of human work: CRC Press; 2015.

[62] Sharkey PF, Sethuraman V, Hozack WJ, Rothman RH, Stiehl JB. Factors influencing choice of implants in total hip arthroplasty and total knee arthroplasty: perspectives of surgeons and patients. The Journal of arthroplasty. 1999;14(3):281–7. 10220180 10.1016/s0883-5403(99)90052-9

[63] Garvin DA. Competing on the eight dimensions of quality. Harvard Business Review. 1987.

[64] Cordonnier T, Sohier J, Rosset P, Layrolle P. Biomimetic materials for bone tissue engineering–state of the art and future trends. Advanced Engineering Materials. 2011;13(5):B135–B50.

[65] Giannoudis PV, Dinopoulos H, Tsiridis E. Bone substitutes: an update. Injury. 2005;36(3):S20–S7. 16188545 10.1016/j.injury.2005.07.029

[66] Miron R, Zhang Y. Osteoinduction a review of old concepts with new standards. Journal of dental research. 2012;91(8):736–44. doi: 10.1177/0022034511435260 22318372 10.1177/0022034511435260

[67] Cima L, Vacanti J, Vacanti C, Ingber D, Mooney D, Langer R. Tissue engineering by cell transplantation using degradable polymer substrates. Journal of biomechanical engineering. 1991;113(2):143–51. 1652042 10.1115/1.2891228

[68] Sun T, Norton D, Ryan A, MacNeil S, Haycock J. Investigation of fibroblast and keratinocyte cell-scaffold interactions using a novel 3D cell culture system. Journal of Materials Science: Materials in Medicine. 2007;18(2):321–8. doi: 10.1007/s10856-006-0696-3 17323165 10.1007/s10856-006-0696-3

[69] Lord MS, Foss M, Besenbacher F. Influence of nanoscale surface topography on protein adsorption and cellular response. Nano Today. 2010;5(1):66–78.

[70] Schmidt-Bleek K, Schell H, Schulz N, Hoff P, Perka C, Buttgereit F, et al Inflammatory phase of bone healing initiates the regenerative healing cascade. Cell and tissue research. 2012;347(3):567–73. doi: 10.1007/s00441-011-1205-7 21789579 10.1007/s00441-011-1205-7

[71] Moursi AM, Globus RK, Damsky CH. Interactions between integrin receptors and fibronectin are required for calvarial osteoblast differentiation in vitro. Journal of cell science. 1997;110(18):2187–96. 9378768 10.1242/jcs.110.18.2187

[72] Dimitriou R, Tsiridis E, Giannoudis PV. Current concepts of molecular aspects of bone healing. Injury. 2005;36(12):1392–404. doi: 10.1016/j.injury.2005.07.019 16102764 10.1016/j.injury.2005.07.019

[73] Ferraris S, Spriano S. Antibacterial titanium surfaces for medical implants. Materials Science and Engineering: C. 2016;61:965–78. 26838926 10.1016/j.msec.2015.12.062

[74] Gogolewski S. Bioresorbable polymers in trauma and bone surgery. Injury. 2000;31:D28–D32. 10.1016/s0020-1383(00)80020-0 11270078

[75] Arealis G, Nikolaou VS. Bone printing: new frontiers in the treatment of bone defects. Injury. 2015;46:S20–S2. 10.1016/S0020-1383(15)30050-4 26747913

[76] Ramin E, Harris RA. Advanced computer-aided design for bone tissue-engineering scaffolds. Proceedings of the Institution of Mechanical Engineers, Part H: Journal of Engineering in Medicine. 2009;223(3):289–301. 10.1243/09544119JEIM452 19405435

[77] Leong K, Cheah C, Chua C. Solid freeform fabrication of three-dimensional scaffolds for engineering replacement tissues and organs. Biomaterials. 2003;24(13):2363–78. 12699674 10.1016/s0142-9612(03)00030-9

[78] Suska F, Kjeller G, Tarnow P, Hryha E, Nyborg L, Snis A, et al Electron beam melting manufacturing technology for individually manufactured jaw prosthesis: A case report. Journal of Oral and Maxillofacial Surgery. 2016. 10.1016/j.joms.2016.03.046 27178123

[79] Karageorgiou V, Kaplan D. Porosity of 3D biomaterial scaffolds and osteogenesis. Biomaterials. 2005;26(27):5474–91. doi: 10.1016/j.biomaterials.2005.02.002 15860204 10.1016/j.biomaterials.2005.02.002

[80] Jahed Z, Molladavoodi S, Seo BB, Gorbet M, Tsui TY, Mofrad MR. Cell responses to metallic nanostructure arrays with complex geometries. Biomaterials. 2014;35(34):9363–71. doi: 10.1016/j.biomaterials.2014.07.022 25123921 10.1016/j.biomaterials.2014.07.022

[81] Barrere F, Mahmood T, De Groot K, Van Blitterswijk C. Advanced biomaterials for skeletal tissue regeneration: Instructive and smart functions. Materials Science and Engineering: R: Reports. 2008;59(1):38–71.

[82] Bagno A, Di Bello C. Surface treatments and roughness properties of Ti-based biomaterials. Journal of Materials Science: Materials in Medicine. 2004;15(9):935–49. doi: 10.1023/B:JMSM.0000042679.28493.7f 15448401 10.1023/B:JMSM.0000042679.28493.7f

[83] Amin Yavari S, Loozen L, Paganelli FL, Bakhshandeh S, Lietaert K, Groot JA, et al Antibacterial Behavior of Additively Manufactured Porous Titanium with Nanotubular Surfaces Releasing Silver Ions. ACS applied materials & interfaces 2016;8(27):17080. 27300485 10.1021/acsami.6b03152

[84] Liu X, Chu PK, Ding C. Surface modification of titanium, titanium alloys, and related materials for biomedical applications. Materials Science and Engineering: R: Reports. 2004;47(3):49–121.

[85] Li WJ, Laurencin CT, Caterson EJ, Tuan RS, Ko FK. Electrospun nanofibrous structure: a novel scaffold for tissue engineering. Journal of biomedical materials research. 2002;60(4):613–21. 11948520 10.1002/jbm.10167

[86] Vasconcelos DM, Santos SG, Lamghari M, Barbosa MA. The two faces of metal ions: From implants rejection to tissue repair/regeneration. Biomaterials. 2016;84:262–75. doi: 10.1016/j.biomaterials.2016.01.046 26851391 10.1016/j.biomaterials.2016.01.046

[87] Lavenus S, Ricquier J-C, Louarn G, Layrolle P. Cell interaction with nanopatterned surface of implants. Nanomedicine. 2010;5(6):937–47. doi: 10.2217/nnm.10.54 20735227 10.2217/nnm.10.54

[88] Bandhyopadhya A, Bose S. Characterization of biomaterials. Amsterdam: Elsevier; 2013.

[89] Hallab NJ, Bundy KJ, O'Connor K, Moses RL, Jacobs JJ. Evaluation of metallic and polymeric biomaterial surface energy and surface roughness characteristics for directed cell adhesion. Tissue engineering. 2001;7(1):55–71. doi: 10.1089/107632700300003297 11224924 10.1089/107632700300003297

[90] Ramsden JJ, Allen DM, Stephenson DJ, Alcock JR, Peggs G, Fuller G, et al The design and manufacture of biomedical surfaces. CIRP Annals-Manufacturing Technology. 2007;56(2):687–711.

[91] Hansen DC. Metal corrosion in the human body: the ultimate bio-corrosion scenario. The Electrochemical Society Interface. 2008;17(2):31.

[92] Lichte P, Pape H, Pufe T, Kobbe P, Fischer H. Scaffolds for bone healing: concepts, materials and evidence. Injury. 2011;42(6):569–73. doi: 10.1016/j.injury.2011.03.033 21489531 10.1016/j.injury.2011.03.033

[93] Wu S, Liu X, Yeung KW, Guo H, Li P, Hu T, et al Surface nano-architectures and their effects on the mechanical properties and corrosion behavior of Ti-based orthopedic implants. Surface and Coatings Technology. 2013;233:13–26.

[94] Salgado AJ, Coutinho OP, Reis RL. Bone tissue engineering: state of the art and future trends. Macromolecular bioscience. 2004;4(8):743–65. doi: 10.1002/mabi.200400026 15468269 10.1002/mabi.200400026

[95] Kulkarni M, Mazare A, Schmuki P, Iglič A. Biomaterial surface modification of titanium and titanium alloys for medical applications. Nanomedicine. 2014;111:111.

[96] Gulati K, Aw MS, Findlay D, Losic D. Local drug delivery to the bone by drug-releasing implants: perspectives of nano-engineered titania nanotube arrays. Therapeutic delivery. 2012;3(7):857–73. 22900467 10.4155/tde.12.66

[97] Shrivats AR, McDermott MC, Hollinger JO. Bone tissue engineering: state of the union. Drug discovery today. 2014;19(6):781–6. doi: 10.1016/j.drudis.2014.04.010 24768619 10.1016/j.drudis.2014.04.010

[98] Hollister SJ. Porous scaffold design for tissue engineering. Nature materials. 2005;4(7):518–24. doi: 10.1038/nmat1421 16003400 10.1038/nmat1421

[99] Alvarez K, Nakajima H. Metallic scaffolds for bone regeneration. Materials. 2009;2(3):790–832.

[100] Dhandayuthapani B, Yoshida Y, Maekawa T, Kumar DS. Polymeric scaffolds in tissue engineering application: a review. International Journal of Polymer Science. 2011;2011.

[101] Saini M, Singh Y, Arora P, Arora V, Jain K. Implant biomaterials: A comprehensive review. World Journal of Clinical Cases: WJCC. 2015;3(1):52 doi: 10.12998/wjcc.v3.i1.52 25610850 10.12998/wjcc.v3.i1.52 PMC4295219

[102] Taboas JM, Hollister SJ, Min Chu T, Schek RM, Lin C-Y. Design and fabrication of bone tissue engineering scaffolds Bone Tissue Engineering: CRC Press; 2005 p. 167–92.

[103] Lee M-Y, Chang C-C, Lin C-C, Lo L-J, Chen Y-R. Custom implant design for patients with cranial defects. Engineering in Medicine and Biology Magazine, IEEE. 2002;21(2):38–44. 10.1109/memb.2002.1000184 12012603

[104] Zhang LC, Klemm D, Eckert J, Hao YL, Sercombe TB. Manufacture by selective laser melting and mechanical behavior of a biomedical Ti–24Nb–4Zr–8Sn alloy. Scripta Materialia. 2011;65(1):21–4. doi: 10.1016/j.scriptamat.2011.03.024

[105] Saijo H, Igawa K, Kanno Y, Mori Y, Kondo K, Shimizu K, et al Maxillofacial reconstruction using custom-made artificial bones fabricated by inkjet printing technology. Journal of Artificial Organs. 2009;12(3):200–5. doi: 10.1007/s10047-009-0462-7 19894095 10.1007/s10047-009-0462-7

[106] Bauer S, Park J, von der Mark K, Schmuki P. TiO2 nanotubes for stimulated cell response: Control of cell-surface interactions at the nanoscale. Europ Cells Mater. 2010;20:16.

[107] Neacsu P, Mazare A, Cimpean A, Park J, Costache M, Schmuki P, et al Reduced inflammatory activity of RAW 264.7 macrophages on titania nanotube modified Ti surface. The international journal of biochemistry & cell biology. 2014;55:187–95. 25220343 10.1016/j.biocel.2014.09.006

[108] Zadpoor AA, Hedayati R. Analytical relationships for prediction of the mechanical properties of additively manufactured porous biomaterials. Journal of Biomedical Materials Research Part A. 2016. 10.1002/jbm.a.35855 PMC5129517 27502358

[109] Groen WM, Diloksumpan P, van Weeren PR, Levato R, Malda J. From intricate to integrated: Biofabrication of articulating joints. Journal of Orthopaedic Research. 2017;35(10):2089–97. doi: 10.1002/jor.23602 28621834 10.1002/jor.23602 PMC5655743

[110] Kuznetsova D, Ageykin A, Koroleva A, Deiwick A, Shpichka A, Solovieva A, et al Surface micromorphology of cross-linked tetrafunctional polylactide scaffolds inducing vessel growth and bone formation. BIOFABRICATION. 2017;9(2). doi: 10.1088/1758-5090/aa6725 28300041 10.1088/1758-5090/aa6725

[111] Fernandez J, Zhang J, Heidlauf T, Sartori M, Besier T, Rohrle O, et al Multiscale musculoskeletal modelling, data—model fusion and electromyography-informed modelling. INTERFACE FOCUS. 2016;6(2):20150084 doi: 10.1098/rsfs.2015.0084 27051510 10.1098/rsfs.2015.0084 PMC4759749

[112] Pizzolato C, Lloyd DG, Barrett RS, Cook JL, Zheng MH, Besier TF, et al Bioinspired Technologies to Connect Musculoskeletal Mechanobiology to the Person for Training and Rehabilitation. Frontiers in Computational Neuroscience. 2017;11(96). doi: 10.3389/fncom.2017.00096 29093676 10.3389/fncom.2017.00096 PMC5651250

[113] Colligan L, Anderson JE, Potts HW, Berman J. Does the process map influence the outcome of quality improvement work? A comparison of a sequential flow diagram and a hierarchical task analysis diagram. BMC health services research. 2010;10(1):1. 20056005 10.1186/1472-6963-10-7 PMC2822834

[114] Hieu LC, Zlatov N, Vander Sloten J, Bohez E, Khanh L, Binh PH, et al Medical rapid prototyping applications and methods. Assembly Automation. 2005;25(4):284–92. doi: 10.1108/01445150510626415

[115] Sun W, Starly B, Nam J, Darling A. Bio-CAD modeling and its applications in computer-aided tissue engineering. Computer-Aided Design. 2005;37(11):1097–114. doi: 10.1016/j.cad.2005.02.002

[116] Rahmati S, Farahmand F, Abbaszadeh F. Application of rapid prototyping for development of custom–made orthopedics prostheses: an investigative study. International Journal of Advanced Design and Manufacturing Technology. 2011;3(2):11–6.

[117] Dérand P, Rännar L-E, Hirsch J-M. Imaging, virtual planning, design, and production of patient-specific implants and clinical validation in craniomaxillofacial surgery. Craniomaxillofacial trauma & reconstruction. 2012;5(3):137. 23997858 10.1055/s-0032-1313357 PMC3578652

[118] Salmi M, Tuomi J, Paloheimo K-S, Björkstrand R, Paloheimo M, Salo J, et al Patient-specific reconstruction with 3D modeling and DMLS additive manufacturing. Rapid Prototyping Journal. 2012;18(3):209–14.

[119] Podshivalov L, Gomes CM, Zocca A, Guenster J, Bar-Yoseph P, Fischer A. Design, analysis and additive manufacturing of porous structures for biocompatible micro-scale scaffolds. Procedia CIRP. 2013;5:247–52.

[120] Chae MP, Rozen WM, McMenamin PG, Findlay MW, Spychal RT, Hunter-Smith DJ. Emerging applications of bedside 3D printing in plastic surgery. Frontiers in surgery. 2015;2. 26137465 10.3389/fsurg.2015.00025 PMC4468745

[121] Chahine G, Koike M, Okabe T, Smith P, Kovacevic R. The design and production of Ti-6Al-4V ELI customized dental implants. Jom. 2008;60(11):50–5.

[122] Hollister SJ, Levy RA, Chu TM, Halloran JW, Feinberg SE. An image‐based approach for designing and manufacturing craniofacial scaffolds. International Journal of Oral & Maxillofacial Surgery. 2000;29(1):67–71. 10.1034/j.1399-0020.2000.290115.x 10691148

[123] Yoo D-J. Heterogeneous porous scaffold design for tissue engineering using triply periodic minimal surfaces. International Journal of Precision Engineering and Manufacturing. 2012;13(4):527–37.

[124] Starly B, Lau W, Bradbury T, Sun W. Internal architecture design and freeform fabrication of tissue replacement structures. Computer-Aided Design. 2006;38(2):115–24.

[125] Hieu LC, Bohez E, Vander Sloten J, Phien HN, Vatcharaporn E, Binh PH, et al Design for medical rapid prototyping of cranioplasty implants. Rapid Prototyping Journal. 2003;9(3):175–86. doi: 10.1108/13552540310477481

[126] Devedzic G, Ristic B, Stefanovic M, Cukovic S, Lukovic T. Development of 3D parametric model of human spine and simulator for biomedical engineering education and scoliosis screening. Computer Applications in Engineering Education. 2012;20(3):434–44.

[127] Starly B, Darling A, Gomez C, Nam J, Sun W, Shokoufandeh A, et al, editors. Image based bio-cad modeling and its applications to biomedical and tissue engineering. Proceedings of the ninth ACM symposium on Solid modeling and applications; 2004: Eurographics Association.

[128] Mitsouras D, Liacouras P, Imanzadeh A, Giannopoulos AA, Cai T, Kumamaru KK, et al Medical 3D printing for the radiologist. RadioGraphics. 2015;35(7):1965–88. doi: 10.1148/rg.2015140320 26562233 10.1148/rg.2015140320 PMC4671424

[129] Majstorovic V, Trajanovic M, Vitkovic N, Stojkovic M. Reverse engineering of human bones by using method of anatomical features. CIRP Annals-Manufacturing Technology. 2013;62(1):167–70.

[130] Wang C-S, Wang W-HA, Lin M-C. STL rapid prototyping bio-CAD model for CT medical image segmentation. Computers in Industry. 2010;61(3):187–97.

[131] Li L, Schemenauer N, Peng X, Zeng Y, Gu P. A reverse engineering system for rapid manufacturing of complex objects. Robotics and Computer-Integrated Manufacturing. 2002;18(1):53–67.

[132] Jardini AL, Larosa MA, de Carvalho Zavaglia CA, Bernardes LF, Lambert CS, Kharmandayan P, et al Customised titanium implant fabricated in additive manufacturing for craniomaxillofacial surgery: This paper discusses the design and fabrication of a metallic implant for the reconstruction of a large cranial defect. Virtual and Physical Prototyping. 2014;9(2):115–25.

[133] Rengier F, Mehndiratta A, von Tengg-Kobligk H, Zechmann CM, Unterhinninghofen R, Kauczor H-U, et al 3D printing based on imaging data: review of medical applications. International journal of computer assisted radiology and surgery. 2010;5(4):335–41. doi: 10.1007/s11548-010-0476-x 20467825 10.1007/s11548-010-0476-x

[134] Padala S, Padala V. Fusion of CT and MRI Scanned Medical Images Using Image Processing. Journal of Computer Technology & Applications. 2012;3(3):17–20.

[135] McGurk M, Amis A, Potamianos P, Goodger N. Rapid prototyping techniques for anatomical modelling in medicine. Annals of the Royal College of Surgeons of England. 1997;79(3):169 9196336 PMC2502901

[136] Le C, Okereke M, Nguyen V, Dao V, Soe S, Zlatov N, et al Personalised medical product development: Methods, challenges and opportunities. Romanian Review Precision Mechanics, Optics and Mechatronics. 2011;40:11–20.

[137] Karatas OH, Toy E. Three-dimensional imaging techniques: A literature review. European journal of dentistry. 2014;8(1):132 doi: 10.4103/1305-7456.126269 24966761 10.4103/1305-7456.126269 PMC4054026

[138] Hendee WR, O’Connor MK. Radiation risks of medical imaging: separating fact from fantasy. Radiology. 2012;264(2):312–21. doi: 10.1148/radiol.12112678 22821690 10.1148/radiol.12112678

[139] Tuomi J, Paloheimo K-S, Vehviläinen J, Björkstrand R, Salmi M, Huotilainen E, et al A novel classification and online platform for planning and documentation of medical applications of additive manufacturing. Surgical innovation. 2014;21(6):553–9. doi: 10.1177/1553350614524838 24616012 10.1177/1553350614524838

[140] Marro A, Bandukwala T, Mak W. 3D printing and medical imaging: A review of the methods and applications. Current Problems in Diagnostic Radiology. 2015. 10.1067/j.cpradiol.2015.07.009 26298798

[141] Robiony M, Salvo I, Costa F, Zerman N, Bazzocchi M, Toso F, et al Virtual reality surgical planning for maxillofacial distraction osteogenesis: the role of reverse engineering rapid prototyping and cooperative work. Journal of oral and maxillofacial surgery. 2007;65(6):1198–208. doi: 10.1016/j.joms.2005.12.080 17517306 10.1016/j.joms.2005.12.080

[142] Maji PK, Banerjee AJ, Banerjee PS, Karmakar S. Additive manufacturing in prosthesis development–a case study. Rapid Prototyping Journal. 2014;20(6):480–9.

[143] Arango-Ospina M, Cortés-Rodriguez C, editors. Engineering design and manufacturing of custom craniofacial implants. The 15th International Conference on Biomedical Engineering; 2014: Springer.

[144] Huotilainen E, Jaanimets R, Valášek J, Marcián P, Salmi M, Tuomi J, et al Inaccuracies in additive manufactured medical skull models caused by the DICOM to STL conversion process. Journal of Cranio-Maxillofacial Surgery. 2014;42(5):e259–e65. doi: 10.1016/j.jcms.2013.10.001 24268714 10.1016/j.jcms.2013.10.001

[145] Ma Z, Tavares JMR, Jorge RN, Mascarenhas T. A review of algorithms for medical image segmentation and their applications to the female pelvic cavity. Computer Methods in Biomechanics and Biomedical Engineering. 2010;13(2):235–46. doi: 10.1080/10255840903131878 19657801 10.1080/10255840903131878

[146] Poelert S, Valstar E, Weinans H, Zadpoor AA. Patient-specific finite element modeling of bones. Proceedings of the Institution of Mechanical Engineers, Part H: Journal of Engineering in Medicine. 2013;227(4):464–78. 10.1177/0954411912467884 23637222

[147] Zachow S, Zilske M, Hege H-C. 3D reconstruction of individual anatomy from medical image data: Segmentation and geometry processing: ZIB; 2007.

[148] Singare S, Lian Q, Ping Wang W, Wang J, Liu Y, Li D, et al Rapid prototyping assisted surgery planning and custom implant design. Rapid Prototyping Journal. 2009;15(1):19–23.

[149] Pinto JM, Arrieta C, Andia ME, Uribe S, Ramos-Grez J, Vargas A, et al Sensitivity analysis of geometric errors in additive manufacturing medical models. Medical engineering & physics. 2015;37(3):328–34. 25649961 10.1016/j.medengphy.2015.01.009

[150] Singare S, Dichen L, Bingheng L, Yanpu L, Zhenyu G, Yaxiong L. Design and fabrication of custom mandible titanium tray based on rapid prototyping. Medical engineering & physics. 2004;26(8):671–6. 15471695 10.1016/j.medengphy.2004.06.001

[151] Hiller JD, Lipson H, editors. STL 2.0: a proposal for a universal multi-material additive manufacturing file format. Proceedings of the Solid Freeform Fabrication Symposium; 2009: Citeseer.

[152] Tetsworth K, Block S, Glatt V. Putting 3D modelling and 3D printing into practice: virtual surgery and preoperative planning to reconstruct complex post-traumatic skeletal deformities and defects. SICOT-J. 2017;3. 28220752 10.1051/sicotj/2016043 PMC5319375

[153] Colledani M, Tolio T, Fischer A, Iung B, Lanza G, Schmitt R, et al Design and management of manufacturing systems for production quality. CIRP Annals-Manufacturing Technology. 2014;63(2):773–96.

[154] Konrath J, Saxby D, Killen B, Pizzolato C, Vertullo C, Barrett R, et al Muscle contributions to medial tibiofemoral compartment contact loading following ACL reconstruction using semitendinosus and gracilis tendon grafts. PLoS One. 2017 doi: 10.1371/journal.pone.0176016 28423061 10.1371/journal.pone.0176016 PMC5397063

[155] Galloway F, Worsley P, Stokes M, Nair P, Taylor M. Development of a statistical model of knee kinetics for applications in pre-clinical testing. Journal of Biomechanics. 2012;45(1):191–5. doi: 10.1016/j.jbiomech.2011.09.009 22030123 10.1016/j.jbiomech.2011.09.009

[156] Erdman AG, Keefe DF, Schiestl R. Grand challenge: Applying regulatory science and big data to improve medical device innovation. IEEE Transactions on Biomedical Engineering. 2013;60(3):700–6. doi: 10.1109/TBME.2013.2244600 23380845 10.1109/TBME.2013.2244600

[157] Zhang J, Sorby H, Clement J, Thomas CDL, Hunter P, Nielsen P, et al, editors. The MAP client: User-friendly musculoskeletal modelling workflows2014 2014.

[158] Bachynskyi M, Oulasvirta A, Palmas G, Weinkauf T, editors. Is motion capture-based biomechanical simulation valid for HCI studies?: study and implications. Proceedings of the 32nd annual ACM conference on Human factors in computing systems; 2014: ACM.

[159] Chen J, Rungsiyakull C, Li W, Chen Y, Swain M, Li Q. Multiscale design of surface morphological gradient for osseointegration. Journal of the Mechanical Behavior of Biomedical Materials. 2013;20:387–97. doi: 10.1016/j.jmbbm.2012.08.019 23523124 10.1016/j.jmbbm.2012.08.019

[160] Nguyen V-H, Rosi G, Naili S, Michel A, Raffa M-L, Bosc R, et al Influence of anisotropic bone properties on the biomechanical behavior of the acetabular cup implant: a multiscale finite element study. Computer Methods in Biomechanics and Biomedical Engineering. 2017;20(12):1312–25. doi: 10.1080/10255842.2017.1357703 28768422 10.1080/10255842.2017.1357703

[161] Zhao FH, Vaughan TJ, McNamara LM. Multiscale fluid-structure interaction modelling to determine the mechanical stimulation of bone cells in a tissue engineered scaffold. BIOMECHANICS AND MODELING IN MECHANOBIOLOGY. 2015;14(2):231–43. doi: 10.1007/s10237-014-0599-z 24903125 10.1007/s10237-014-0599-z

[162] Popov I, Onuh S. Reverse engineering of pelvic bone for hip joint replacement. Journal of medical engineering & technology. 2009;33(6):454–9. 19479609 10.1080/03091900902952634

[163] Giannitelli S, Accoto D, Trombetta M, Rainer A. Current trends in the design of scaffolds for computer-aided tissue engineering. Acta biomaterialia. 2014;10(2):580–94. doi: 10.1016/j.actbio.2013.10.024 24184176 10.1016/j.actbio.2013.10.024

[164] Wang X, Xu S, Zhou S, Xu W, Leary M, Choong P, et al Topological design and additive manufacturing of porous metals for bone scaffolds and orthopaedic implants: A review. Biomaterials. 2016;83:127–41. doi: 10.1016/j.biomaterials.2016.01.012 26773669 10.1016/j.biomaterials.2016.01.012

[165] Cheah C, Chua C, Leong K, Chua S. Development of a tissue engineering scaffold structure library for rapid prototyping. Part 1: investigation and classification. The International Journal of Advanced Manufacturing Technology. 2003;21(4):291–301.

[166] An J, Teoh JEM, Suntornnond R, Chua CK. Design and 3D printing of scaffolds and tissues. Engineering. 2015;1(2):261–8.

[167] Chantarapanich N, Puttawibul P, Sucharitpwatskul S, Jeamwatthanachai P, Inglam S, Sitthiseripratip K. Scaffold library for tissue engineering: a geometric evaluation. Computational and mathematical methods in medicine. 2012;2012. 10.1155/2012/407805 PMC3463988 23056147

[168] Wettergreen M, Bucklen B, Starly B, Yuksel E, Sun W, Liebschner M. Creation of a unit block library of architectures for use in assembled scaffold engineering. Computer-Aided Design. 2005;37(11):1141–9.

[169] Yan C, Hao L, Hussein A, Young P. Ti–6Al–4V triply periodic minimal surface structures for bone implants fabricated via selective laser melting. journal of the mechanical behavior of biomedical materials. 2015;51:61–73. doi: 10.1016/j.jmbbm.2015.06.024 26210549 10.1016/j.jmbbm.2015.06.024

[170] Yoo DJ. Porous scaffold design using the distance field and triply periodic minimal surface models. Biomaterials. 2011;32(31):7741–54. doi: 10.1016/j.biomaterials.2011.07.019 21798592 10.1016/j.biomaterials.2011.07.019

[171] Pasko A, Fryazinov O, Vilbrandt T, Fayolle P-A, Adzhiev V. Procedural function-based modelling of volumetric microstructures. Graphical Models. 2011;73(5):165–81.

[172] Yoo D. New paradigms in hierarchical porous scaffold design for tissue engineering. Materials Science and Engineering: C. 2013;33(3):1759–72. 23827634 10.1016/j.msec.2012.12.092

[173] Yoo D-J. Computer-aided porous scaffold design for tissue engineering using triply periodic minimal surfaces. International Journal of Precision Engineering and Manufacturing. 2011;12(1):61–71.

[174] Gabbrielli R, Turner I, Bowen CR, editors. Development of modelling methods for materials to be used as bone substitutes. Key Engineering Materials; 2008: Trans Tech Publ.

[175] Afshar M, Anaraki AP, Montazerian H, Kadkhodapour J. Additive manufacturing and mechanical characterization of graded porosity scaffolds designed based on triply periodic minimal surface architectures. Journal of the Mechanical Behavior of Biomedical Materials. 2016;62:481–94. doi: 10.1016/j.jmbbm.2016.05.027 27281165 10.1016/j.jmbbm.2016.05.027

[176] Almeida HA, Bártolo PJ. Design of tissue engineering scaffolds based on hyperbolic surfaces: Structural numerical evaluation. Medical engineering & physics. 2014;36(8):1033–40. 24935150 10.1016/j.medengphy.2014.05.006

[177] Hollister SJ, Maddox R, Taboas JM. Optimal design and fabrication of scaffolds to mimic tissue properties and satisfy biological constraints. Biomaterials. 2002;23(20):4095–103. 12182311 10.1016/s0142-9612(02)00148-5

[178] Gardan N. Knowledge management for topological optimization integration in additive manufacturing. International Journal of Manufacturing Engineering. 2014;2014.

[179] Huang X, Radman A, Xie Y. Topological design of microstructures of cellular materials for maximum bulk or shear modulus. Computational Materials Science. 2011;50(6):1861–70.

[180] Robbins J, Owen S, Clark B, Voth T. An efficient and scalable approach for generating topologically optimized cellular structures for additive manufacturing. Additive Manufacturing. 2016.

[181] Heynick M, Stotz I, editors. 3D CAD, CAM and rapid prototyping. LAPA Digital Technology Seminar Workshop; 2006.

[182] Atala A, Yoon J. 1 ed. Academic Press, editor2015. 440 p.

[183] Petrovic V, Vicente Haro Gonzalez J, Jorda Ferrando O, Delgado Gordillo J, Ramon Blasco Puchades J, Portoles Grinan L. Additive layered manufacturing: sectors of industrial application shown through case studies. International Journal of Production Research. 2011;49(4):1061–79.

[184] Qian B, Shen Z. Laser sintering of ceramics. Journal of Asian Ceramic Societies. 2013;1(4):315–21.

[185] Kircher R, Christensen A, Wurth K. Electron beam melted (EBM) Co-Cr-Mo alloy for orthopaedic implant applications Austin: Solid Free form Fabrication 2009;436.

[186] Duan B, Wang M. Selective laser sintering and its application in biomedical engineering. MRS bulletin. 2011;36(12):998–1005.

[187] Bogue R. 3D printing: the dawn of a new era in manufacturing? Assembly Automation. 2013;33(4):307–11.

[188] Slotwinski JA, Garboczi EJ, Stutzman PE, Ferraris CF, Watson SS, Peltz MA. Characterization of metal powders used for additive manufacturing. Journal of research of the National Institute of Standards and Technology. 2014;119:460 doi: 10.6028/jres.119.018 26601040 10.6028/jres.119.018 PMC4487284

[189] Zarringhalam H, Hopkinson N, Kamperman N, De Vlieger J. Effects of processing on microstructure and properties of SLS Nylon 12. Materials Science and Engineering: A. 2006;435:172–80.

[190] Butler J. Using selective laser sintering for manufacturing. Assembly Automation. 2011;31(3):212–9.

[191] De Nardo L, Altomare L, Del Curto B, Cigada A, Draghi L. Electrochemical surface modifications of titanium and titanium alloys for biomedical applications Coatings for Biomedical Applications: Woodhead Publishing 2012:106–42.

[192] Kang Y-H, Choi B, Ahn C, Oh S, Lee MS, Jin E-J. Titanium oxide nanotube surface topography and microRNA-488 contribute to modulating osteogenesis. Journal of Nanomaterials. 2014;2014:1.

[193] Tsuchiya H, Macak JM, Müller L, Kunze J, Müller F, Greil P, et al Hydroxyapatite growth on anodic TiO2 nanotubes. Journal of Biomedical Materials Research Part A. 2006;77(3):534–41. doi: 10.1002/jbm.a.30677 16489589 10.1002/jbm.a.30677

[194] Oh S, Daraio C, Chen LH, Pisanic TR, Finones RR, Jin S. Significantly accelerated osteoblast cell growth on aligned TiO2 nanotubes. Journal of Biomedical Materials Research Part A. 2006;78(1):97–103. doi: 10.1002/jbm.a.30722 16602089 10.1002/jbm.a.30722

[195] von Woedtke T, Kramer A. The limits of sterility assurance. GMS Krankenhaushygiene interdisziplinar. 2008;3(3). PMC2831250 20204091

[196] Morrison RJ, Kashlan KN, Flanangan CL, Wright JK, Green GE, Hollister SJ, et al Regulatory Considerations in the Design and Manufacturing of Implantable 3D‐Printed Medical Devices. Clinical and Translational Science. 2015;8(5):594–600. doi: 10.1111/cts.12315 26243449 10.1111/cts.12315 PMC4626249

[197] Lee MH, Arcidiacono JA, Bilek AM, Wille JJ, Hamill CA, Wonnacott KM, et al Considerations for tissue-engineered and regenerative medicine product development prior to clinical trials in the United States. Tissue Engineering Part B: Reviews. 2009;16(1):41–54. 10.1089/ten.TEB.2009.0449 19728784

[198] Di Prima M, Coburn J, Hwang D, Kelly J, Khairuzzaman A, Ricles L. Additively manufactured medical products–the FDA perspective. 3D Printing in Medicine. 2016;2(1):1–6. doi: 10.1186/s41205-016-0005-9 10.1186/s41205-016-0005-9 PMC6027614 29974058

[199] Tang H, Qian M, Liu N, Zhang X, Yang G, Wang J. Effect of powder reuse times on additive manufacturing of Ti-6Al-4V by selective electron beam melting. Jom. 2015;67(3):555–63.

[200] U.S. Department of Health and Human Services. Use of International Standard ISO-10993, Biological Evaluation of Medical Devices Part 1: Evaluation and Testing. Draft Guidance for Industry and Food and Drug Administration Staff In: Services USDoHaH, editor. 2013.

[201] Little TA. Essentials in quality by design. BioProcess International. 2014;12:3.

[202] U.S. Department of Health and Human Services Food and Drug Administration, Center for Biologics Evaluation Research, Center for Drug Evaluation Research. Guidance for industry: Q9 quality risk management U.S. Dept. of Health and Human Services; 2006.

[203] Li J, Zou P. Risk identification and assessment in PPP infrastructure projects using fuzzy analytical hierarchy process and life-cycle methodology. Australasian Journal of Construction Economics and Building. 2012;8(1):34–48. doi: 10.5130/ajceb.v8i1.2996

[204] Rose KH. A guide to the project management body of knowledge Edition F, editor: Project Management Institute; 2013. e1-e p.

[205] Orlandini S, Pinzauti S, Furlanetto S. Application of quality by design to the development of analytical separation methods. Analytical and bioanalytical chemistry. 2013;405(2–3):443–50. doi: 10.1007/s00216-012-6302-2 22941176 10.1007/s00216-012-6302-2

[206] Mauch PD. Quality management: theory and application: CRC press; 2009.

[207] Hillson D, editor Use a risk breakdown structure (RBS) to understand your risks. Proceedings of the project management institute annual seminars & symposium; 2002: San Antonio, Texas, USA.

[208] Liu H-C, Liu L, Liu N. Risk evaluation approaches in failure mode and effects analysis: A literature review. Expert systems with applications. 2013;40(2):828–38.

[209] Stamatis DH. Failure mode and effect analysis: FMEA from theory to execution Second edition ed. Milwaukee, Wisc: ASQ Quality Press; 2003.

[210] Silva DN, De Oliveira MG, Meurer E, Meurer MI, da Silva JVL, Santa-Bárbara A. Dimensional error in selective laser sintering and 3D-printing of models for craniomaxillary anatomy reconstruction. Journal of cranio-Maxillofacial surgery. 2008;36(8):443–9. doi: 10.1016/j.jcms.2008.04.003 18579391 10.1016/j.jcms.2008.04.003

[211] Bibb R, Winder J. A review of the issues surrounding three-dimensional computed tomography for medical modelling using rapid prototyping techniques. Radiography. 2010;16(1):78–83.

[212] Winder J, Bibb R. Medical rapid prototyping technologies: State of the art and current limitations for application in oral and maxillofacial surgery. Journal of Oral and Maxillofacial Surgery. 2005;63(7):1006–15. 16003630 10.1016/j.joms.2005.03.016

[213] Oropallo W, Piegl LA. Ten challenges in 3D printing. Engineering with Computers. 2015:1–14.

[214] Downey J, O'Sullivan D, Nejmen M, Bombinski S, O’Leary P, Raghavendra R, et al Real time monitoring of the CNC process in a production environment- the data collection & analysis phase. Procedia CIRP. 2016;41:920–6. doi: 10.1016/j.procir.2015.12.008

[215] Bi G, Sun C, Gasser A. Study on influential factors for process monitoring and control in laser aided additive manufacturing. Journal of Materials Processing Technology. 2013;213(3):463–8.

[216] Tapia G, Elwany A. A review on process monitoring and control in metal-based additive manufacturing. Journal of Manufacturing Science and Engineering. 2014;136(6):060801.

[217] Ahn D, Kim H, Lee S. Fabrication direction optimization to minimize post-machining in layered manufacturing. International Journal of Machine Tools and Manufacture. 2007;47(3):593–606.

[218] Bikas H, Stavropoulos P, Chryssolouris G. Additive manufacturing methods and modelling approaches: a critical review. The International Journal of Advanced Manufacturing Technology. 2015:1–17.

[219] Colasante C, Sanford Z, Garfein E, Tepper O. Current trends in 3D printing, bioprosthetics, and tissue engineering in plastic and reconstructive surgery. Current Surgery Reports. 2016;4(3):1–14.

[220] Toth T, Hudak R, Zivcak J. Dimensional verification and quality control of implants produced by additive manufacturing. Quality Innovation Prosperity. 2015;19(1):9–21. doi: 10.12776/qip.v19i1.393

[221] Liu H. Numerical analysis of thermal stress and deformation in multi-layer laser metal deposition process: Missouri University of Science and Technology; 2014.

[222] Forging Industry Association. Heat treatment titanium alloys 2015 [cited 2015 November 10th]. Available from: https://www.forging.org/design/374-heat-treating-titanium-alloys.

[223] Liu X, Ma Z, Han D, Lv R, Fang H. Evaluation of uit on titanium alloy residual stress eliminating by ultrasonic residual stress measurement system Rev Adv Mater Sci. 2013;33:266–9.

[224] Craeghs T, Clijsters S, Yasa E, Kruth J-P, editors. Online quality control of selective laser melting. Proceedings of the Solid Freeform Fabrication Symposium, Austin, TX; 2011.

[225] Sun P, Fang ZZ, Xia Y, Zhang Y, Zhou C. A novel method for production of spherical Ti-6Al-4V powder for additive manufacturing. Powder Technology. 2016;301:331–5.

[226] Stoor P, Suomalainen A, Lindqvist C, Mesimäki K, Danielsson D, Westermark A, et al Rapid prototyped patient specific implants for reconstruction of orbital wall defects. Journal of Cranio-Maxillofacial Surgery. 2014;42(8):1644–9. doi: 10.1016/j.jcms.2014.05.006 25139812 10.1016/j.jcms.2014.05.006

[227] Choi J-Y, Choi J-H, Kim N-K, Kim Y, Lee J-K, Kim M-K, et al Analysis of errors in medical rapid prototyping models. International journal of oral and maxillofacial surgery. 2002;31(1):23–32. doi: 10.1054/ijom.2000.0135 11936396 10.1054/ijom.2000.0135

[228] Mallepree T, Bergers D. Accuracy of medical RP models. Rapid Prototyping Journal. 2009;15(5):325–32. doi: 10.1108/13552540910993842

[229] Lamecker H, Kamer L, Wittmers A, Zachow S, Kaup T, Schramm A, et al A method for the three-dimensional statistical shape analysis of the bony orbit. Proc Computer Aided Surgery Around the Head. 2007:94–7.

[230] Chang PS-H, Parker TH, Patrick CW, Miller MJ. The accuracy of stereolithography in planning craniofacial bone replacement. Journal of Craniofacial Surgery. 2003;14(2):164–70. 12621285 10.1097/00001665-200303000-00006

[231] Simonelli M, Tse Y, Tuck C. Effect of the build orientation on the Mechanical Properties and Fracture Modes of SLM Ti–6Al–4V. Materials Science and Engineering: A. 2014;616:1–11.

[232] Ahn S-H, Montero M, Odell D, Roundy S, Wright PK. Anisotropic material properties of fused deposition modeling ABS. Rapid Prototyping Journal. 2002;8(4):248–57.

[233] Coelho PG, Hollister SJ, Flanagan CL, Fernandes PR. Bioresorbable scaffolds for bone tissue engineering: optimal design, fabrication, mechanical testing and scale-size effects analysis. Medical engineering & physics. 2015;37(3):287–96. 25640805 10.1016/j.medengphy.2015.01.004

[234] Leutenecker-Twelsiek B, Klahn C, Meboldt M. Considering part orientation in design for additive manufacturing. Procedia CIRP. 2016;50:408–13.

[235] Hitzler L, Merkel M, Hall W, Öchsner A. A Review of Metal Fabricated with Laser‐and Powder‐Bed Based Additive Manufacturing Techniques: Process, Nomenclature, Materials, Achievable Properties, and its Utilization in the Medical Sector. Advanced Engineering Materials. 2018.

[236] Gerus P, Sartori M, Besier TF, Fregly BJ, Delp SL, Banks SA, et al Subject-specific knee joint geometry improves predictions of medial tibiofemoral contact forces. Journal of biomechanics. 2013;46(16):2778–86. doi: 10.1016/j.jbiomech.2013.09.005 24074941 10.1016/j.jbiomech.2013.09.005 PMC3888900

[237] Saxby DJ, Modenese L, Bryant AL, Gerus P, Killen B, Fortin K, et al Tibiofemoral contact forces during walking, running and sidestepping. Gait & Posture. 2016;49:78–85. 27391249 10.1016/j.gaitpost.2016.06.014

[238] Thambyah A, Pereira BP, Wyss U. Estimation of bone-on-bone contact forces in the tibiofemoral joint during walking. The Knee. 2005;12(5):383–8. doi: 10.1016/j.knee.2004.12.005 16146627 10.1016/j.knee.2004.12.005

[239] Pankaj P. Patient‐specific modelling of bone and bone‐implant systems: the challenges. International Journal for Numerical Methods in Biomedical Engineering. 2013;29(2):233–49. doi: 10.1002/cnm.2536 23281281 10.1002/cnm.2536

[240] Tang Y, Loh HT, Fuh J-Y-H, Wong YS, Lu L, Ning Y, et al Accuracy analysis and improvement for direct laser sintering. 2004.

[241] Guo N, Leu MC. Additive manufacturing: technology, applications and research needs. Frontiers of Mechanical Engineering. 2013;8(3):215–43.

[242] Shirazi SFS, Gharehkhani S, Mehrali M, Yarmand H, Metselaar HSC, Kadri NA, et al A review on powder-based additive manufacturing for tissue engineering: selective laser sintering and inkjet 3D printing. Science and Technology of Advanced Materials. 2016. 10.1088/1468-6996/16/3/033502 PMC5099820 27877783

[243] Tontowi AE, Childs T. Density prediction of crystalline polymer sintered parts at various powder bed temperatures. Rapid Prototyping Journal. 2001;7(3):180–4.

[244] Bordat C, McCullouch B, Sinha K. An analysis of cost overruns and time delays of INDOT projects. Joint Transportation Research Program. 2004:11.

[245] Wegner A, Witt G editors. Process monitoring in laser sintering using thermal imaging SFF Symposium, Austin, Texas, USA; 2011.

[246] Frazier WE. Metal additive manufacturing: a review. Journal of Materials Engineering and Performance. 2014;23(6):1917–28.

[247] Chia HN, Wu BM. Recent advances in 3D printing of biomaterials. Journal of biological engineering. 2015;9(1):1. 25866560 10.1186/s13036-015-0001-4 PMC4392469

[248] Amini AR, Laurencin CT, Nukavarapu SP. Bone tissue engineering: recent advances and challenges. Critical Reviews™ in Biomedical Engineering. 2012;40(5). 10.1615/critrevbiomedeng.v40.i5.10 PMC3766369 23339648

[249] Junker R, Dimakis A, Thoneick M, Jansen JA. Effects of implant surface coatings and composition on bone integration: a systematic review. Clinical Oral Implants Research. 2009;20(s4):185–206. doi: 10.1111/j.1600-0501.2009.01777.x 19663965 10.1111/j.1600-0501.2009.01777.x

[250] Park JH, Olivares-Navarrete R, Baier RE, Meyer AE, Tannenbaum R, Boyan BD, et al Effect of cleaning and sterilization on titanium implant surface properties and cellular response. Acta biomaterialia. 2012;8(5):1966–75. doi: 10.1016/j.actbio.2011.11.026 22154860 10.1016/j.actbio.2011.11.026 PMC3618465

[251] Bozic KJ, Kurtz SM, Lau E, Ong K, Chiu V, Vail TP, et al The epidemiology of revision total knee arthroplasty in the United States. Clinical Orthopaedics and Related Research®. 2010;468(1):45–51. doi: 10.1007/s11999-009-0945-0 19554385 10.1007/s11999-009-0945-0 PMC2795838

[252] Kummer KM, Taylor EN, Durmas NG, Tarquinio KM, Ercan B, Webster TJ. Effects of different sterilization techniques and varying anodized TiO2 nanotube dimensions on bacteria growth. Journal of Biomedical Materials Research Part B: Applied Biomaterials. 2013;101B(5):677–88. doi: 10.1002/jbm.b.32870 23359494 10.1002/jbm.b.32870

[253] Kruth J-P, Bartscher M, Carmignato S, Schmitt R, De Chiffre L, Weckenmann A. Computed tomography for dimensional metrology. CIRP Annals-Manufacturing Technology. 2011;60(2):821–42.

[254] Bell C. 3D printer maintenance: preventive and corrective tasks Maintaining and Troubleshooting Your 3D Printer. Berkeley, CA: Apress; 2014 p. 327–67.

[255] Bell C. Calibrating the Printer Maintaining and Troubleshooting Your 3D Printer. Berkeley, CA: Apress; 2014 p. 175–204.

[256] Binder WJ, Dhir K. Internet access to advanced 3-dimensional software for the prototyping and design of complex and precise custom mandibular implants. The American Journal of Cosmetic Surgery. 2016:0748806816648677.

[257] Negi S, Dhiman S, Kumar Sharma R. Basics and applications of rapid prototyping medical models. Rapid Prototyping Journal. 2014;20(3):256–67. doi: 10.1108/rpj-07-2012-0065

[258] Hemmert M. The influence of institutional factors on the technology acquisition performance of high-tech firms: survey results from Germany and Japan. Research Policy. 2004;33(6–7):1019–39.

[259] Amid A, Ghodsypour SH, O’Brien C. Fuzzy multiobjective linear model for supplier selection in a supply chain. International Journal of Production Economics. 2006;104(2):394–407. http://dx.doi.org/10.1016/j.ijpe.2005.04.012.

[260] Sharples S, Martin J, Lang A, Craven M, O’Neill S, Barnett J. Medical device design in context: A model of user–device interaction and consequences. Displays. 2012;33(4):221–32.

[261] Shaw B. The role of the interaction between the user and the manufacturer in medical equipment innovation. R&D Management. 1985;15(4):283–92.

[262] Omar M, Zeller AN, Gellrich NC, Rana M, Krettek C, Liodakis E. Application of a customized 3D printed reduction aid after external fixation of the femur and tibia. The International Journal of Medical Robotics and Computer Assisted Surgery. 2017. 10.1002/rcs.1803 28120475

[263] Walker JM. Additive manufacturing towards the realization of porous and stiffness-tailored NiTi implants: University of Toledo 2014.

[264] Enab TA, Bondok NE. Material selection in the design of the tibia tray component of cemented artificial knee using finite element method. Materials & Design. 2013;44:454–60.

