# Supplementary material for: Application of quality by design for 3D printed bone prostheses and scaffolds
Source: PLoS One. 2018 Apr 12;13(4):e0195291. doi: 10.1371/journal.pone.0195291 (PMC5896968; doi:10.1371/journal.pone.0195291)
Supplement: S1 File — (PDF) [file pone.0195291.s001.pdf]

Supplementary material S1: Prior QbD study descriptive statistics

Application of quality by design for 3D printed bone prostheses and scaffolds

Daniel. Martinez-Marquez[1], Ali. Mirnajafizadeh[2], Christopher P. Carty [3,4,5], Rodney A. Stewart[1*]

[1] School of Engineering, Griffith University, Gold Coast, Queensland, Australia

[2] Molecular Cell Biomechanics Laboratory, University of Berkeley, Berkeley, California, USA

[3] School of Allied Health Sciences and Innovations in Health Technology, Menzies Health Institute Queensland, Griffith University, Gold Coast, Queensland, Australia

[4] Centre for Musculoskeletal Research, Menzies Health Institute Queensland, Griffith University, Gold Coast, Queensland, Australia

[5] Queensland Children's Gait Laboratory, Queensland Paediatric Rehabilitation Service, Children's Health Queensland Hospital and Health Service, Brisbane, Queensland, Australia

* Rodney A. Stewart

Email: r.stewart@griffith.edu.au (RAS)

Systematic search for application and outcomes of the Quality by Design Approach

1. Materials and methods

Since the introduction of QbD in 2004 in the pharmaceutical field, it has been widely used in different pharmaceutical fields to enhance process understanding, predict and optimize pharmaceutical formulations, optimize processes, and reduce variability in production to ensure robust manufacturing methods. However, to understand in more detail the potential benefits that QbD approach can provide for the development of custom 3D printed bone prostheses and scaffold, and how it have been used in different research fields we analysed 30 different studies that applied this approach.

2. Data collection

A systematic search of Science Direct, was conducted on 2nd August 2017 according to the Prisma statement [1]. Search related term was "*quality by design*".

3. Study selection

Selected studies were limited to the following inclusion criteria: (1) peer-reviewed papers with full-text; (2) empirical studies showing evidence of the applicability of Quality by Design approach; (3) published in English language; (4) assess the first ten pages of the search results; (5) sort the search results by relevance.

4. Data extraction and analysis

Full-text screening was independently performed by the authors. Any discrepancy between the two reviewers was resolved by a consensus meeting. The articles were thoroughly reviewed and analysed to extract the following information: Application of QbD, QbD implemented steps;

QBD tools used; Key output/conclusion. Moreover, the articles' objective/key output was classified in four different categories: Process understanding (PU); Prediction and optimization (PO); Reduction of experimental runs (RER); Development of robust manufacturing methods (DRM). Furthermore, the total number of experiments that each study performed was gathered, including the calculation of the number of experiments if they had used one variable at the time, for more detailed information refer to S4_Database file. The summary of extracted information mentioned above can be seen in Table 1.

Table 1. Data extracted from the reviewed studies

Study Code	QbD Application context	QbD implemented steps	Objective / Key output	Objective category	No of experiments with one variable at the time	No of experiments with DOE	Ref
S1	Pharmaceutical development of an intranasal nanosized formulation	QTPP, CQA, CPP, RA, DS	*"The practical research **justified the theoretical prediction**. Their **experimental results reflected and correlated with the RA results**." "The study confirmed that a QbD-based experimental design and **RA can help to reduce the practical aspects of the early development research** in pharmaceutical technology by **predicting the parameters that most strongly influence the final quality.**" "The results of the practical research justified the theoretical prediction. This method can improve pharmaceutical nano-developments by **achieving shorter development time, lower cost, saving human resource efforts and more effective target-orientation**. It makes possible focusing the resources on the selected parameters and area during the practical product development"*	PU,PO	L:3<br>F:3<br>Total:27	Total:27	[2]
S2	Development of a starch-based nanoparticulate carrier system (StNC) for topical delivery of lipophilic bioactive molecules	QTPP, CQA, CPP, RA, DS	*"The manufacturing process and the **formula of the StNC were simultaneously optimized**." "The production of StNC **was optimized in terms of composition and process** using a QbD approach, enabling to successfully develop StNC with an optimized nanometric particle size and acceptable stability."*	PO, DRM	L:3<br>F:3<br>Total:27	Total:17	[3]
S3	Design of naproxen enteric-coated pellets (NAP-ECPs) by fluid-bed coating	CQA, CPP, CMA, RA, DS	*"Due to the **extreme coincidence of the predicted value** generated by model with the observed value, **the accuracy and robustness of the model were confirmed**." "It could be concluded that a promising **NAP-ECPs was successfully designed using** QbD approach in a laboratory scale."*	PO,DRM	L:2<br>F:8<br>Total:256<br>L:3<br>F:3<br>Total:27	Total:12<br>Total:15	[4]
S4	Development of lyophilized liposomes with simvastatin	CQA, DS	*"The current work brings an important innovative contribution in the field of lyophilized liposomes, through the successful application of the QbD approach in the development of lyo-LCL-SIM. In this manner, **the influence of both formulation and process parameters on the CQAs of lyo-LCL-SIM was determined through the use of DoE**." "This modern, scientifically based approach of pharmaceutical development, **enabled us to determine the design space** for lyo-LCL-SIM, in which the established quality requirements of the product are met, provided that the risk factors vary within the established limits. We could conclude that QbD is a **useful, time-effective strategy** for the development of lyophilized liposome's **having controlled, predictable quality.**"*	PU	L:3<br>F:5<br>Total:243	Total:21	[5]
S5	Improve the manufacturing process understanding of botanical drug products	PFD, CPP, DS	*"This case study demonstrated that QbD is a powerful tool in **improving the knowledge** of the relationships between potentially high-risk factors and the performance of ethanol precipitation." "The process characterization studies and the design space provide opportunities for establishing control strategies of ethanol precipitation. It is envisioned that developing manufacturing process of botanical drug products under the framework of QbD **not only***	PU	L:3<br>F:3<br>Total:27	Total:16	[6]

			enhances process understanding*, but also facilitates the optimization and control of process to produce the products with desired quality.”*				
S6	Drug substance manufacturing process of an Fc fusion protein	CQA, CPP, RA, DS, DICS	*“Finally, the control strategy was devised to* ***ensure consistent manufacturing*** *of drug substance meeting pre-defined quality targets. The concept of the global multi-step Design Space was introduced:* ***predictive models of product quality developed for each individual step were linked to give operational limits for the entire manufacturing process****.” “The global multi-step Design Space allowed the development of an adaptive strategy where the QbD goal of increased manufacturing flexibility was realised:* ***final product of the desired quality was obtained through adjustment of operating parameters in*** *response to the variability in input material characteristics.”*	PO, DRM	L:<br>F:<br>Total: NA	Total: NA	[7]
S7	Development and Evaluation of Paclitaxel Nanoparticles	CQA, RA, DS	*“In this study, the effects of formulation and process variables were evaluated.” “With the fundamental understanding of the variables and the process, the outcomes of this study can be applied to nanoparticulate drug delivery systems encapsulating active substances other than paclitaxel.”*	PU	L:2<br>F:8<br>Total:256<br>L:3<br>F:3<br>Total:27	Total:12<br>Total:15	[8]
S8	Development and characterisation of efavirenz (EFZ) loaded solid lipid nanoparticles (SLN)	QTPP, CQA, CPP, RA, DS	*“This study suggests the importance of implementation of QbD* ***approach for identifying critical process and formulation parameter*** *in order to develop nano based pharmaceutical product.”*	PU	L:3<br>F:2<br>Total:18	Total:18	[9]
S9	Understand and define optimal parameters for preparation of small interfering RNA (siRNA) loaded PLGA nanoparticles by the double emulsion solvent evaporation method and characterize their properties.	CPP, DS	*“These results* ***enable careful understanding and definition of optimal process parameters*** *for preparation of PLGA nanoparticles encapsulating high amounts of siRNA with immediate and long-term sustained release properties.” “****The measured encapsulation efficiency ranged from 2.01% to as much as 51.18%. (there is a large variation****)”“This study demonstrates that it is possible to increase the encapsulation efficiency without the use of cationic* ***co-excipients to more than 60–70%*** *of biologically active siRNA by the choice of optimized formulation parameters without compromising the particle size and the negative particle zeta potential.”*	PU, PO, DRM	L:3<br>F:5<br>Total:243	Total:18	[10]
S10	The present study is to demonstrate the useful- ness of the QbD approach within the framework of a real example illustrating an issue with unexpected impurities appearing during the last stage of a long-term stability study of a controlled released drug formulation (under confidential agreement)	CQA, PFD, CPP, DS, DICS	*“****The developed method was also subsequently used in routine analysis, demonstrating the usefulness*** *of the QbD approach for the development of a stability-indicating method. The QbD methodology followed in this paper could be advantageously applied to the development and improvement of any stability-indicating method.”*	PU, PO, DRM	L:<br>F:<br>Total:	Total:	[11]
S11	Product and process design for brivanib alaninate filme-coated tablets	CQA, PFD, CPP, RA, DS, DICS	*“Using a risk-based approach, the strategy for* ***development entailed identification*** *of product critical quality attributes (CQAs), assessment of risks to the CQAs, and performing experiments to* ***understand and mitigate*** *identified risks” “This demonstration of QbD principles to integrated and holistic drug product development highlights the value of mechanistically driven experiments in* ***building product and process robustness*** *while addressing key risks to the patient, manufacturing plant, and product development.”*	PU, PO, DRM	L:3<br>F:4<br>Total:81	Total:20	[12]
S12	Development, optimization of a in situ gelling microemulsion of Lorazepam via intranasal route	DS	*“Ex vivo diffusion studies revealed significantly higher release for MEG compared to microemulsion and drug solution. MEG showed higher flux and permeation across goat nasal mucosa.” “According to the study, it could be concluded that formulation would successfully provide the rapid onset of action, and decrease the mucociliary clearance due to formation of in situ gelling mucoadhesive system.” “****MEG has shown higher brain targeting, higher drug release*** *from formulation and no evidence on nasal ciliotoxicity.* ***This may help in decreasing dose and frequency of administration*** *of drug and may*	PO	L:3<br>F:2<br>Total:27	Total:9	[13]

			possibly **maximize therapeutic benefits and may also reduce cost of therapy**.”				
S13	Improvement of Tablet Coating Uniformity	CPP, DS	“This systematic application of the QbD-based method resulted in an **enhanced process understanding** and **reducing the coating variability by more than half**.” “These results reported here demonstrate that the new coating process **optimized** by this QbD study **was robust and produced consistent results**.”	PU, PO, DRM	L:<br>F:<br>Total: NA	Total: NA	[14]
S14	Understand the impact of formulation and process variables on the quality of oral disintegrating films (ODF)	CQA,CPP, CMA, DS	“All the DoE formulations showed rapid drug dissolution wherein **almost 90% of the drug dissolved in 3–6 min** (Fig. 4). In contrast, ODF reported in literature required **a minimum of 10– 40 mins for complete dissolution**.” “It was concluded that Quality by Design (QbD) study, employed in the present investigation, **helped in optimizing product and process variables** impacting the CQA of LMT ODF product.”	PU, PO, DRM	L:3<br>F:5<br>Total:243	Total:14	[15]
S15	Maximize the understanding of the unit operation of microfluidization, for the preparation of nanosuspensions using indomethacin as a model drug.	CQA,DS	“This quality by design approach facilitated the elucidation of **various two-way interactions between independent variables which are impossible to detect with the conventional one factor at a time methodology**.” “This research **highlights the level of understanding** that can be **accomplished** through a well-designed study based on the philosophy of QbD.”	PU	L:3<br>F:5<br>Total:243	Total:22	[16]
S16	To facilitate an in-depth process understanding, and offer opportunities for developing control strategies to ensure product quality, a combination of experimental design, optimization and multivariate techniques was integrated into the process development of a drug product	CQA, PFD, RA, DS	“It was **demonstrated that the DOE effect/response surface analysis was a powerful tool in studying the effects of selected factors** (water amount, wet massing time and lubrication time) on response variables, and establishing design space **to ensure the desired manufacturability**—tablet blend flow.” “It is clear that DOE effect/response surface analysis and multivariate data analysis are complementary tools for pharmaceutical product and process development. **The level of understanding would not be achieved with either approach alone.**”	PU	L:5<br>F:3<br>Total:125	Total:13	[17]
S17	Formulation and Process Design Space for Roller-Compacted Ciprofloxacin Hydrochloride Immediate-Release Tablets	QTPP, CQA, CPP, DS	“In summary, scientific rationale and quality risk management analysis were used to **successfully and efficiently determine the CQAs coming from the formulations and the manufacturing processes**.”	PU	L:2<br>F:11<br>Total:2048	Total:12	[18]
S18	Retrospective Quality by Design (rQbD) applied to the optimization of orodispersible films	CQA, CPP, DS	“The rQbD approach followed in this work contributed to fill a gap on literature by extending the scientific understanding of the influence of CPPs on the stability of drug release profile and to define a design space for this novel dosage form” “This work shows that it is possible to apply rQbD to achieve a greater understanding of the manufacturing process of ODFs and to define a proper design space”	PU, PO	L:<br>F:<br>Total:	Total:	[19]
S19	Development of dispersible tablets	QTPP, CQA, PFD, CPP, DS, RA, DICS	“The better process understanding and control are vital to minimize the product waste due to manufacturing failure and produce product of desired quality with reduced end product testing. These objectives were accomplished by identifying process variables for preparing robust diclofenac dispersible tablets, measuring and monitoring them as provided in process analytical technology” “Consequently, this study marked a possibility of a major shift from traditional QbT approach to enhance the manufacturers’ confidence in their products as well as to relieve the FDA work load significantly as quality is built in the system”	PU, PO, DRM	L:2<br>F:2<br>Total:4<br>L:2<br>F:3<br>Total:8	Total:4<br>Total:8	[20]
S20	Development of Ohydrophilic interaction liquid chromatographic (HILIC) method for the analysis of bilastine and its degradation impurities	CQA, CPP	This study presents a step by step development of HILIC method for the analysis of bilastine and its degradation impurities following Analytical Quality by Design approach. Taking into account the complexity of HILIC technique and that bilastine is investigated for the first time, systematic selection of factors and their ranges was carried out. After that, following AQbD roles, **method’s design space was established and optimal and robust chromatographic conditions were defined**. Finally, the method was **fully validated and its applicability for the real samples analysis was confirmed.**	PU, PO, DRM	L:3<br>F:3<br>Total:27<br>L:3<br>F:7<br>Total:2187	Total:15<br>Total:11	[21]

S21	Pharmaceutical development of enoxaparin sodium microspheres for colon-specific delivery.	QTPP,CQA, CPP, DS	*The in vitro **release profile of enoxaparin sodium was close to the ideal one**, therefore the **system was successfully designed** using QbD approach. "The QbD approach was used to achieve a design space with the desired QTPP as well as to determine the **optimum formulation within the design spac**e. The optimum formulation was characterized and found to possess very good characteristics: small size, high drug entrapment and gradual release of the drug from the microspheres, appropriate to a colonic drug delivery system. Consequently, this study demonstrated the practical gain of the QbD approach in pharmaceutical drug development, in this case the rapid development of colon-specific delivery microspheres with the desired QTPP."*	PU, PO	L:3<br>F:3<br>Total:27	Total:17	[22]
S22	Development of nanostructured lipid carriers containing salicyclic acid for dermal use	QTPP, CQA, CPP, CMA, RA,	*"Three highly critical quality attributes (CQAs) for NLC SA were identified, namely particle size, particle size distribution and aggregation. Five attributes of medium influence were identified, including dissolution rate, dissolution efficiency, pH, lipid solubility of the active pharmaceutical ingredient (API) and entrapment efficiency." "Based on our experiments, an optimal formulation can be obtained" "The present paper describes a **successfully optimized formula** of nanostructured lipid carriers containing salicylic acid, prepared by applying the QbD concept on the development process." "Based on these results, a promising salicylic acid-loaded NLC formulation could be developed which can function as a potential dermal drug delivery system in the treatment of acne, psoriasis and eczema"*	PU, PO	L:2<br>F:3<br>Total:8	Total:8	[23]
S23	Development of aceclofenac loaded-nano structured lipid carriers (NLCs)	QTPP, CQA, CMA, RA, DS	*ACE-NLCs **were successfully formulated**, characterized, and optimized by using QbD approach for topical drug delivery.*	PU, PO	L:2<br>F:7<br>Total:128<br>L:3<br>F:3<br>Total:27	Total:8<br>Total:17	[24]
S24	Development of pellets for oral lysozyme delivery	CQA, CPP, RA	*"Use of the QbD approach during the formulation of biotechno-logically produced APIs is essential. The high variability and sensitivity of these molecules demand **a better understanding of the effects** of the CPPs on the CQAs of the products. The present results **partially confirmed** the original assumptions of the risk assessment"*	PU	L:2<br>F:6<br>Total:64	Total:64	[25]
S25	Design of 5-fluorouracil loaded lipid nanoparticles by the W/O/W double emulsion	QTPP, CQA, CPP, DS, DICS	*"The optimal formulations recommended by the models were prepared and the critical quality attributes belonging to those formulations were assigned. The experimental **results remained within the design space limit value**s. Consequently, **optimal formulations with the CQA determined to achieve the QTPP were successfully obtained** within the design space by following the QbD steps." "These formulations were examined experimentally and found to successfully reside within the design space. ANN programs are quite advantageous for the industry because they provide detailed results and **prevent unnecessary studies which cause a loss of time and money.** Consequently; the emphasis of this study is to demonstrate the practical gain of the QbD approach in pharmaceutical drug development. It can be concluded that optimum 5-FU loaded lipid nanoparticle formulation could be achieved by QbD."*	PU, PO	L:<br>F:<br>Total:	Total:	[26]
S26	A multivariate formulation robustness study was performed for a selected formulation of a monoclonal antibody to demonstrate acceptable quality at the target composition as well as at the edges of the allowable composition ranges and fulfilment of the end-of-shelf-life stability requirements of 36 months at the intended storage temperature.	CQA, DS	*"In our study, first, we predefined acceptance limits for the CQAs tested in formulation robustness studies, based on QbD principles and in alignment with the project specific overall control strategy. Second, an MLR model was calculated. In the case that the MLR model is statistically not significant, we can deduce that the formulation is robust within the formulation composition ranges." "Based on this case study, **we propose that a formulation can be claimed as "robust" if all drug substance and drug product critical quality attributes remain within their respective end-of-shelf-life** critical quality attribute acceptance criteria throughout the entire claimed formulation composition range."*	PU, PO	L:2<br>F:3<br>Extra experiments : 3<br>Total:11	Total:11	[27]
S27	Design space definition of a blending unit operation	CQA, CPP, CMA, RA, DS	*"We show that a structured and **systematic quality risk management approach** within the QbD-framework **significantly contributes** to an **efficient development process**. This is achieved by identifying those quality attributes and input parameters that are crucial for further investigations. By subsequent linking of the safety and efficacy of the product with the identified quality attributes, a starting point for*	PU	L:<br>F:<br>Total:	Total:	[28]

			process development can be defined" "This information is of crucial importance in the development phase of the blending unit."					
S28	Development, optimization, and evaluation of Carbopol 940 topical gel containing aceclofenac-crospovidone (1:4) solid dispersion	CQA, DE	*"The selected QbD strategy allowed an **efficient selection of the best formulation composition and of the most suitable experimental conditions in the shortest time and with the minimum number of experiments**. The best formulation was studied for in-vivo pharmaco dynamic performance in carrageen-induced rat paw oedema model and was compared with marketed gel formulation." "Carbopol 940 topical gel containing aceclofenac-crospovidone solid dispersion was **successfully developed and optimized** by QbD approach based on 2^3 factorial design."*	PU, PO	L:2<br>F:3<br>Total:8	Total:8	[29]
S29	Understand the spray drying process of insulin intended for pulmonary administration	CPP	*"Prediction equations were obtained for all dependent variables including both interaction and quadratic terms." "The results indicate that formulation parameters are at least as important as process parameters when spray drying proteins. In particular, parameters affecting the critical concentration are important when designing a proper process for spray drying proteins. Design of experiments and multivariate data analysis proved to be useful tools for QBD and **were able to identify important parameters and variable correlations**."*	PU,PO	L:3<br>F:5<br>Total:125	Total:31	[30]
S30	Development of a stability indicating UHPLC method for ebastine in the API and pharmaceutical formulations	CPP, RA, DS, DICS	*"A verification study demonstrated that the **established model for Design Space is accurate with a relative error of prediction of only 0.6%.**" A Design Space – a volume in which the **method is robust is defined and visualized**. Also, the predicted retention times and **resolution values are in excellent agreement with experimental values**. The method was fully validated in compliance with ICH guidelines and a robustness study was performed by varying six chromatographic parameters. **The analysis time is less than 4 min, which is an impressive 40-fold increase in productivity** in comparison to the method published in the E.P. monograph and allows purity testing of more than 360 samples per day. "Moreover, a single run takes 160 min and is therefore not acceptable for routine analysis."*	PU, PO, DRM	L:<br>F:<br>Total:	Total:	[31]

5. Results

A total of 30 studies were statistically and qualitative analysed to identify: the most implemented QbD steps, the reasons to use QbD system in research, what positive results and drawbacks were encountered, and what was the total reduction of experiments obtained by using QbD system. According to our results, QbD has been widely used in different pharmaceutical fields for several purposes, such as formulation and process design [27], improvement of drug manufacturing [38-40], and development of nano based pharmaceutical products [41-43]. Moreover, it was found that the most implemented QbD steps in the reviewed studies are: identification of critical process parameters and material attributes (CPP/CMA), design space (DS), and identification of critical quality attributes (CQA), with 93%, 87%, and 77% respectively, as shown in Figure 1c. Whereas the least QbD steps used were: development of strategies for product lifecycle management and continuous improvement (PLMCI), process flow diagram (PFD), and design and implement a control strategy (DICS), with 0%, 23%, and 17% respectively.

Furthermore, in this analysis it was identified that QbD was mainly used to achieve four different objectives: process understanding (PU), prediction and optimization (PO), reduction of

experimental runs (RER), and development of robust manufacturing methods (DRM), as shown in Figure 1a.

5.1. Process understanding (PU)

First, the majority of the studies (86%) used QbD to enhance process understanding (PU). Where the identification of CQA and CPP/CMA were the main steps of the QbD approach used for this purpose, representing 77% and 93% respectively (Figure 1c). For example, Verma et al. [1] used QbD to gain a comprehensive understanding of the preparation and processing of nanosuspensions via microfluidization by identifying various two-way interactions between independent variables which were impossible to detect using other methods. Similarly, Zhang et al. [2] used QbD to improve the manufacturing process understanding of botanical drug products. In this study they identified the critical factors that can affect the performance of ethanol precipitation process, to then develop a potential design space of ethanol precipitation operation. According to Zhang et al. [2] *"QbD is a powerful tool in improving the knowledge of the relationships between potentially high-risk factors and the performance of ethanol precipitation…"*.

5.2. Prediction and optimization (PO) and reduction of experimental runs (RER)

Prediction and optimization of pharmaceutical formulations (PO), and reduction of experimental runs (RER) were the second and third reasons of using QbD with 56% and 53% respectively, as shown in Figure 1a. A total of 16 studies out of 30, aimed for the reduction of experimental runs (Figure 1b). Incredibly 50% of these studies reduced their experimental runs by more than 90%. In average the reduction of experimental runs was 78%. In regards to optimization of pharmaceutical formulations, Mazumder et al. [3] optimized the formulation of an oral disintegrating film to reduce its dissolution time from 10-40 mins to 3-6 min, representing and improvement of 85%. In another study, Schmidt and Molnár [4] reduced the purity testing of non-

sedating antihistamine using ultra high performance liquid chromatographic (UHPLC) method from 160 min to 4 min, representing an impressive 40-fold increase in productivity.

5.3. Development of robust manufacturing methods (DRM)

The fourth reason identified for implementing of QbD was for the development of robust manufacturing methods (DRM) by reducing variability in production. Just 36% of these studies used QbD for this purpose (Figure 1a), and 23% implemented the 7th step of the QbD approach which aims to design and implement a control strategy (DICS), as shown in Figure 1c. A good example of variability reduction with QbD is Cun et al. [5] study, where they were able to increase the encapsulation efficiency of Poly(DL-lactide-co-glycolide acid) PLGA nanoparticles from 2.01% to 60%, and reduce the variation efficiency from 49.17% to just 10%. Also Dubey et al. [6] was able to improve their production variability. In their study they reduced the tablet coating variability by more than 50%, and concluded: "*These results reported here demonstrate that the new coating process optimized by this QbD study was robust and produced consistent results*".

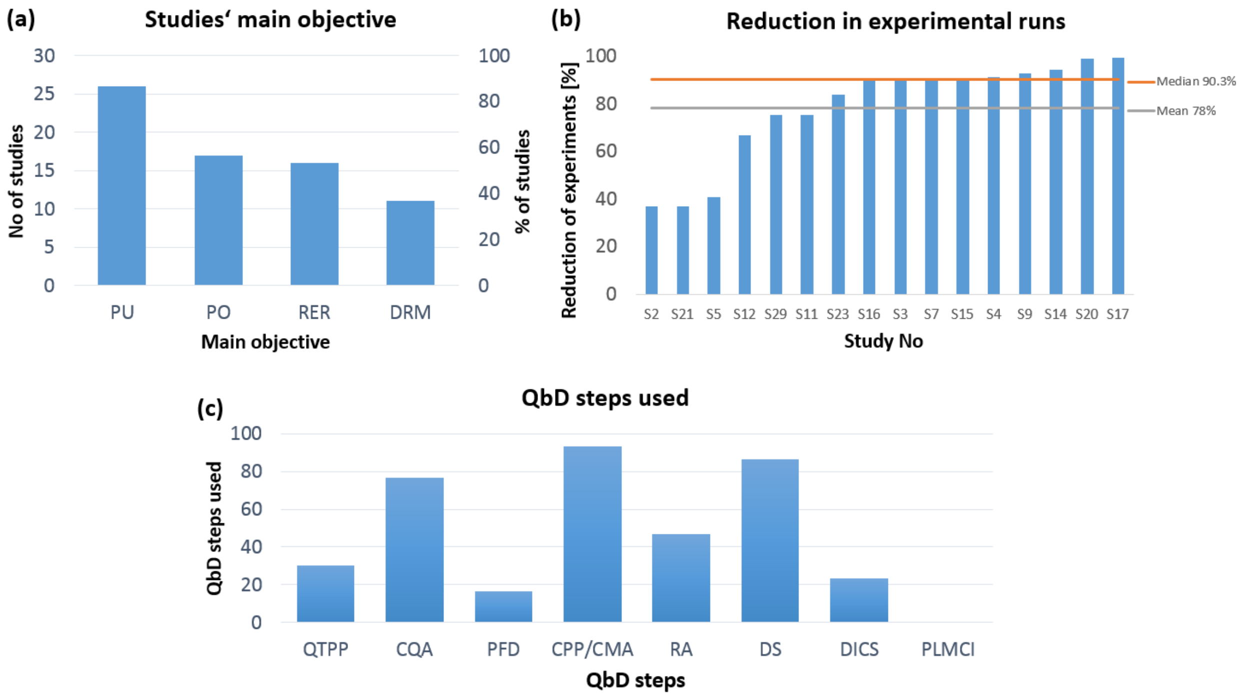

Figure 1. **(a)** Studies' main objective, *PU: Process understanding, PO: Prediction and optimization, RER: Reduction of experimental runs, DRM: development of robust manufacturing*; **(b)** Percentage of reduction of experimental runs per paper; **(c)** QbD steps implemented in the reviewed studies.

6. Conclusion

In summary, the results suggests that the majority of the reviewed studies presented low levels of maturity in regards to the product development life cycle. The reason is that most of research studies were not aimed to reach commercial stages, therefore the 7th (DICS) and 8th (PLMCI) QbD steps were be perceived irrelevant for most of these research studies. Additionally, it has to be noted that the QbD system was developed to assist and accelerate the product development process in pharmaceutical companies, therefore is not a surprise that all the steps of the QbD system were not implemented in any study.

Nevertheless, it can be said the all these studies obtained positive results from the implementation of QbD. Moreover, despite the fact that just a few studies aimed for DRM and implement the DICS step, they achieved remarkable results. Furthermore, most studies had a dramatic reduction of experimental runs and process variability, which can be translated in lower cost and improved quality. The implementation of QbD can provide similar benefits to different kind of process and products regardless the industry to which they belong. Therefore, QbD is a powerful system that can assist the development of 3D printed bone implants and scaffolds to provide better products and process understanding, ensure robust manufacturing, and minimize development risks and cost.

References

1. Liberati A, Altman DG, Tetzlaff J, Mulrow C, Gøtzsche PC, Ioannidis JP, et al. The PRISMA statement for reporting systematic reviews and meta-analyses of studies that evaluate health care interventions: explanation and elaboration. Annals of internal medicine. 2009;151(4):W-65-W-94.
2. Pallagi E, Ambrus R, Szabó-Révész P, Csóka I. Adaptation of the quality by design concept in early pharmaceutical development of an intranasal nanosized formulation. International journal of pharmaceutics. 2015;491(1):384-92.
3. Marto J, Gouveia L, Gonçalves L, Gaspar D, Pinto P, Carvalho F, et al. A quality by design (QbD) approach on starch-based nanocapsules: a promising platform for topical drug delivery. Colloids and Surfaces B: Biointerfaces. 2016;143:177-85.

4. Kan S, Lu J, Liu J, Wang J, Zhao Y. A quality by design (QbD) case study on enteric-coated pellets: Screening of critical variables and establishment of design space at laboratory scale. asian journal of pharmaceutical sciences. 2014;9(5):268-78.
5. Porfire A, Muntean DM, Rus L, Sylvester B, Tomuţă I. A quality by design approach for the development of lyophilized liposomes with simvastatin. Saudi Pharmaceutical Journal. 2017.
6. Zhang L, Yan B, Gong X, Lawrence XY, Qu H. Application of quality by design to the process development of botanical drug products: a case study. Aaps Pharmscitech. 2013;14(1):277-86.
7. Eon-duval A, Valax P, Solacroup T, Broly H, Gleixner R, Strat CL, et al. Application of the quality by design approach to the drug substance manufacturing process of an Fc fusion protein: Towards a global multi-step design space. Journal of pharmaceutical sciences. 2012;101(10):3604-18.
8. Yerlikaya F, Ozgen A, Vural I, Guven O, Karaagaoglu E, Khan MA, et al. Development and Evaluation of Paclitaxel Nanoparticles Using a Quality-by-Design Approach. Journal of pharmaceutical sciences. 2013;102(10):3748-61.
9. Raina H, Kaur S, Jindal AB. Development of efavirenz loaded solid lipid nanoparticles: Risk assessment, quality-by-design (QbD) based optimisation and physicochemical characterisation. Journal of Drug Delivery Science and Technology. 2017;39:180-91.
10. Cun D, Jensen DK, Maltesen MJ, Bunker M, Whiteside P, Scurr D, et al. High loading efficiency and sustained release of siRNA encapsulated in PLGA nanoparticles: quality by design optimization and characterization. European Journal of Pharmaceutics and Biopharmaceutics. 2011;77(1):26-35.
11. Hubert C, Lebrun P, Houari S, Ziemons E, Rozet E, Hubert P. Improvement of a stability-indicating method by Quality-by-Design versus Quality-by-Testing: A case of a learning process. Journal of pharmaceutical and biomedical analysis. 2014;88:401-9.
12. Badawy SI, Narang AS, LaMarche KR, Subramanian GA, Varia SA, Lin J, et al. Integrated Application of Quality-by-Design Principles to Drug Product Development: A Case Study of Brivanib Alaninate Film–Coated Tablets. Journal of pharmaceutical sciences. 2016;105(1):168-81.
13. Shah V, Sharma M, Pandya R, Parikh RK, Bharatiya B, Shukla A, et al. Quality by Design approach for an in situ gelling microemulsion of Lorazepam via intranasal route. Materials Science and Engineering: C. 2017;75:1231-41.
14. Dubey A, Boukouvala F, Keyvan G, Hsia R, Saranteas K, Brone D, et al. Improvement of tablet coating uniformity using a quality by design approach. AAPS PharmSciTech. 2012;13(1):231-46.
15. Mazumder S, Pavurala N, Manda P, Xu X, Cruz CN, Krishnaiah YS. Quality by Design approach for studying the impact of formulation and process variables on product quality of oral disintegrating films. International Journal of Pharmaceutics. 2017.
16. Verma S, Lan Y, Gokhale R, Burgess DJ. Quality by design approach to understand the process of nanosuspension preparation. International Journal of Pharmaceutics. 2009;377(1):185-98.
17. Huang J, Kaul G, Cai C, Chatlapalli R, Hernandez-Abad P, Ghosh K, et al. Quality by design case study: an integrated multivariate approach to drug product and process development. International journal of pharmaceutics. 2009;382(1):23-32.
18. Fahmy R, Kona R, Dandu R, Xie W, Claycamp G, Hoag SW. Quality by design I: application of failure mode effect analysis (FMEA) and Plackett–Burman design of experiments in the identification of “main factors” in the formulation and process design space for roller-compacted ciprofloxacin hydrochloride immediate-release tablets. AAPS PharmSciTech. 2012;13(4):1243-54.
19. Silva BM, Vicente S, Cunha S, Coelho JF, Silva C, Reis MS, et al. Retrospective Quality by Design (rQbD) applied to the optimization of orodispersible films. International Journal of Pharmaceutics. 2017;528(1-2):655-63.
20. Charoo NA, Shamsher AA, Zidan AS, Rahman Z. Quality by design approach for formulation development: a case study of dispersible tablets. International journal of pharmaceutics. 2012;423(2):167-78.
21. Terzić J, Popović I, Stajić A, Tumpa A, Jančić-Stojanović B. Application of Analytical Quality by Design concept for bilastine and its degradation impurities determination by hydrophilic interaction liquid chromatographic method. Journal of pharmaceutical and biomedical analysis. 2016;125:385-93.

22. Hales D, Vlase L, Porav SA, Bodoki A, Barbu-Tudoran L, Achim M, et al. A quality by design (QbD) study on enoxaparin sodium loaded polymeric microspheres for colon-specific delivery. European Journal of Pharmaceutical Sciences. 2017;100:249-61.
23. Kovács A, Berkó S, Csányi E, Csóka I. Development of nanostructured lipid carriers containing salicyclic acid for dermal use based on the Quality by Design method. European Journal of Pharmaceutical Sciences. 2017;99:246-57.
24. Garg NK, Sharma G, Singh B, Nirbhavane P, Tyagi RK, Shukla R, et al. Quality by Design (QbD)-enabled development of aceclofenac loaded-nano structured lipid carriers (NLCs): An improved dermatokinetic profile for inflammatory disorder (s). International journal of pharmaceutics. 2017;517(1):413-31.
25. Sovány T, Csordás K, Kelemen A, Regdon G, Pintye-Hódi K. Development of pellets for oral lysozyme delivery by using a quality by design approach. Chemical Engineering Research and Design. 2016;106:92-100.
26. Amasya G, Badilli U, Aksu B, Tarimci N. Quality by design case study 1: Design of 5-fluorouracil loaded lipid nanoparticles by the W/O/W double emulsion—Solvent evaporation method. European Journal of Pharmaceutical Sciences. 2016;84:92-102.
27. Wurth C, Demeule B, Mahler H-C, Adler M. Quality by design approaches to formulation robustness—an antibody case study. Journal of pharmaceutical sciences. 2016;105(5):1667-75.
28. Adam S, Suzzi D, Radeke C, Khinast JG. An integrated Quality by Design (QbD) approach towards design space definition of a blending unit operation by Discrete Element Method (DEM) simulation. European Journal of Pharmaceutical Sciences. 2011;42(1):106-15.
29. Jana S, Ali SA, Nayak AK, Sen KK, Basu SK. Development of topical gel containing aceclofenac-crospovidone solid dispersion by "Quality by Design (QbD)" approach. Chemical Engineering Research and Design. 2014;92(11):2095-105.
30. Maltesen MJ, Bjerregaard S, Hovgaard L, Havelund S, Van De Weert M. Quality by design–Spray drying of insulin intended for inhalation. European Journal of Pharmaceutics and Biopharmaceutics. 2008;70(3):828-38.
31. Schmidt AH, Molnár I. Using an innovative Quality-by-Design approach for development of a stability indicating UHPLC method for ebastine in the API and pharmaceutical formulations. Journal of pharmaceutical and biomedical analysis. 2013;78:65-74.
