# Supplementary material for: Application of quality by design for 3D printed bone prostheses and scaffolds
Source: PLoS One. 2018 Apr 12;13(4):e0195291. doi: 10.1371/journal.pone.0195291 (PMC5896968; doi:10.1371/journal.pone.0195291)
Supplement: S2 File — (PDF) [file pone.0195291.s002.pdf]

Supplementary material S2: Semi-structured interviews

Application of quality by design for 3D printed bone prostheses and scaffolds

Daniel. Martinez-Marquez[1], Ali. Mirnajafizadeh[2], Christopher P. Carty [3,4,5], Rodney A. Stewart[1*]

[1] School of Engineering, Griffith University, Gold Coast, Queensland, Australia

[2] Molecular Cell Biomechanics Laboratory, University of Berkeley, Berkeley, California, USA

[3] School of Allied Health Sciences and Innovations in Health Technology, Menzies Health Institute Queensland, Griffith University, Gold Coast, Queensland, Australia

[4] Centre for Musculoskeletal Research, Menzies Health Institute Queensland, Griffith University, Gold Coast, Queensland, Australia

[5] Queensland Children's Gait Laboratory, Queensland Paediatric Rehabilitation Service, Children's Health Queensland Hospital and Health Service, Brisbane, Queensland, Australia

* Rodney A. Stewart

Email: r.stewart@griffith.edu.au (RAS)

1. Overview

Since the introduction of QbD into the pharmaceutical field in 2004, it has been widely used in different pharmaceutical fields to enhance formulation and process design (Fahmy et al., 2012), improve of drug manufacture (Badawy et al., 2016; Hubert et al., 2014; Zhang, Yan, Gong, Lawrence, & Qu, 2013), and to develop nano based pharmaceutical products (Cun et al., 2011; Raina, Kaur, & Jindal, 2017; Verma, Lan, Gokhale, & Burgess, 2009). Moreover, through a systematic search and a statistical analysis (S1 File) it was found that the implementation of QbD can provide remarkable results enhancing process and product understanding, and leading to a drastic (up to 90%) reduction of experimental runs. Additionally, it was found that QbD was mainly used to achieve four distinct objectives: process understanding (PU), prediction and optimization (PO), reduction of experimental runs (RER), and development of robust manufacturing methods (DRM).

Based on the positive results that QbD has being providing for the pharmaceutical field, we believe that the adaptation of QbD for the development of 3D printed bone prostheses and scaffolds can provide similar outcomes. As a result, this study formulated and implemented the first five steps (1-5.3) of the QbD system for the development of 3D printed bone prostheses and scaffolds. However, in order to know if this adaptation of QbD will be useful for the development of 3D printed bone prostheses and scaffolds, we validated our results with several experts in the field of study. For this purpose we performed semi-structured interviews to achieve the following objectives:

a) Introduce the concept of the QbD system to a selected group of experts notably researchers and surgeons.

b) To compare the QbD system with business as usual practices and obtain comments and examples on how QbD system can improve current practices.
c) To study the perceptions of researchers and practitioners in relation to the results of the adaptation of QbD for custom 3D printed bone implants with the four previously identified (statistical analysis) potential benefits that QbD can provide to their field of expertise.

2. Materials and methods

In order to validate the results of the adaptation of QbD for custom 3D printed bone implants, a qualitative research approach was employed. For this purpose, semi-structured interviews were specifically designed to capture the experiences and opinions of researchers, industry experts, and practitioners with previous experience in at least one of the main processes necessary for the design and fabrication of custom 3D printed bone implants.

3. Data collection

The design and fabrication of custom 3D printed bone implants requires a multi-disciplinary team of experts of different fields specifically medicine, engineering, and bone biology. Therefore, qualitative semi-structured interviews were selected as the data collection instrument to be performed with each participating. For the purpose of this study, the exploratory approach was adopted following the Consolidated criteria for reporting qualitative research (COREQ) (Tong, Sainsbury, & Craig, 2007), as the aim was to validate the empirically implemented QbD system. By following this approach, 26 questions in total divided in four specific groups were designed. Moreover, an interview guide and a PowerPoint presentation were designed to guide the direction of the conversation, present the preliminary results obtained from the adaptation of QbD and the statistical analysis, and to gather new data

that could not be taken into account during the adaptation of QbD system, for more details refer to Appendix A.

4. Study selection

The criteria to select the participants for this study was based on their experience and expertise in the field of study. Therefore, pertinent experts in the field of tissue engineering, medical product development, and orthopaedic surgeons with previous experience with 3D printed bone implants, were selected.

The sample size is limited by the nature of the research field, which is characterized by small samples, but detailed and extensive work (Anderson, 2010). Therefore, the snowball sampling method was selected to cover as many people the researcher can gain access to. Snowball sampling is when the research benefits from participants network, and others participants are suggested or introduced to the researcher (Harrell & Bradley, 2009). Eight invitations to participate in this study were send via email to different experts of different universities in Australia and USA.

5. Data extraction and analysis

To facilitate the data extraction an interview guide was used to maintain the direction of the conversation and the relevant lines of enquiry, whilst probing into the issues of interest. To obtain a complete and accurate description of the interviewee's responses and comments, all interviews were recorded and transcribed for further analysis. Moreover, at the beginning of each interview a consent form was delivered to each participant, explaining that the information that they will provide will be considered confidential and the summary of results of this study may be used for presentations and publications. Consequently, each participant signed the consent form and approved the interview be audio recorded. The types of questions

that this research addressed were *descriptive* and *structural*. *Descriptive* questions are asked to get descriptions of things and processes in order to get insights, or to check validity or accuracy about something (Harrell & Bradley, 2009). *Structural* questions help the researcher to categorize groups of things and processes and to understand its relationships (Harrell & Bradley, 2009).

Qualitative data analysis required to examine, categorize, tabulate, test and combine evidence to address the initial propositions of a study (Yin, 2014). The data analysis for the semi-structured interviews followed two key steps recommended by Eisenhardt (1989): within-case and cross-case analyses. In this study the within-case analysis was concerned with the evaluation of the collected data, as well as the reporting of the findings of each individual case study. A systematic reading through each transcript was performed, to then assign codes to tag segments with similar content to sort them into separate categories for a final distillation into major themes (Appendix B). The codes were pre-designed using the deductive coding technique based on the five groups of questions designed for the interviews (Stemler, 2001). It is worth mentioning that due to the open ended nature of the interview questions, the answers for a particular question were be mixed up with another question. As result, part of the data gathered was of an "unstructured" nature consisting of long paragraphs which were organized in a structured and evidentiary-based manner to be able to draw conclusions as the study progresses. The information obtained from each interview provided an insight into how different factors of the adapted QbD system were perceived by experts from different fields of expertise. This was presented in the form of evidentiary tables containing the classified evidence from each interview using Miles, Huberman, and Saldaña (2014) tabular approach, for more details refer to Appendix C.

Following this, the cross-case analysis was performed to find patterns, agreements, and disagreements in opinions between the interviewees (McCutcheon & Meredith, 1993). To

facilitate the cross-case analysis, the information in the form of evidence extracts, was categorized in tabular manner based on the coding used, for more details refer to Appendix D. Once all the evidence had been organised, the results of this analysis were used to enhance and validate the preliminary results obtained from the adaptation of the QbD approach, and to report participants' opinions and concerns. It has to be noted that the pattern-matching procedure involves no precise comparisons; there may not be quantitative or statistical criteria involved on which to judge the pattern, thus allowing for some interpretive discretion on the part of the researcher (Yin, 2003).

6. Results

A total of six semi-structured interviews were performed in October 2017. To maintain the participants' confidentiality and anonymity a code was assigned to each of them as A, B, C, D, E, and F. Overall, the participants' expertise comprised a good mix of researchers, industry experts, and medical practitioners of different fields related to medical device development, 3D bone printed implants, motion capture, tissue engineering, orthopaedic surgery, bone biomechanics, computational neuromuscular modeling, and nano engineered implants. Participants' details such as years of experience, and area of expertise and research are summarized in Table 1. The duration of each interview ranged from 30 to 90 minutes. Four sets of face to face interviews were conducted in Australia, and two via video conference in Australia and USA. All interviews were carried out by the first author of this paper on a one-to-one basis.

Table 1. Interview participants' background information

Participants details	Participants' assigned code					
	A	B	C	D	E	F
Research field	Tissue Engineering of cardiovascular implants	Orthopedic Surgery	Computational Neuromuscular Modeling	Biomedical Engineering with emphasis on bone biomechanics, and spinal disorders and biomechanics	Biomechanics	Nano engineered implants
Current position and responsibilities	Principal Engineer in research and product development in a medical device company	Visiting Medical Officer, Paediatric Orthopaedic Surgeon	Professor and Chair of orthopaedic research and education, and lead of Innovations in health technology	Professor and chair of biomedical engineering of spinal disorders	Clinical motion analysis consultant	Research fellow Postdoctoral researcher
Field of education	PhD in Cardiovascular Engineering, Master in Biomedical Engineering, and bachelor in Mechanical Engineer	Bachelor of Science, and Bachelor of Medicine	Postdoc in Neurophysiology, PhD in Biomechanics, and Bachelor in Mechanical Engineering	Postdoc in Bioengineering, PhD in Mechanics of Materials, Master in Theoretical Mechanics.	PhD in Biomechanics and Orthopaedics, and Bachelor degree in human movement science	PhD in Nano technology, and Bachelor in Nano-biotechnology
Interview type and participant location	Online. Irvine, California, USA	Office. Brisbane, Queensland, Australia	Office. Gold Coast, Queensland, Australia	Office. Brisbane, Queensland, Australia	Online, Brisbane, Queensland, Australia	Office, Brisbane, Queensland, Australia
Years of experience	20	7	38	16	13	8
Number of projects in which they had participated	14 major industry projects	4 major medical projects	30 research and industry projects	40 research and industry projects	4 major research and medical projects	20 research projects

Previously in the statistical analysis (S1 File) it was identified that the main objectives to use the QbD system in the pharmaceutical sector were: process understanding (PU), prediction and optimization (PO), reduction of experimental runs (RER), and development of robust manufacturing methods (DRM). Comparing this results with the interviews responses, it was found that there is a positive common consensus between participants' responses in relation to the main four reasons that the adaptation of the QbD system can be used in the

development of custom 3D printed bone implants. Moreover, during the interviews the participants commented about other potential benefits that QbD can provide to this field, as well as concerns and suggestions.

Five out of six participants agree that the adaptation of QbD facilitates PU by providing a framework in a flowchart fashion that allows to understanding the whole development process in an easier way. *"This is an enhancement in streamlining the whole product development process… to aid new companies… and giving to them a framework to understand the development process"* (Participant B). Moreover, QbD "*allow to simulate possible variations on design and see what is important and what is not important*" (Participant C). "*have a number of factors that can influence the quality of the product and write them down in a more flowchart fashion then it is easier to find out what is the step that is missing or if it needs more control"* (Participant F).

In regards to PO and RER the interview participants stated that "*this is a huge capability that QbD has"* (Participant A, tissue engineering expert), "*this could be a good way to reduce the number of experiments"* (Participant D). *Moreover,* "*QbD will tell us the most crucial things that we should do in experiments… because allow us to look the most sensitive parameters for the required outcomes. For example, wind design used to be empirical, in the transition from subsonic to supersonic. Design engineers use to think that they can find a wind design that allow for a smooth transition with laminar flow, but actually it was shown mathematically that can never exist, and that stop them for looking the solution for this problem. So using the same analogy we can also use predictions that will tell us what we can do and what we cannot do"* (participant C).

In relation to DRM, all participants agree that the adaptation of QbD can help to achieve robust manufacturing process, improving biomedical engineering products by minimising the

risks and reducing product variation. *"I can see that this method can be the replacement to the Six Sigma method, which is really effective for conventional manufacturing, but in the case of 3D printing this is totally different, that's why I think that QbD can work here"* (Participant A). "*QbD feedback loops can ensure that the fabrication produces what we are expecting*" (Participant C). "*There is not a streamline process that we can really rely yet. So the fact that now the QbD process very systematically brakes down into sections which later people can take on and then develop independently, this will be a big step towards making the process more efficient and improve time frames*" (Participant B).

Through the interviews other potential benefits that QbD can provide for the development of 3D printed bone implants products were explored. These benefits are: direction to a clear goal (D-GOAL); improvement of current practices (IMPRV-PRAC); enhancement of current bone implant and scaffold design (ENHAN); product development acceleration (ACCEL); innovation encouragement (INNOV); and other kinds of potential benefits (O-BENF).

According to the opinions of different experts one of the potential benefits that QbD can offer for the development of such products is the direction of the product development process clearer goals by providing a depth understanding of the numerous factors involved in it, allowing a better definition of product boundaries, targets, and potential modifications. For example: *QbD does this (facilitate the direction of a project) really nicely, because is quite overwhelming to start a research project in this area. QbD simplifies the whole process and your results are a beautiful demonstration of the process* (Participant B). *By checking all the boxes in QbD, I would suggest you can better define the direction of your project and also the research question, in particular in determining the boundaries of the product may encompass, you can determine where your product is to be targeted then also how much modifications you can see within that process* (Participant E).

According to Participants B and C the adaptation of the QbD can enhance current design practices and accelerate product development by breaking down the whole development process into easily understood sections that can independently be developed under the same goal. This results in a more efficient, streamlined process as well as the reduction of product development time frames, and the help to reduce a high risk industry into something that is very low risk. *"There are all sorts of ideas coming up from doctors and surgeons, but how to do this and how to do it better, are just intuitive guesses. This (QbD) is actually a framework…that allows you to test those intuitions correctly and rapidly. It actually allows you to play around with… and rapidly test different ideas"* (Participant C). "*Using more lean techniques to establish models that have not been used in this area can save us a lot of time"* (Participant F). *QbD can improve efficiency within the service and accelerate the use of the technology …and give confidence to produce products as good or even better that current designs* (Participant E).

QbD by design also "*can act as an insurance for innovation to be truly examined and analysed"* (Participant A). Moreover, *"having the backing of QbD it will definitely assist innovation towards commercialization"* (Participant E). All this can be possible due to the fact that QbD is a tool that facilitates a complete understanding of the product and its fabrication processes, allowing to short test products and ideas to find optimal ranges of operation that then can be extrapolated to invent new products (Participant D).

Other potential benefits were perceived by the participants of this study, for example: Participant B stated that QbD can facilitate communication between experts from different fields, cutting out work throughout the project and saving a lot of trouble and cost. Moreover, participants B and C mentioned that the use of QbD and computational modelling will be critical to develop personalised biological implants, because they *"does not allow destructive testing, and will required millions of hours of physical testing just to one product"* (Participant

C). Furthermore, participants D, E, and F identified that there is a need for regulations in this area, and QbD can pave the way the way for future enhancement in this research field and act as a guidance for regulatory bodies across countries such as FDA (USA), and TGA (Australia) to develop new quality standards for these emerging customized products, thus *"they can function better, brake less, and give better outcomes"* (Participant C).

In regards to the participants' concerns, three of them said that the challenge is to convince industry and researchers to use the QbD system. Moreover, they point out that the biggest challenge could be the integration of the system, and the start-up time and cost to implement it. Additionally, some participants suggested few improvements to the adaptation of QbD, which were accordingly addressed in order to enhance our preliminary results.

7. Conclusions

Overall, the interview results show a positive common consensus between participants' responses agreeing that adapted QbD system of this study can be used to achieve the same benefits that the QbD has been providing to the pharmaceutical sector. These four benefits where previously identified in the statistical analysis (S1 File), they are: process understanding (PU), prediction and optimization (PO), reduction of experimental runs (RER), and development of robust manufacturing methods (DRM). Moreover, during the interviews the participants commented about different potential benefits that QbD can provide to this field, including some concerns and suggestions.

According to the opinions of participating experts, one of the potential benefits that QbD can offer for the development of such products is the direction of the product development process clearer goals by providing a depth understanding of the numerous factors involved in it, allowing a better definition of product boundaries, targets, and potential modifications. Moreover, according to participants' comments the results of the adaptation of the QbD can

enhance current design practices and accelerate product development by breaking down the whole development process into ease understandable sections that can independently be developed under the same goal. The results of it is a more efficient streamline process towards the reduction of product development time frames, and the help to reduce a high risk industry in a situation that is really low risk. Furthermore, QbD by design also "*can act as an insurance for innovation to be truly examined and analysed"* (Participant A). Moreover, *"having the backing of QbD it will definitely assist innovation towards commercialization"* (Participant E). All this can be possible due to the fact that QbD is a tool that facilitates a complete understanding of the product and its fabrication processes, allowing short testing of products and ideas to find optimal ranges of operation that then can be extrapolated to invent new products (Participant D).

Other potential benefits that were perceived are that QbD can facilitate communication between experts, because it breaks down the whole development process into coherent sections cutting out work throughout the project and saving a lot of trouble and cost. Moreover, it was mentioned that the use of QbD and computational modelling will be critical for the development of personalised biological implants. Furthermore, three participants identified that there is a need for regulations in this area, and QbD can pave the way for future enhancement in this research field, acting as a guide for regulatory bodies across countries such as FDA (USA), and TGA (Australia) to develop new quality standards for emerging customized medical products, thus *"they can function better, brake less, and give better outcomes"* (Participant C).

References

Anderson, C. (2010). Presenting and evaluating qualitative research. *American journal of pharmaceutical education, 74*(8).

Badawy, S. I., Narang, A. S., LaMarche, K. R., Subramanian, G. A., Varia, S. A., Lin, J., . . . Shah, P. A. (2016). Integrated Application of Quality-by-Design Principles to Drug Product Development:

A Case Study of Brivanib Alaninate Film–Coated Tablets. *Journal of pharmaceutical sciences, 105*(1), 168-181.

Cun, D., Jensen, D. K., Maltesen, M. J., Bunker, M., Whiteside, P., Scurr, D., . . . Nielsen, H. M. (2011). High loading efficiency and sustained release of siRNA encapsulated in PLGA nanoparticles: quality by design optimization and characterization. *European journal of pharmaceutics and biopharmaceutics, 77*(1), 26-35.

Eisenhardt, K. M. (1989). Building Theories from Case Study Research. *The Academy of Management Review, 14*(4), 532-550. doi:10.5465/AMR.1989.4308385

Fahmy, R., Kona, R., Dandu, R., Xie, W., Claycamp, G., & Hoag, S. W. (2012). Quality by design I: application of failure mode effect analysis (FMEA) and Plackett–Burman design of experiments in the identification of “main factors” in the formulation and process design space for roller-compacted ciprofloxacin hydrochloride immediate-release tablets. *AAPS PharmSciTech, 13*(4), 1243-1254.

Harrell, M. C., & Bradley, M. A. (2009). *Data collection methods. Semi-structured interviews and focus groups*. Retrieved from

Hubert, C., Lebrun, P., Houari, S., Ziemons, E., Rozet, E., & Hubert, P. (2014). Improvement of a stability-indicating method by Quality-by-Design versus Quality-by-Testing: A case of a learning process. *Journal of pharmaceutical and biomedical analysis, 88*, 401-409.

McCutcheon, D. M., & Meredith, J. R. (1993). Conducting case study research in operations management. *Journal of Operations Management, 11*(3), 239-256. doi:10.1016/0272-6963(93)90002-7

Miles, M. B., Huberman, A. M., & Saldaña, J. (2014). *Qualitative data analysis: a methods sourcebook* (Third ed.). Thousand Oaks, Califorinia: SAGE Publications, Inc.

Raina, H., Kaur, S., & Jindal, A. B. (2017). Development of efavirenz loaded solid lipid nanoparticles: Risk assessment, quality-by-design (QbD) based optimisation and physicochemical characterisation. *Journal of Drug Delivery Science and Technology, 39*, 180-191.

Stemler, S. (2001). An overview of content analysis. *Practical assessment, research & evaluation, 7*(17), 137-146.

Tong, A., Sainsbury, P., & Craig, J. (2007). Consolidated criteria for reporting qualitative research (COREQ): a 32-item checklist for interviews and focus groups. *International Journal for Quality in Health Care, 19*(6), 349-357.

Verma, S., Lan, Y., Gokhale, R., & Burgess, D. J. (2009). Quality by design approach to understand the process of nanosuspension preparation. *International journal of pharmaceutics, 377*(1), 185-198.

Yin, R. K. (2014). *Case study research: design and methods* (Fifth ed.). Los Angeles: SAGE.

Zhang, L., Yan, B., Gong, X., Lawrence, X. Y., & Qu, H. (2013). Application of quality by design to the process development of botanical drug products: a case study. *AAPS PharmSciTech, 14*(1), 277-286.

Appendix A
Interview Guide

Topic
Potential benefits, improvements, and challenges of the Quality by Design (QbD) system in research and industry.

Objectives

1. Introduce the concept of the QbD system to a selected group of experts such as researchers and medical doctors.

2. To compare the QbD system against business as usual practices and obtain comments and examples on how QbD system can improve current practices.

3. To study the perceptions of researchers and medical doctors in relation to the four previously identified (statistical analysis) potential benefits that QbD can provide to their field of expertise.

Interview questions and steps

1. **Background information**

   1.1. Research field
   1.2. Current position and responsibilities
   1.3. Field of education and research
   1.4. Years of experience
   1.5. Number of project in which you had participated

2. **Introduction of the QbD system adapted for custom 3D printed bon prostheses and scaffolds**

General (objective 1): In the following minutes I will introduce and explain the concept of the QbD system, including the results of the adaptation of this system for the development of 3D printed bone prostheses and scaffolds (refer to page 4).

- Quality target product profile
- Critical quality attributes
- Process flow diagram
- Critical process parameters and material attributes
- Risk assessment
- Design Space and Design of experiments
- Quality control strategies
- Product lifecycle management and continuous improvement

3. Compare the QbD system against business as usual practices

General (objectives 2): Please think about the business as usual practices in your field.

3.1. Can you tell me if you used something similar to QbD in previous projects?

3.2. Can you please tell me, what are the differences that you can perceive between businesses as usual practices and the QbD system?

- **Probe on :** QbD can help to better direct the development process to a clear goal than current practices

3.3. To what extent do you think that QbD can facilitate the direction of a project/product development?

- **Probe on:** QbD can accelerate the product/research development process

3.4. Do you believe that QbD system can help to accelerate product/research development process?

3.5. If yes, can you give some examples based on current practices?

- **Probe on:** Comprehensives of QbD risk assessment compared to current practices

3.6. Can you tell me if you used something similar in previous projects?

3.7. Can you tell me which risk assessment is more comprehensive?

3.8. If yes, can you give some examples based on current practices?

3.9. What other kind of potential benefits do you believe that QbD system can provide to 3D printing in the medical field in relation to bone implants and medical devices?

3.10. Do you believe that using QbD system can improve current practices?

4. <u>Perception of potential benefits of the QbD system</u>

General (objective 3): Please think about your previous experience in relation to 3D printing.

- **Probe on:** Process and product understanding

4.1. To what extent do you believe that the QbD system can enhance process and product understanding?

- **Probe on:** Prediction and optimization

4.2. To what extent do you believe that the QbD system can help to predict experimental results?

4.3. To what extent do you believe that the QbD system can facilitate optimization of experiments and processes?

- **Probe on:** Reduction of experimental runs

4.4. To what extent do you believe that the QbD system can help to reduce experimental runs without affect the quality of your experiments?

- **Probe on:** Development of robust processes and manufacturing

4.5. To what extent do you believe that the QbD system can assist for the development of more robust processes and manufacturing practices?

5. <u>Other potential benefits of the QbD system</u>

5.1. Can you tell me some examples on how QbD can help you in future projects?

5.2. Do you have a quality control strategy to ensure that your project/process can provide consistent and reliable outcomes?

5.3. Do believe that the application of QbD can lead to innovation?

5.4. To what extend do you believe that the content of the QbD adaptation can enhance current bone implant and scaffold design?

5.5. The solution needs significant improvements?

5.6. Are you keen to apply the solution or aspects of it in my future work?

Appendix B
Coded Interviews Transcript

Table B-1. Coded interviewees' responses transcript

Question No	Participants' assigned code					
	A	B	C	D	E	F
**3.1.** Can you tell me if you used something similar to QbD in previous projects?	No, I have not used this tool and methods in this specific fashion	No haven't use something like this before	No I haven't, but we are trying to implement it in developing new personalized implants and wearable devices. (P-BENF)	No in such extensive way.	I wound say loosely, in terms of designing collection protocols for motion analysis data, we sort observed in the past where errors could occur where thing were missing or data been erroneous, we developed a check list especially with the equipment	No as such, but we try to minimize the risk in simple things like when you are trying to see the variation of one factor is really important to keep all the other factors constant…
**3.2.** Can you please tell me, what are the differences that you can perceive between businesses as usual practices and the QbD system?	We use some of the tools and methods that you mentioned. But we also use important tools such a design history file, and the traceability matrix to communicate with the FDA (CONC-IMPROV)	The companies with who I work they develop similar products and perhaps this process would be really useful to them in terms of development (D-GOAL)	With QbD we will produce personalized products that function better, fit the function of the person, brake less, and give better outcomes. (P-BENF)	Most of scientific projects are relatively narrow, You are talking about a really broad process which is not generally within a research project.	In terms of stablishing the QbD regime it will be difficult to start because there are a lot of check points to go through and you have to investigate a lot of steps within the process. (CONC-IMPROV) But the advantage that I suggest is that once the system is setup is very easy to ensure that all the processes are correct and followed, and you are able to identify where the errors are, and quickly fix the contribution of that errors. (P-BENF)	I'm very much interested first of all in reducing the number of experiments (RER), so if you want to see the influence of five factors. And otherwise because I work with applications for bone dental implants, so to go in way to minimize any intervention like the FDA, so if you want to proceed to human trials just follow a protocol to minimize any way. (D-GOAL) (P-BENF)
**3.3.** To what extent do you	Yes (D-GOAL), I think sounds really	What you just show me is quite	I think it will improve our success rates. I		By checking all the boxes in QbD, I	I think is more about of reducing

think that QbD can facilitate the direction of a project/product development?	interesting and exciting. This is going to be a really powerful tool, and if is presented well to industries I think it will be a huge interest on this. (P-BENF)	critical to aid new companies in their product development process, and giving to them a framework to understand the development process. (PU)<br><br>I think QbD does this really nicely, because is quite overwhelming to start a research project in this area. QbD simplifies the whole process and your results are a beautiful demonstration of the process. (D-GOAL)	think if we can harbor this process we can do better (D-GOAL)		would suggest you can better define the direction of your project and also the research question (D-GOAL), in particular in determining the boundaries of the product may encompass , you can determine where your product is to be targeted then also how much modifications you can see within that process. (P-BENF) (PU)<br><br>In the case for regulatory approvals and so forth because all that hasn't been stablished as supposed to. (P-BENF)<br>Getting half way in a project and then realizing that you are not going where should be. (D-GOAL)	the risks for the ultimate aim, and following a path to deal with the FDA or authorities like, and then proceeding with authorities from research to the actual life as a product to the implant market is minimized. (ACCEL). For example, each lab have their own practices to minimize the risk, but recently each technique has been very much optimized to achieve really reproducible results, so I think the reproducibility is one thing which apply to all parts of the process…so everything is scalable but how reproducible it is.
**3.4.** Do you believe that QbD system can help to accelerate product/research development process?	Certainty (ACCEL), Also I can see how QbD can be used to reduce the scrap rate in the manufacturing process (P-BENF)	Absolutely, without doubt (ACCEL)	Definitely, especially now with personalization of implants with 3D printing. I think that QbD and computational modelling will help to design better biological implants. (ENHAN)	Is a tricky question, because all the principle that you said all make sense, the big question is going through all the principles will really improve what is currently done? So we just can hope that it will improve. (ACCEL)<br>(CONC-IMPROV)	I think product development yes (ACCEL). But in terms of research especially in my field, there is such a pressure to continuously publish that definitely corners are cut. (CONC-IMPROV) I think once is stablished, so if you have a specialized person within the research team to ensure the quality or at least investigate and to learn the process, I think it will be quite valuable. But my perception would be that a lot of research would not take the effort to learn the whole process and implement all the procedures. (CONC-IMPROV) For product design, commercialization and innovation I think would be valuable, an approach like that should be implemented if the point of the research is to move to	In research development again taking the example of reducing the number of experiments by using more lean techniques to stablish models that have not been used in this area can save us a lot of time (IMPRV-PRAC). (ACCEL) (P-BENF)., and besides that again most people just stop at proving a point but they don't take care about what is the next step, because our ultimate aim is to have it (TNTs) in the implant market. So to ensure bone integration there are many tricky things to take care of, so we can use this management theory. (IMPRV-PRAC).

					commercialization (ACCEL).	
**3.5.** If yes, can you give some examples based on current practices?		A lot of surgeons have wonderful ideas but in reality when they try to develop it is much more difficult. Is not only about understanding the whole process, but all these different processes need different people who don't necessary know what to speak with each other. So introducing the different sections of QbD to these different people will cut-out work throughout the project saving a lot of troubles and cost. (P-BENF)	In my practice in aeronautical industry I used it. You can see how much the aircraft industry succeeded in reducing a high risk industry to something that is really low risk (IMPRV-PRAC).		What I observed in some students' projects developing 3D models of patient's bones, in the image reconstruction you can perceive that there are errors in the final product, at that point it is really difficult to know where those errors are present and at what point in the process they came about, so in those cases the student had to started again and trying to do a better job, but they may get the same error again. I think in designing the process where you have multiple check points along the way to say: it is my data is good at this point? Can I proceed yes or no? That's something of advantage for (P-BENF). I think with my observation within surgery, just knowing all the constrains from the outsets, for instance if we are designing a product that fits perfectly into the bone will allow the surgeon to cut in a specific place, but then what is not considered within that process some of the clinical indications, so maybe there is a muscle that attaches now through your guide and you haven't even thought about. So came to surgery and try to put this guide that doesn't fit because there is a muscle there. So I think making sure that you have all the information beforehand definitely accelerate the product (IMPRV-PRAC). (D-GOAL) (ACCEL), and will lead to the client, the surgeon in that case, and	

					accelerate the use of the technology (P-BENF). Because if it doesn't fit is most likely that they won't try it again.	
**Prove on:** Comprehensives of QbD risk assessment<br><br>**3.6**. Can you tell me if you used something similar in previous projects?	Yes, but slightly different	Yes, in terms of patient care we use six sigma risk assessment to minimise the number of processes in order to minimise the number of potential errors. But in terms of manufacturing a product we don't.	I think this risks assessment is similar to the one used in aeronautics, but needs to be integrated with computational simulations. (CONC-IMPROV)	Probably not so much because my projects were not relate in developing a prototype to be applied in human, so we don't have to be worried about if there is a risk. I guess the only risk assessment that is generally applied in research projects is related to if you develop a new methodology that hasn't been applied ever what if can't be developed in the way you are thinking, so you need to have a plan B if this doesn't work maybe we could do it in this way. So is risk assessment but in terms of feasibility.	Similar but more primitive	Unfortunately there is nothing like that, so there is a research gap in this area. If you look at the papers, every other paper has their own protocol, things will (are) be disclosed and (are not) won't disclosed because they are not sure about. Simple example is aging the electrolyte, there is no uniformity, people said 'I sterilised the TNTs in UV', but how much time, what wave length what is the distance... So every little factor matters, if you are talking about integrating it to the current implant market, so we really need a protocol of fixed set of rules to follow, especially for the young researchers how are entering this area. (D-GOAL) (P-BENF) (IMPRV-PRAC).
**Prove on:** Comprehensives of QbD risk assessment<br><br>**3.7**. Can you tell me which risk assessment is more comprehensive?	Risk assessment and FMEA are slightly different, however I did not see in your presentation the severity of the risks and consequences to the patient (CONC-IMPROV)	Yes this is a similar process compare with what we do. However, you should include also patient's characteristics such as biology, health condition, because patient infection and defect can change etc. (CONC-IMPROV)			QbD is definitely much more comprehensive the risk management that use now, in motion capture is more focused in calibrating the equipment and the lab properly. So we ensure that the right data is collected. I think QbD provides probably more a template to ensure that the quality of the data is as precise as possible. (D-GOAL) (P-BENF)	Every step matters, so for the real optimised results that are reproducible
**3.8**. If yes, can you give some examples based on current practices?					We do some crosschecks but not as systematic as the process you provided. To add to that the checks	

					that we would implemented are probably more reactive than pre-planned, an error will come about then look where that had error occurred and later implement check to ensure that does not happen again, whereas QbD we are trying to do from the outside is to determine what is scopeable possible and have the process setup from that point of view. (D-GOAL) (P-BENF)	
**3.9.** What other kind of potential benefits do you believe that QbD system can provide to 3D printing in the medical field in relation to bone implants and medical devices?	QbD sounds as a requirement when you are talking about customized manufacturing, I can see that this method can be the replacement to the Six sigma method, which is really effective for conventional manufacturing. But in the case of 3D printing this is totally different, that's why I think that QbD can work here. (IMPRV-PRAC).	If we look it for 3D printing biological material, I think at the moment our technology is not quite there. But we are really close in developing customised prosthetics. However, in the future we need to head for biological products, and that's where this process (QbD) will be critical to develop that sort of technology. (P-BENF)	Personalised implants does not allow destructive testing, because that will required millions of hours of physical testing just to one product. You do not do that in a personalised implant. It has to be by QbD there is no other way to do it. (P-BENF)	Generally there is a need for regulations, at the moment there is no regulatory agreement in that space and also I think we need to try to have that consistency across different countries and universities, because then you can compare the differently produced products (IMPRV-PRAC)., because at the end of the day you will need lots of data to do statistics and see if that works or if that doesn't work. So I think having a document that is a guideline of what you should be doing, is like when you are doing mechanical testing and there is the American's standard and you take that as your standard testing, so if somebody have done that then I should be able to reproduce it and roughly have the same results. In the implant space we don't have that and in order to compare we need something like that, is just a matter of what will people accept overall, (D-GOAL) (P-BENF) because as you see the standards are relatively simple generally, but yours is like a Pandora box, very complex. (CONC-IMPROV)	(P-BENF) Trust is probably the main one, if the clinician and the regulatory approval bodies consider that there is a body of evidence or work showing that a comprehensive risk assessment has been undertaken they are more likely to have confidence that they don't going to get significant issues from using 3D printing (IMPRV-PRAC).. From the point of view of a hospital what they don't want a front page in Korean news……	I think it comes to the rules and regulations by the authorities to be able to be successfully be integrated into the current implant market (IMPRV-PRAC), everything has to be reproducible, scalable, and customised for all the specific patient's needs. (P-BENF)
**3.10**. Do you believe that	Yes, (IMPRV-PRAC).	Yes (IMPRV-PRAC).	Yes, I think it can. For example: A knee	Probably (IMPRV-PRAC).	Definitely, I think it can improve	Yes, this can improve current

using QbD system can improve current practices?	the challenge here is to convince industry and get the 3D printers' qualification (CONC-IMPROV)		implant from "A" company can have between 100 to 1000 combinations, between implant components, how do you select the right component for an individual. You don't, surgeons rely upon on most go to set of components and apply it to a person. But having a digital model of that person will give better outcomes. (IMPRV-PRAC).		current practices and improve efficiency within the service (IMPRV-PRAC), I mean knowing all the issues and when and why those errors occurred (P-BENF). It Is just the start-up time and cost to implement it. (CONC-IMPROV)	practices (IMPRV-PRAC). starting from the very fabrication TNTs to the therapeutic preparation of that (P-BENF)
**4.1.** To what extent do you believe that the QbD system can enhance process and product understanding?	I don't know how to answer that question (PU)	Nothing like this ever existed like this before, so look it from this perspective, this is an enhancement in streamlining the whole product development process. (ACCEL) (IMPRV-PRAC). (PU) So even looking the companies that are well stablished and are gone through the FDA approval they being in part of this process, but I can guarantee you they would not have being as anywhere as streamline as this (QbD), and that's why get the approval of the TGA is so difficult.	Firstly, this will allow to simulate possible variations on design and see what is important and what is not important. (ACCEL) Secondly, QbD requires feedback loops so the outcomes of the product go back into the design process in a rigorous way to analyse why something failed (PU). Moreover, this feedback loops can ensure that the fabrication produces what we are expecting. (DRM)	I think it is a good reminder of what could go wrong with 3D printing and be sure that you do your quality control checks (PU) (D-GOAL)	I haven't had a huge amount of experience with 3D printing, but looking at the process, the QbD process enable you to have all the major areas (P-BENF) (PU), and knowing the settings on the printers and software are the key. (D-GOAL)	I think if you have a number of factors that can influence the quality of the product and write them down in a more flowchart fashion then it is easier to find out what is the step that is missing or if it needs more control (PU) (P-BENF) (D-GOAL). Because I know if you ask someone that is working in this area of someone that is just starting to work in this area there are endless possibilities to go wrong there are numerous factors that people ignore (PU). When you are talking about reproducing results at the nanoscale every parameter most be properly optimised every time, otherwise the results can vary. And if you talk about going into the implant market there is only a certain window of error that is allowed. It has to be within an acceptable range of error for all the qualities…
**4.2.** To what extent do you believe that the QbD system can help to predict experimental results?	Very much so, I think this is a huge capability that QbD has. I think is really effective for design of experiments and reduction of number of experiments (PO)	I'm don't know if that can predict experimental results, but certainly will reduce the number of experiments that need to be conducted (RER), and if this can be adopted by the	Yes, I think that if QbD can include multiscale models, we can predict the outcomes of an implant design and treatment. (PO)	This could be a good way to reduce the number of experiments (PO) (RER),	I think many aspects you can predict base on it (PO), but depends on how good your premeasures are in terms of looking it from a loading environment, so ensuring that we actually understand	If you can control the factors that you don't want to change, and you only vary that factors that you want to see implementing the yield and improving the yield so makes life much easier

		TGA, FDA etc., this can inform researchers and developers what kind of studies need to be performed to don't waste time in studies that doesn't going to lead to good results (IMPRV-PRAC). This will lead the process more to a streamline and this is a good thing. (D-GOAL) (ACCEL)			dynamically what forces will be experienced by the 3D printed prosthesis, so I think we can be confidence that we can predict it reliability and longevity if adequately understood the environment to which the implant will be exposed to (PO). But I'm not sure how well we understand the environment for an atypical child or adult. For example I have patients with cerebral palsy that loading can be a lot different, even just the patter of their walking and the activation of their muscles. So my response is that QbD will enable us to predict outcomes assuming we know the input data.	(PO). As a person who expended hours in lab trying to do different things, but if you know what needs to be done exactly following a path, so I think this will help researchers that are looking for a path towards optimization of it (PO)(PU)
**4.3.** To what extent do you believe that the QbD system can facilitate optimization of experiments and processes?	I think is really effective for design of experiments and reduction of number of experiments (RER)	Yes (PO), can make the development process quicker (ACCEL)	Yes, it will tell us the most crucial things that we should do in experiments on. (PO)	Same answer (PO), that you can basically reduce the number of tests (RER),	Yes (PO)	There numerous things to optimise and control, if you know what to do will make life easier (D-GOAL). And again is that table where you can reduce the number of experiments, I have been in research for so many years and I had no idea that you actually reduce the number of experiments if you follow that table (PO). So there is a lack of integration of actual lab work and stablished models like this (P-BENF).
**4.4.** To what extent do you believe that the QbD system can help to reduce experimental runs without affect the quality of your experiments?	I think it depends of the case. I'm sure that if is used in the right problem definitely will reduce the number of experiments (RER),		Definitely, because allow us to look the most sensitive parameters for the required outcomes. For example, wind design used to be empirical, in the transition from subsonic to supersonic. Design engineers use to think that they can find a wind design that allow for a		Yes (RER), but I need to understand more the theory behind that.	I'm very much interested first of all in reducing the number of experiments (Q 3.2)

			smooth transition with laminar flow, but actually it was shown mathematically that can never exist, and that stop them for looking the solution for this problem. So using the same analogy we can also use predictions that will tell us what we can do and what we cannot do. (RER)			
**4.5.** To what extent do you believe that the QbD system can assist for the development of more robust processes and manufacturing practices?	I think there are steps in QbD that certainly you can help to have robust manufacturing process (DRM).	There is not a streamline process that we can really rely yet. So the fact that now the QbD process very systematically brakes down into sections which later people can take on and then develop independently (IMPRV-PRAC), this will be a big step towards making the process more efficient and improve time frames. (ACCEL) (DRM)	In its complete element it will improve biomedical engineering products, yes, I think there is enough evidence. I think this is a rigorous detailed process that have been suggested here. (DRM)	Yes I believe it can be applied (DRM)	I thing 3D printing in medicine is definitely worthwhile, so I think it will benefit the field	Following protocols will minimise the risk, which will be end product of a total investigation starting from modelling to the numerous mechanisms used in others models and areas and apply it to this area of fabrication of 3D printed implants with nanotubes can pave the way for future enhancement in this area. (ACCEL) (P-BENF).
**5.1.** Can you tell me some examples on how QbD can help you in future projects?				I think if I want to develop an implant for certain tissue like shoulder or leg prosthesis for amputees for example. Think about this context you are projecting ahead how you would go to develop it. (D-GOAL) (ACCEL) (P-BENF)	for instance in a project that I'm looking at virtual correction of children who had hip fracture so they had a deformity in the femur and we are looking cutting guides for the surgeons, so QbD will ensure that the final product more close to optimal at the time of printing (IMPRV-PRAC), but also for the down track of surgical blade plates to hold the correction and loading environment they are going to undertake. So I think QbD would definitely give us a lot more confidence to proceed with that kind of projects at least in terms of prototype that can add up (P-BENF). I think of having the backing of QbD it will definitely assist innovation towards commercialization	A very understanding of the numerous factors… I think is a part of research where you is unavoidable to change a few things annoyingly, when you have things really in a format of a flowchart you know what you have to do (PU) (D-GOAL), you are only allowed to little deviations in this area within than range again make life easier as a researcher to minimise the errors bar. So if we really follow this mechanisms we can reduce the error bar as we generally observe. Just reduce the variation in the size or in the release profile that can be achieve and that is a big achievement. (P-BENF)

5.2. Do you have a quality control strategy to ensure that your project/process can provide consistent and reliable outcomes?		Not me personally, but I imagine that those processes do exist.	Personally no, because we are researching it. How we put QbD and BIM, PLM into a process. How we actually do that? That's I think is the problem at the moment, it doesn't exist in the field. (CONC-IMPROV)	Not really, this is not needed in research projects		Yes we try the best to minimise the variations with are not in our control. For things like any paper mention that anodization was carried in a close system but it is well know that the electrolyte they used is usually hydroscopic so moisture will be absorbed over time, things like this are not really well known so if we have a protocol written down were it describe step by step what could go wrong and what are the chances of other factors which are robust otherwise to go wrong, that will really help the researchers. (P-BENF)
**5.3.** Do believe that the application of QbD can lead to innovation?	I think QbD can act as an insurance for innovation to be truly examined and analysed. The systematic approach that QbD has, can encourage innovation (INNOV)	It alternately lead to better outcomes for those projects that people embark on, yes. Certainty the implementation yes (INNOV).	Certainty, I think that the whole process is innovation (IMPRV-PRAC) (INNOV). It actually allows you to play around with simulations and 3D printing, and really rapidly test different ideas (ACCEL), and that is absolutely critical for innovation in this space (INNOV). There are sorts of ideas coming up from doctors and surgeons, but how to do this and how to do it better, are just intuitive guesses. This (QbD) is actually a framework with the simulations and 3D printing that allows you to test those intuitions correctly and rapidly. (ACCEL)	I guess, in terms that you develop something that is short of tested in certain range and then you just extrapolate a little bit, just by definition the extrapolation still like a new innovation (INNOV), and if you had thought about that, then it will be relatively easy to extend it without having to go again through the whole application process. (D-GOAL) (ACCEL)<br>I think that is the interesting part, it is a good way of thinking, because you may better try to combine the approved products and invent a new one (INNOV). (P-BENF)	Possibly, I don't know if innovation proceeds QbD or QbD can .... Definitely will assist with translation out of innovation (INNOV)	Yes definitely (INNOV), if you avoid the trial and error, the yield and the productivity will increase overall (ACCEL).
**5.4.** To what extend do you believe that the content of the QbD adaptation can enhance current bone implant and scaffold design?	Yes (ENHAN), because this system is much easier to implement (P-BENF)	That depends on the government. If you are able to take it to the people that can make those decisions and convince them that the process is the one that they need to adopt, which I believe you should, then if there are able to implement it and marketed and make it available	I think generally yes. (IMPRV-PRAC). (ENHAN)	I think you would put a timeframe on how long some of those things will take. Because I guess if you present it to a surgeon for somebody that has a big tumour you have two options, you cut his leg off which is you normally do, or you basically 3D printing a prototype in next five days and save this limb. So for me the question is	Definitely will enable us to match current design and give confidence to produce products as good or even better that current designs. (ENHAN)	Yes (ENHAN), because overall this field holds great promise, but there are numerous challenges that needs to be concord before enter to the implant market, and this path can help to overcome those challenges. (ACCEL) (P-BENF)

		to developers, then yes absolutely. (ENHAN)		with your concept I took that to some extend or it is necessary to go the way that surgeons and engineers go at the moment. So might be worth to think in an immediately criteria that give you at least some guidance of what these implants should be capable of. (IMPRV-PRAC) (ENHAN).<br>So depends on the application, for a long term product you can test all things and maybe is not the question of those short term projects. (CONC-IMPROV)		
**5.5.** The solution needs significant improvements?	Yes, it need to include design validation and verification, traceability matrix, and design history file<br>(CONC-IMPROV)	It needs to be simplified in some way, and I need to sit down and go through the individual points, because I can look it and it looks comprehensive but maybe a lot in there, because my lack of knowledge, I'm not able to pick up upon what maybe you had missed. Certainty from the areas I'm involved in looks like you pick everything. But again for somebody that is just starting and does not have an overall picture of what QbD is, it may be look it and don't understand it. It will be nice to have a single page summary of the idea and concept before you break it down.<br>(CONC-IMPROV)	There are some details there that need to be amended to get this other concept together in put in proper simulation in creating 4D models, I think needs to be a central part of what you are proposing. Also rehabilitation is an important component of the success of the implant in the person.<br>(CONC-IMPROV)	You need to have some examples where you applied the concept and show the feasibility of it.<br>(CONC-IMPROV)	Maybe, is the useability of the results, how would someone actually going to implement the whole process (CONC-IMPROV). I would say that this is a comprehensive outline which involves multiple professions, so I think along that process you are going have lots of different teams of people working in stablishing the risk management strategy.	I'm new in this area of optimizing using modelling and other things, but I think the biggest challenge is how you integrate into a system where there are numerous possibilities to go wrong, how you can define how is this the correct way and this is a kind of dice way to do thinks. How can you say that this factor is the real contributing factor and this factors are allowed to a little bit change. So again I think the integration will be challenging.<br>(CONC-IMPROV)
**5.6.** Are you keen to apply the solution or aspects of it in my future work?	No, unfortunately the field and nature of the products that we develop does not allow that	Absolutely	Yes, big time that's what we want to do.	If I have a research project where I want to do product development, then yes.	Yes	Yes, I'm very keen first to all to start to have that system to reduce the number of experiments to a very proper operating flowchart where I know what can go wrong, so I can avoid that.

Appendix C
Within-case evidence extracts

Table C-2. Within-case evidence extract: participant A

	Factors	Descriptive summary
**QbD Potential Benefits**	D-GOAL	• Yes (Q 3.3)
	ACCEL	• Certainty (Q 3.4)
	O-BENF	• I think sounds really interesting and exciting. This is going to be a really powerful tool, and if is presented well to industries I think it will be a huge interest on this (Q 3.3)<br>• Also I can see how QbD can be used to reduce the scrap rate in the manufacturing process (Q 3.4)<br>• QbD sounds as a requirement when you are talking about customized manufacturing (Q 3.9)<br>• This system is much easier to implement (Q 5.4)
	IMPRV-PRAC	• I can see that this method can be the replacement to the Six Sigma method, which is really effective for conventional manufacturing. But in the case of 3D printing this is totally different, that's why I think that QbD can work here (Q 3.9)
	ENHAN	• Yes, because this system is much easier to implement (Q 5.4)
	INNOV	• I think QbD can act as an insurance for innovation to be truly examined and analysed. The systematic approach that QbD has, can encourage innovation (Q 5.3)
**Concerns and improvement of QbD**	CON-IMPROV	• I did not see in your presentation the severity of the risks and consequences to the patient (Q 3.7)<br>• The challenge here is to convince industry and get the 3D printers' qualification (Q 3.10)<br>• Yes, it need to include design validation and verification, traceability matrix, and design history file (Q 5.5)
**Most used reasons for the use of QbD**	PU	• I don't know how to answer that question (Q 4.1)
	PO	• Very much so, I think this is a huge capability that QbD has. (Q 4.2)
	RER	• I think is really effective for design of experiments and reduction of number of experiments (Q 4.3)<br>• I'm sure that if is used in the right problem definitely will reduce the number of experiments (Q 4.4)
	DRM	• I think there are steps in QbD that certainly you can help to have robust manufacturing process (Q 4.5)

Table C-3. Within-case evidence extract: participant B

	Factors	Descriptive summary
**QbD Potential Benefits**	D-GOAL	• The companies with who I work they develop similar products and perhaps this process would be really useful to them in terms of development (Q 3.2)<br>• I think QbD does this (facilitate the direction of a project) really nicely, because is quite overwhelming to start a research project in this area. QbD simplifies the whole process and your results are a beautiful demonstration of the process (Q 3.3)

	ACCEL	• Absolutely, without doubt (Q 3.4)<br>• Nothing like this ever existed like this before, so look it from this perspective, this is an enhancement in streamlining the whole product development process (Q 4.1)<br>• If this can be adopted by the TGA, FDA etc., this can inform researchers and developers what kind of studies need to be performed to don't waste time in studies that doesn't going to lead to good results (Q 4.2)<br>• Yes (facilitate optimization of experiments), can make the development process quicker (Q 4.3)
	O-BENF	• What you just show me is quite critical to aid new companies in their product development process (Q 3.3)<br>• Is not only about understanding the whole process, but all these different processes need different people who don't necessary know what to speak with each other. So introducing the different sections of QbD to these different people will cut-out work throughout the project saving a lot of troubles and cost (Q 3.5)<br>• If we look it for 3D printing biological material, I think at the moment our technology is not quite there… However, in the future we need to head for biological products, and that's where this process (QbD) will be critical to develop that sort of technology (Q 3.9)
	IMPRV-PRAC	• Yes (Q 3.10)<br>• Nothing like this ever existed like this before, so look it from this perspective, this is an enhancement in streamlining the whole product development process (Q 4.1)<br>• There is not a streamline process that we can really rely yet. So the fact that now the QbD process very systematically brakes down into sections which later people can take on and then develop independently, this will be a big step towards making the process more efficient and improve time frames (Q 4.5)
	ENHAN	• That depends on the government. If you are able to take it to the people that can make those decisions and convince them that the process is the one that they need to adopt, which I believe you should, then if there are able to implement it and marketed and make it available to developers, then yes absolutely (Q 5.4)
	INNOV	• It alternately lead to better outcomes for those projects that people embark on, yes. Certainty the implementation yes (Q 5.3)
**Concerns and improvement of QbD**	CON-IMPROV	• you should include also patient's characteristics such as biology, health condition, because patient infection and defect can change etc (Q 3.7)<br>• It needs to be simplified in some way… for somebody that is just starting and does not have an overall picture of what QbD is, it may be look it and don't understand it. It will be nice to have a single page summary of the idea and concept before you break it down (Q 5.5)
**Most used reasons for the use of QbD**	PU	• What you just show me is quite critical to aid new companies in their product development process, and giving to them a framework to understand the development process (Q 3.3)<br>• Nothing like this ever existed like this before, so look it from this perspective, this is an enhancement in streamlining the whole product development process (Q 4.1)
	PO	• Yes (facilitate optimization of experiments), can make the development process quicker (Q 4.3)
	RER	• certainly will reduce the number of experiments that need to be conducted (Q 4.2)
	DRM	• There is not a streamline process that we can really rely yet. So the fact that now the QbD process very systematically brakes down into sections which later people can take on and then develop independently, this will be a big step towards making the process more efficient and improve time frames (Q 4.5)

Table C-4. Within-case evidence extract: participant C

	Factors	Descriptive summary
**QbD Potential Benefits**	D-GOAL	• I think it will improve our success rates. I think if we can harbor this process we can do better (Q 3.3)
	ACCEL	• Firstly, this will allow to simulate possible variations on design and see what is important and what is not important (Q 4.1)<br>• There are sorts of ideas coming up from doctors and surgeons, but how to do this and how to do it better, are just intuitive guesses. This (QbD) is actually a framework with the simulations and 3D printing that allows you to test those intuitions correctly and rapidly (Q 5.3)

		• It actually allows you to play around with simulations and 3D printing, and really rapidly test different ideas (Q 5.3)
	O-BENF	• With QbD we will produce personalized products that function better, fit the function of the person, brake less, and give better outcomes (Q 3.2)<br>• Personalised implants does not allow destructive testing, because that will required millions of hours of physical testing just to one product. You do not do that in a personalised implant. It has to be by QbD there is no other way to do it (Q 3.9)
	IMPRV-PRAC	• In my practice in aeronautical industry I used it. You can see how much the aircraft industry succeeded in reducing a high risk industry to something that is really low risk (Q 3.5)<br>• I think this risks assessment is similar to the one used in aeronautics (Q 3.6)<br>• Yes, I think it can. For example: A knee implant from "A" company can have between 100 to 1000 combinations, between implant components, how do you select the right component for an individual. You don't, surgeons rely upon on most go to set of components and apply it to a person. But having a digital model of that person will give better outcomes (Q 3.10)
	ENHAN	• Definitely, especially now with personalization of implants with 3D printing. I think that QbD and computational modelling will help to design better biological implants (Q 3.4)<br>• Certainty, I think that the whole process is innovation (Q 5.3)<br>• I think generally yes (Q 5.4)
	INNOV	• Certainty, I think that the whole process is innovation. It actually allows you to play around with simulations and 3D printing, and really rapidly test different ideas, and that is absolutely critical for innovation in this space (Q 5.3)
**Concerns and improvement of QbD**	CON-IMPROV	• I think this risks assessment is similar to the one used in aeronautics, but needs to be integrated with computational simulations (Q 3.6)<br>• How we put QbD and BIM, PLM into a process. How we actually do that? That's I think is the problem at the moment, it doesn't exist in the field (Q 5.2)<br>• There are some details there that need to be amended to get this other concept together in put in proper simulation in creating 4D models, I think needs to be a central part of what you are proposing. Also rehabilitation is an important component of the success of the implant in the person (Q 5.5)
**Most used reasons for the use of QbD**	PU	• Firstly, this will allow to simulate possible variations on design and see what is important and what is not important (Q 4.1)<br>• QbD requires feedback loops so the outcomes of the product go back into the design process in a rigorous way to analyse why something failed (Q 4.1)
	PO	• Yes, I think that if QbD can include multiscale models, we can predict the outcomes of an implant design and treatment (Q 4.2)<br>• Yes, it will tell us the most crucial things that we should do in experiments on (Q 4.3)
	RER	• Definitely, because allow us to look the most sensitive parameters for the required outcomes. For example, wind design used to be empirical, in the transition from subsonic to supersonic. Design engineers use to think that they can find a wind design that allow for a smooth transition with laminar flow, but actually it was shown mathematically that can never exist, and that stop them for looking the solution for this problem. So using the same analogy we can also use predictions that will tell us what we can do and what we cannot do (Q 4.4)
	DRM	• QbD requires feedback loops so the outcomes of the product go back into the design process in a rigorous way to analyse why something failed. Moreover, this feedback loops can ensure that the fabrication produces what we are expecting (Q 4.1)<br>• In its complete element it (QbD) will improve biomedical engineering products, yes, I think there is enough evidence. I think this is a rigorous detailed process that have been suggested here (Q 4.5)

Table C-5. Within-case evidence extract: participant D

	Factors	Descriptive summary
**QbD Potential Benefits**	D-GOAL	• Most of scientific projects are relatively narrow, You are talking about a really broad process which is not generally within a research project (Q 3.2)<br>• I think if I want to develop an implant for certain tissue… Think about this context you are projecting ahead how you would go to develop it (Q 5.1)
	ACCEL	•

	O-BENF	• Generally there is a need for regulations, at the moment there is no regulatory agreement in that space and also I think we need to try to have that consistency across different countries and universities, because then you can compare the differently produced products. So I think having a document that is a guideline of what you should be doing, is like when you are doing mechanical testing and there is the American's standard and you take that as your standard testing, so if somebody have done that then I should be able to reproduce it and roughly have the same results. In the implant space we don't have that and in order to compare we need something like that, is just a matter of what will people accept overall (Q 3.9)
	IMPRV-PRAC	• Probably (Q 3.10)
	ENHAN	• So depends on the application, for a long term product you can test all things and maybe is not the question of those short term projects (Q 5.4)
	INNOV	• I guess, in terms that you develop something that is short of tested in certain range and then you just extrapolate a little bit, just by definition the extrapolation still like a new innovation, and if you had thought about that, then it will be relatively easy to extend it without having to go again through the whole application process. I think that is the interesting part, it is a good way of thinking, because you may better try to combine the approved products and invent a new one (Q 5.3)
**Concerns and improvement of QbD**	CON-IMPROV	• (ACCEL) Is a tricky question, because all the principle that you said all make sense, the big question is going through all the principles will really improve what is currently done? So we just can hope that it will improve (Q 3.4)<br>• As you see the standards are relatively simple generally, but yours is like a Pandora box, very complex (Q 3.9)<br>• I think you would put a timeframe on how long some of those things will take. Because I guess if you present it to a surgeon for somebody that has a big tumour you have two options, you cut his leg off which is you normally do, or you basically 3D printing a prototype in next five days and save this limb. So for me the question is with your concept I took that to some extend or it is necessary to go the way that surgeons and engineers go at the moment (Q 5.4)
**Most used reasons for the use of QbD**	PU	• I think it is a good reminder of what could go wrong with 3D printing and be sure that you do your quality control checks (Q 4.1)
	PO	• This could be a good way to reduce the number of experiments (Q 4.2, 4.3)
	RER	• This could be a good way to reduce the number of experiments (Q 4.2)
	DRM	• Yes I believe it can be applied (Q 4.5)

Table C-6. Within-case evidence extract: participant E

	Factors	Descriptive summary
**QbD Potential Benefits**	D-GOAL	• By checking all the boxes in QbD, I would suggest you can better define the direction of your project and also the research question, in particular in determining the boundaries of the product may encompass, you can determine where your product is to be targeted then also how much modifications you can see within that process. In the case for regulatory approvals and so forth because all that hasn't been stablished as supposed to. Getting half way in a project and then realizing that you are not going where should be (Q 3.3)<br>• whereas QbD we are trying to do from the outside is to determine what is scopeable possible and have the process setup from that point of view (Q 3.8)
	ACCEL	• I think product development yes. I think once is stablished, so if you have a specialized person within the research team to ensure the quality or at least investigate and to learn the process, I think it will be quite valuable (Q 3.4)<br>• For product design, commercialization and innovation I think would be valuable, an approach like that should be implemented if the point of the research is to move to commercialization (Q 3.4)<br>• So I think making sure that you have all the information beforehand definitely accelerate the product and will lead to the client, the surgeon in that case, and accelerate the use of the technology (Q 3.5)
	O-BENF	• But the advantage that I suggest is that once the system is setup is very easy to ensure that all the processes are correct and followed, and you are able to identify where the errors are, and quickly fix the contribution of that errors (Q 3.2)<br>• I think in designing the process where you have multiple check points along the way to say: it is my data is good at this point? Can I proceed yes or no? That's something of advantage for (Q 3.5)

		• I think QbD provides probably more a template to ensure that the quality of the data is as precise as possible (Q 3.7)<br>• Trust is probably the main one (potential benefit), if the clinician and the regulatory approval bodies consider that there is a body of evidence of work showing that a comprehensive risk assessment has been undertaken they are more likely to have confidence that they don't going to get significant issues from using 3D printing (Q 3.9)<br>• So I think QbD would definitely give us a lot more confidence to proceed with that kind of projects at least in terms of prototype that can add up (Q 5.1)
	IMPRV-PRAC	• Definitely, I think it can improve current practices and improve efficiency within the service. I mean knowing all the issues and when and why those errors occurred (Q 3.10)
	ENHAN	• Definitely will enable us to match current design and give confidence to produce products as good or even better that current designs (Q 5.4)
	INNOV	• I think of having the backing of QbD it will definitely assist innovation towards commercialization (Q 5.1)<br>• Definitely will assist with translation out of innovation (Q 5.3)
**Concerns and improvement of QbD**	CON-IMPROV	• In terms of stablishing the QbD regime it will be difficult to start because there are a lot of check points to go through and you have to investigate a lot of steps within the process (Q 3.2)<br>• I think product development yes **(ACCEL)**. But in terms of research especially in my field, there is such a pressure to continuously publish that definitely corners are cut. my perception would be that a lot of research would not take the effort to learn the whole process and implement all the procedures (Q 3.4)<br>• It Is just the start-up time and cost to implement it (Q 3.10)<br>• Maybe, is the useability of the results, how would someone actually going to implement the whole process would say that this is a comprehensive outline which involves multiple professions, so I think along that process you are going have lots of different teams of people working in stablishing the risk management strategy (Q 5.5)
**Most used reasons for the use of QbD**	PU	• Looking at the process, the QbD process enable you to have all the major areas, and knowing the settings on the printers and software are the key (Q 4.1)
	PO	• I think many aspects you can predict base on it, but depends on how good your premeasures are in terms of looking it from a loading environment, so ensuring that we actually understand dynamically what forces will be experienced by the 3D printed prosthesis, so I think we can be confidence that we can predict it reliability and longevity if adequately understood the environment to which the implant will be exposed to. So my response is that QbD will enable us to predict outcomes assuming we know the input data (Q 4.2)<br>• Yes (Q 4.3)
	RER	• Yes (Q 4.4)
	DRM	• I thing 3D printing in medicine is definitely worthwhile, so I think it will benefit the field (Q 4.5)<br>• QbD will ensure that the final product more close to optimal at the time of printing (Q 5.1)

Table C-7. Within-case evidence extract: participant F

	Factors	Descriptive summary
**QbD Potential Benefits**	D-GOAL	• if you are talking about integrating it to the current implant market, so we really need a protocol of fixed set of rules to follow, especially for the young researchers how are entering this area (Q 3.6)<br>• A very understanding of the numerous factors… I think is a part of research where you is unavoidable to change a few things annoyingly, when you have things really in a format of a flowchart you know what you have to do you are only allowed to little deviations in this area within than range again make life easier as a researcher to minimise the errors bar (Q 5.1)
	ACCEL	• by using more lean techniques to stablish models that have not been used in this area can save us a lot of time (Q 3.4)
	O-BENF	• I think it comes to the rules and regulations by the authorities to be able to be successfully be integrated into the current implant market, everything has to be reproducible, scalable, and customised for all the specific patient's needs (Q 3.9)
	IMPRV-PRAC	• Yes, this can improve current practices (Q 3.10)

	ENHAN	• so if we have a protocol written down were describes step by step what could go wrong and what are the chances of other factors which are robust otherwise to go wrong, that will really help the researchers (Q 5.2)<br>• Yes, because overall this field holds great promise, but there are numerous challenges that needs to be concord before enter to the implant market, and this path can help to overcome those challenges (Q 5.4)
	INNOV	• Yes definitely, if you avoid the trial and error, the yield and the productivity will increase overall (Q 5.3)
**Concerns and improvement of QbD**	CON-IMPROV	• I'm new in this area of optimizing using modelling and other things, but I think the biggest challenge is how you integrate into a system where there are numerous possibilities to go wrong, how you can define how is this the correct way and this is a kind of dice way to do thinks. How can you say that this factor is the real contributing factor and this factors are allowed to a little bit change. So again I think the integration will be challenging (Q 5.5)
**Most used reasons for the use of QbD**	PU	• I think if you have a number of factors that can influence the quality of the product and write them down in a more flowchart fashion then it is easier to find out what is the step that is missing or if it needs more control (Q 4.1)<br>• Because I know if you ask someone that is working in this area of someone that is just starting to work in this area there are endless possibilities to go wrong there are numerous factors that people ignore (Q 4.1)<br>• As a person who expended hours in lab trying to do different things, but if you know what needs to be done exactly following a path, so I think this will help researchers that are looking for a path towards optimization of it (Q4.2)
	PO	• As a person who expended hours in lab trying to do different things, but if you know what needs to be done exactly following a path, so I think this will help researchers that are looking for a path towards optimization of it (Q 4.2)<br>• There numerous things to optimise and control, if you know what to do will make life easier. I have been in research for so many years and I had no idea that you actually reduce the number of experiments if you follow that table. So there is a lack of integration of actual lab work and stablished models like this (Q 4.3)
	RER	• I'm very much interested first of all in reducing the number of experiments (Q 3.2)
	DRM	• Following protocols will minimise the risk, which will be end product of a total investigation starting from modelling to the numerous mechanisms used in others models and areas and apply it to this area of fabrication of 3D printed implants with nanotubes can pave the way for future enhancement in this area (Q 4.5)<br>• So if we really follow this mechanisms we can reduce the error bar as we generally observe. Just reduce the variation in the size or in the release profile that can be achieve and that is a big achievement (Q 5.1)

Appendix D
Cross-case evidence extracts

Table D-1. Cross-case analysis and results table: Four most used reasons for the use of QbD

	**Factors**	**Descriptive summary**					
		A	B	C	D	E	F
**Most used reasons for the use of QbD**	PU	**NA:**<br>• I don't know how to answer that question (Q 4.1)	**Agree:**<br>• What you just show me is quite critical to aid new companies in their product development process, and giving to them a framework to understand the development process (Q 3.3)<br>• Nothing like this ever existed like this before, so look it from this perspective, this is an enhancement in streamlining the whole product development process (Q 4.1)	**Agree:**<br>• Firstly, this will allow to simulate possible variations on design and see what is important and what is not important (Q 4.1)<br>• QbD requires feedback loops so the outcomes of the product go back into the design process in a rigorous way to analyse why something failed (Q 4.1)	**Agree:**<br>• I think it is a good reminder of what could go wrong with 3D printing and be sure that you do your quality control checks (Q 4.1)	**Agree:**<br>• Looking at the process, the QbD process enable you to have all the major areas, and knowing the settings on the printers and software are the key (Q 4.1)	**Agree:**<br>• I think if you have a number of factors that can influence the quality of the product and write them down in a more flowchart fashion then it is easier to find out what is the step that is missing or if it needs more control (Q 4.1)<br>• Because I know if you ask someone that is working in this area of someone that is just starting to work in this area there are endless possibilities to go wrong there are numerous factors that people ignore (Q 4.1)<br>• As a person who expended hours in lab trying to do different things, but if you know what needs to be done exactly following a path, so I think this will help researchers that are looking for a path towards optimization of it (Q4.2)
	PO	**Agree:**<br>• Very much so, I think this is a huge capability that QbD has. (Q 4.2)	**Agree:**<br>• Yes (facilitate optimization of experiments), can make the development process quicker (Q 4.3)	**Agree:**<br>• Yes, it will tell us the most crucial things that we should do in experiments on (Q 4.3)<br>• I think that if QbD can include multiscale models, we can predict the outcomes of an implant design and treatment (Q 4.2)	**Agree:**<br>• This could be a good way to reduce the number of experiments (Q 4.2, 4.3)	**Agree:**<br>• Yes (Q 4.3)<br>• I think many aspects you can predict base on it… So my response is that QbD will enable us to predict outcomes assuming we know the input data (Q 4.2)	**Agree:**<br>• As a person who expended hours in lab trying to do different things, but if you know what needs to be done exactly following a path, so I think this will help researchers that are looking for a path towards optimization of it (Q 4.2)<br>• There numerous things to optimise and control, if you know what to do will make life easier (Q 4.3)
	RER	**Agree:**<br>• I think is really effective for design of experiments and reduction of number of experiments (Q 4.3)<br>• I'm sure that if is used in the right problem definitely will reduce the number of experiments (Q 4.4)	**Agree:**<br>• Certainly will reduce the number of experiments that need to be conducted (Q 4.2)	**Agree:**<br>• Definitely, because allow us to look the most sensitive parameters for the required outcomes. For example, wind design used to be empirical, in the transition from subsonic to supersonic. Design engineers use to think that they can find a wind design that allow for a smooth transition with laminar flow, but actually it was shown mathematically that can never exist, and that stop them for looking the solution for this problem. So using the same analogy we can also use predictions that will tell us what we can do and what we cannot do (Q 4.4)	**Agree:**<br>• This could be a good way to reduce the number of experiments (Q 4.2)	**Agree:**<br>• Yes (Q 4.4)	**Agree:**<br>• I'm very much interested first of all in reducing the number of experiments (Q 3.2)

	DRM	**Agree:**<br>• I can see that this method can be the replacement to the Six Sigma method, which is really effective for conventional manufacturing. But in the case of 3D printing this is totally different, that's why I think that QbD can work here (Q 3.9)<br>• I think there are steps in QbD that certainly you can help to have robust manufacturing process (Q 4.5).	**Agree:**<br>• There is not a streamline process that we can really rely yet. So the fact that now the QbD process very systematically brakes down into sections which later people can take on and then develop independently, this will be a big step towards making the process more efficient and improve time frames (Q 4.5)	**Agree:**<br>• I think it will improve our success rates. I think if we can harbor this process we can do better (Q 3.3)<br>• QbD requires feedback loops so the outcomes of the product go back into the design process in a rigorous way to analyse why something failed. Moreover, this feedback loops can ensure that the fabrication produces what we are expecting (Q 4.1)<br>• In its complete element it (QbD) will improve biomedical engineering products, yes, I think there is enough evidence. I think this is a rigorous detailed process that have been suggested here (Q 4.5)	**Agree:**<br>• Yes I believe it can be applied (Q 4.5)	**Agree:**<br>• I thing 3D printing in medicine is definitely worthwhile, so I think it will benefit the field (Q 4.5)	**Agree:**<br>• Following protocols will minimise the risk, which will be end product of a total investigation starting from modelling to the numerous mechanisms used in others models and areas and apply it to this area of fabrication of 3D printed implants with nanotubes can pave the way for future enhancement in this area (Q 4.5)<br>• So if we really follow this mechanisms we can reduce the error bar as we generally observe. Just reduce the variation in the size or in the release profile that can be achieve and that is a big achievement (Q 5.1)

Table D-2. Cross-case analysis and results table: QbD Potential Benefits

	**Factors**	**Descriptive summary**					
		A	B	C	D	E	F
**QbD Potential Benefits**	D-GOAL	• Yes (Q 3.3)	• The companies with who I work they develop similar products and perhaps this process would be really useful to them in terms of development (Q 3.2)<br>• I think QbD does this (facilitate the direction of a project) really nicely, because is quite overwhelming to start a research project in this area. QbD simplifies the whole process and your results are a beautiful demonstration of the process (Q 3.3)	• I think it will improve our success rates. I think if we can harbor this process we can do better (Q 3.3)	• Most of scientific projects are relatively narrow, You are talking about a really broad process which is not generally within a research project (Q 3.2)<br>• I think if I want to develop an implant for certain tissue… Think about this context you are projecting ahead how you would go to develop it (Q 5.1)	• By checking all the boxes in QbD, I would suggest you can better define the direction of your project and also the research question, in particular in determining the boundaries of the product may encompass, you can determine where your product is to be targeted then also how much modifications you can see within that process. In the case for regulatory approvals and so forth because all that hasn't been stablished as supposed to. Getting half way in a project and then realizing that you are not going where should be (Q 3.3)<br>• whereas QbD we are trying to do from the outside is to determine what is scopeable possible and have the process setup from that point of view (Q 3.8)	• if you are talking about integrating it to the current implant market, so we really need a protocol of fixed set of rules to follow, especially for the young researchers how are entering this area (Q 3.6)<br>• A very understanding of the numerous factors… I think is a part of research where you is unavoidable to change a few things annoyingly, when you have things really in a format of a flowchart you know what you have to do you are only allowed to little deviations in this area within than range again make life easier as a researcher to minimise the errors bar (Q 5.1)
	ACCEL	• Certainty (Q 3.4)	• Absolutely, without doubt (Q 3.4)<br>• Nothing like this ever existed like this before, so look it from this perspective, this is an enhancement in streamlining the whole product development process (Q 4.1)<br>• If this can be adopted by the TGA, FDA etc., this can inform researchers and developers what kind of studies need to be performed to don't waste time in studies that doesn't going to lead to good results (Q 4.2)<br>• Yes (facilitate optimization of experiments), can make the development process quicker (Q 4.3)	• Firstly, this will allow to simulate possible variations on design and see what is important and what is not important (Q 4.1)<br>• There are sorts of ideas coming up from doctors and surgeons, but how to do this and how to do it better, are just intuitive guesses. This (QbD) is actually a framework with the simulations and 3D printing that allows you to test those intuitions correctly and rapidly (Q 5.3)<br>• It actually allows you to play around with simulations and 3D printing, and really rapidly test different ideas (Q 5.3)	• NA	• I think product development yes. I think once is stablished, so if you have a specialized person within the research team to ensure the quality or at least investigate and to learn the process, I think it will be quite valuable (Q 3.4)<br>• For product design, commercialization and innovation I think would be valuable, an approach like that should be implemented if the point of the research is to move to commercialization (Q 3.4)<br>• So I think making sure that you have all the information beforehand definitely accelerate the product and will lead to the client, the surgeon in that case, and accelerate the use of the technology (Q 3.5)	• By using more lean techniques to stablish models that have not been used in this area can save us a lot of time (Q 3.4)

	O-BENF	• I think sounds really interesting and exciting. This is going to be a really powerful tool, and if is presented well to industries I think it will be a huge interest on this (Q 3.3)<br>• Also I can see how QbD can be used to reduce the scrap rate in the manufacturing process (Q 3.4)<br>• QbD sounds as a requirement when you are talking about customized manufacturing (Q 3.9)<br>• This system is much easier to implement (Q 5.4)	• What you just show me is quite critical to aid new companies in their product development process (Q 3.3)<br>• Is not only about understanding the whole process, but all these different processes need different people who don't necessary know what to speak with each other. So introducing the different sections of QbD to these different people will cut-out work throughout the project saving a lot of troubles and cost (Q 3.5)<br>• If we look it for 3D printing biological material, I think at the moment our technology is not quite there… However, in the future we need to head for biological products, and that's where this process (QbD) will be critical to develop that sort of technology (Q 3.9)	• With QbD we will produce personalized products that function better, fit the function of the person, brake less, and give better outcomes (Q 3.2)<br>• Personalised implants does not allow destructive testing, because that will required millions of hours of physical testing just to one product. You do not do that in a personalised implant. It has to be by QbD there is no other way to do it (Q 3.9)	• Generally there is a need for regulations, at the moment there is no regulatory agreement in that space and also I think we need to try to have that consistency across different countries and universities, because then you can compare the differently produced products. So I think having a document that is a guideline of what you should be doing, is like when you are doing mechanical testing and there is the American's standard and you take that as your standard testing, so if somebody have done that then I should be able to reproduce it and roughly have the same results. In the implant space we don't have that and in order to compare we need something like that, is just a matter of what will people accept overall (Q 3.9)	• But the advantage that I suggest is that once the system is setup is very easy to ensure that all the processes are correct and followed, and you are able to identify where the errors are, and quickly fix the contribution of that errors (Q 3.2)<br>• I think in designing the process where you have multiple check points along the way to say: it is my data is good at this point? Can I proceed yes or no? That's something of advantage for (Q 3.5)<br>• I think QbD provides probably more a template to ensure that the quality of the data is as precise as possible (Q 3.7)<br>• Trust is probably the main one (potential benefit), if the clinician and the regulatory approval bodies consider that there is a body of evidence of work showing that a comprehensive risk assessment has been undertaken they are more likely to have confidence that they don't going to get significant issues from using 3D printing (Q 3.9)<br>• So I think QbD would definitely give us a lot more confidence to proceed with that kind of projects at least in terms of prototype that can add up (Q 5.1)	• I think it comes to the rules and regulations by the authorities to be able to be successfully be integrated into the current implant market, everything has to be reproducible, scalable, and customised for all the specific patient's needs (Q 3.9)

Table D-3. Continuation of Within-case analysis and results: QbD Potential Benefits

	Factors	Descriptive summary					
		A	B	C	D	E	F
**QbD Potential Benefits**	IMPRV-PRAC	• I can see that this method can be the replacement to the Six Sigma method, which is really effective for conventional manufacturing. But in the case of 3D printing this is totally different, that's why I think that QbD can work here (Q 3.9)	• Yes (Q 3.10)<br>• Nothing like this ever existed like this before, so look it from this perspective, this is an enhancement in streamlining the whole product development process (Q 4.1)<br>• There is not a streamline process that we can really rely yet. So the fact that now the QbD process very systematically brakes down into sections which later people can take on and then develop independently, this will be a big step towards making the process more efficient and improve time frames (Q 4.5)	• In my practice in aeronautical industry I used it. You can see how much the aircraft industry succeeded in reducing a high risk industry to something that is really low risk (Q 3.5)<br>• I think this risks assessment is similar to the one used in aeronautics (Q 3.6)<br>• Yes, I think it can. For example: A knee implant from "A" company can have between 100 to 1000 combinations, between implant components, how do you select the right component for an individual. You don't, surgeons rely upon on most go to set of components and apply it to a person. But having a digital model of that person will give better outcomes (Q 3.10)	• Probably (Q 3.10)	• Definitely, I think it can improve current practices and improve efficiency within the service. I mean knowing all the issues and when and why those errors occurred (Q 3.10)	• Yes, this can improve current practices (Q 3.10)
	ENHAN	• Yes, because this system is much easier to implement (Q 5.4)	• That depends on the government. If you are able to take it to the people that can make those decisions and convince them that the process is the one that they need to adopt, which I believe you	• Definitely, especially now with personalization of implants with 3D printing. I think that QbD and computational modelling will help to design better biological implants (Q 3.4)	• So depends on the application, for a long term product you can test all things and maybe is not the question of those short term projects (Q 5.4)	• Definitely will enable us to match current design and give confidence to produce products as good or even better that current designs (Q 5.4)	• so if we have a protocol written down were describes step by step what could go wrong and what are the chances of other factors which are robust otherwise to go wrong, that will

			should, then if there are able to implement it and marketed and make it available to developers, then yes absolutely (Q 5.4)	• Certainty, I think that the whole process is innovation (Q 5.3)<br>• I think generally yes (Q 5.4)			really help the researchers (Q 5.2)<br>• Yes, because overall this field holds great promise, but there are numerous challenges that needs to be concord before enter to the implant market, and this path can help to overcome those challenges (Q 5.4)
	INNOV	• I think QbD can act as an insurance for innovation to be truly examined and analysed. The systematic approach that QbD has, can encourage innovation (Q 5.3)	• It alternately lead to better outcomes for those projects that people embark on, yes. Certainty the implementation yes (Q 5.3)	• Certainty, I think that the whole process is innovation. It actually allows you to play around with simulations and 3D printing, and really rapidly test different ideas, and that is absolutely critical for innovation in this space (Q 5.3)	• I guess, in terms that you develop something that is short of tested in certain range and then you just extrapolate a little bit, just by definition the extrapolation still like a new innovation, and if you had thought about that, then it will be relatively easy to extend it without having to go again through the whole application process. I think that is the interesting part, it is a good way of thinking, because you may better try to combine the approved products and invent a new one (Q 5.3)	• I think of having the backing of QbD it will definitely assist innovation towards commercialization (Q 5.1)<br>• Definitely will assist with translation out of innovation (Q 5.3)	• Yes definitely, if you avoid the trial and error, the yield and the productivity will increase overall (Q 5.3)

Table D-4. Within-case analysis and results: Concerns and improvement of QbD

	Factors	Descriptive summary					
		A	B	C	D	E	F
**Concerns and improvement of QbD**	CON-IMPROV	• I did not see in your presentation the severity of the risks and consequences to the patient (Q 3.7)<br>• The challenge here is to convince industry and get the 3D printers' qualification (Q 3.10)<br>• Yes, it need to include design validation and verification, traceability matrix, and design history file (Q 5.5	• you should include also patient's characteristics such as biology, health condition, because patient infection and defect can change etc (Q 3.7)<br>• It needs to be simplified in some way… for somebody that is just starting and does not have an overall picture of what QbD is, it may be look it and don't understand it. It will be nice to have a single page summary of the idea and concept before you break it down (Q 5.5)	• I think this risks assessment is similar to the one used in aeronautics, but needs to be integrated with computational simulations (Q 3.6)<br>• How we put QbD and BIM, PLM into a process. How we actually do that? That's I think is the problem at the moment, it doesn't exist in the field (Q 5.2)<br>• There are some details there that need to be amended to get this other concept together in put in proper simulation in creating 4D models, I think needs to be a central part of what you are proposing. Also rehabilitation is an important component of the success of the implant in the person (Q 5.5)	• ACCEL) Is a tricky question, because all the principle that you said all make sense, the big question is going through all the principles will really improve what is currently done? So we just can hope that it will improve (Q 3.4)<br>• As you see the standards are relatively simple generally, but yours is like a Pandora box, very complex (Q 3.9)<br>• I think you would put a timeframe on how long some of those things will take. Because I guess if you present it to a surgeon for somebody that has a big tumour you have two options, you cut his leg off which is you normally do, or you basically 3D printing a prototype in next five days and save this limb. So for me the question is with your concept I took that to some extend or it is necessary	• In terms of stablishing the QbD regime it will be difficult to start because there are a lot of check points to go through and you have to investigate a lot of steps within the process (Q 3.2)<br>• I think product development yes **(ACCEL)**. But in terms of research especially in my field, there is such a pressure to continuously publish that definitely corners are cut. my perception would be that a lot of research would not take the effort to learn the whole process and implement all the procedures (Q 3.4)<br>• It Is just the start-up time and cost to implement it (Q 3.10)<br>• Maybe, is the useability of the results, how would someone actually going to implement the whole process would say that this is a comprehensive outline which	• I'm new in this area of optimizing using modelling and other things, but I think the biggest challenge is how you integrate into a system where there are numerous possibilities to go wrong, how you can define how is this the correct way and this is a kind of dice way to do thinks. How can you say that this factor is the real contributing factor and this factors are allowed to a little bit change. So again I think the integration will be challenging (Q 5.5)

					to go the way that surgeons and engineers go at the moment (Q 5.4)	involves multiple professions, so I think along that process you are going have lots of different teams of people working in stablishing the risk management strategy (Q 5.5)	

12
