# Supplementary material for: Application of quality by design for 3D printed bone prostheses and scaffolds
Source: PLoS One. 2018 Apr 12;13(4):e0195291. doi: 10.1371/journal.pone.0195291 (PMC5896968; doi:10.1371/journal.pone.0195291)
Supplement: S3 File — (PDF) [file pone.0195291.s003.pdf]

Data availability statement

Application of quality by design for 3D printed bone prostheses and scaffolds

Daniel. Martinez-Marquez[1], Ali. Mirnajafizadeh[2], Christopher P. Carty [3,4,5], Rodney A. Stewart[1*]

[1] School of Engineering, Griffith University, Gold Coast, Queensland, Australia

[2] Molecular Cell Biomechanics Laboratory, University of Berkeley, Berkeley, California, USA

[3] School of Allied Health Sciences and Innovations in Health Technology, Menzies Health Institute Queensland, Griffith University, Gold Coast, Queensland, Australia

[4] Centre for Musculoskeletal Research, Menzies Health Institute Queensland, Griffith University, Gold Coast, Queensland, Australia

[5] Queensland Children's Gait Laboratory, Queensland Paediatric Rehabilitation Service, Children's Health Queensland Hospital and Health Service, Brisbane, Queensland, Australia

* Rodney A. Stewart

Email: r.stewart@griffith.edu.au (RAS)

This study discussed and/or proposed an implementation of the Quality by Design (QbD) approach to custom 3D printed bone prostheses and scaffolds. The overall approach selected for this study is constructive research approach which is used to produce innovative solutions to practical problems [1] using managerial problem-solving techniques through the construction of models, diagrams, plans, and organizations [2]. However, it is important to note that this is a new research field and a niche industry which is still in its development stages. As a result, to gather the necessary data for this study, qualitative secondary data was the main data source needed to develop the adaptation of QbD system for custom 3D printed bone implants.

To gather the necessary data we performed an extensive qualitative systematic search of Science Direct, and Google Scholar using the Prisma statement guideline [3]. The systematic search was aimed at to identifying the main outcomes and benefits that QbD had provided to other studies research studies, and the different design and fabrication processes for custom 3D printed bone prostheses and scaffolds, including the risks associated with these processes.
The classification topics were: Application of QbD, QbD implemented steps; QBD tools used; Key output/conclusion; properties and requirements of porous scaffolds; medical image; biomaterials and surface treatments; study cases; 3D printing methods; 3D printing fabrication errors; design approaches; and performance simulation (finite element analysis; and joint kinematics simulation). The classified articles were thoroughly reviewed and analysed to get a deep understanding of the technologies and processes involved, in order to gather data related to the design and fabrication of patient-specific bone implants, and to identify risk factors related to their design and fabrication. Additionally, the reference list from collected papers was systematically reviewed to identify further items. Once all applicable literature was identified the tailored QbD approach adapted specifically for 3D printed bone implants was formulated.

To avoid risk of bias full-text screening was performed by independently D.M., R.S, C.C and A.M. Any discrepancy between the reviewers was resolved by a consensus meeting. The articles were classified based on their research objective to facilitate their analysis.

Note: The full list of studies included in this systematic search can be found in S5_Database File.

Search strategy

Systematic search Date: November 10th 2016

Search strategy for first question: Which are the design and manufacturing steps for custom 3D printed bone implants?

("Additive manufacturing" OR "3d printing" OR "rapid prototyping" OR "reverse engineering" OR "tissue engineering" OR "biomimetic") AND (scaffold OR custom OR custom?ed OR "patient specific") AND (implant* OR prosthes* OR design OR lattice)*

Table 1. Search strategy for first question, custom range 2000-2016 only journal papers

Data Base	Records identified
Google Scholar	18,200
Science Direct	20,460
Duplicates	16,900

Search strategy for second question: Which are the risks on product quality that can occur during the design and manufacturing of custom 3D printed bone implants?

("Additive manufacturing" OR "3d printing" OR "rapid prototyping" OR "reverse engineering") AND (accuracy OR defect OR metrology OR quality OR errors OR optimi?ation OR strategy OR rules)*

Table 2. Search strategy for second question, custom range 2000-2016 only journal papers

Data Base	Records identified
Google Scholar	17,200
Science Direct	17,125
Duplicates	8710

Systematic search Date: August 2th 2017

Search strategy for third question: what are the main reasons for the use of QbD?

(“*Quality by design”)*

Data Base	Records identified
Science Direct	1,057,344

Total records identified

Table 3. Total records identified

Data Base	Records identified	Total
Google Scholar	35,400	1,096,329
Science Direct	1,060,929	
Duplicates	25,610	**1,070,719**

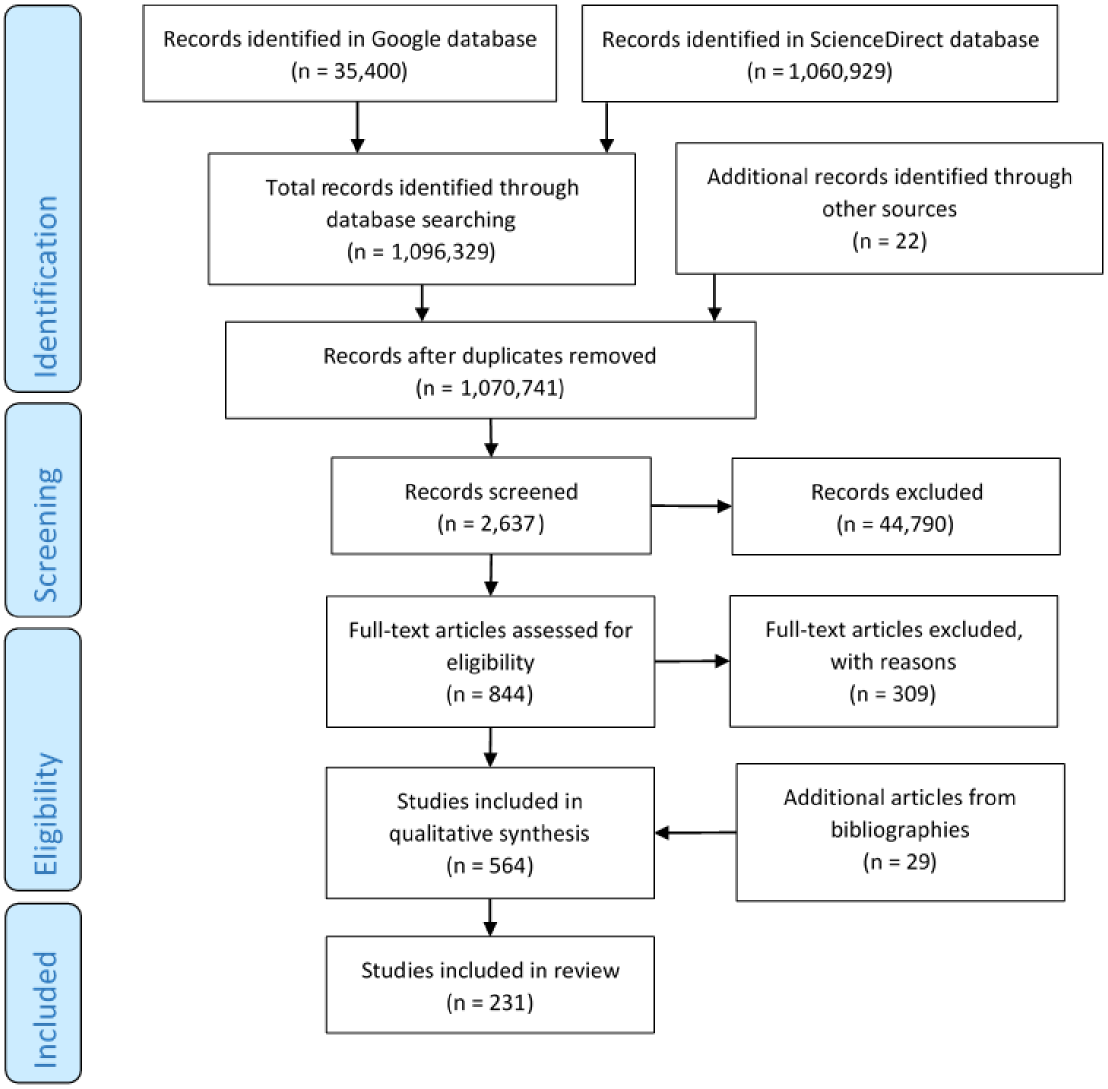

Figure 1. Search strategy and selection of the studies

References

1. Oyegoke A. The constructive research approach in project management research. International Journal of Managing Projects in Business. 2011;4(4):573-95. doi: 10.1108/17538371111164029.
2. Kasanen E, Lukka K, Siitonen A. The constructive approach in management accounting research. Journal of Management Accounting Research. 1993;5:243.
3. Liberati A, Altman DG, Tetzlaff J, Mulrow C, Gøtzsche PC, Ioannidis JP, et al. The PRISMA statement for reporting systematic reviews and meta-analyses of studies that evaluate health care interventions: explanation and elaboration. Annals of internal medicine. 2009;151(4):W-65-W-94.
4. ICH Harmonised Tripartite Guideline. Pharmaceutical development Q8 (R2). ICH Steering Committee, Step. 2009;4.
